# Diastereodivergent electrophilic trapping of α-boryl lithium derivatives

**DOI:** 10.3762/bjoc.22.68

**Published:** 2026-06-05

**Authors:** Tereza Pavlíčková, Noam Orbach, Ilan Marek

**Affiliations:** 1 Schulich Faculty of Chemistry and the Resnick Sustainability Center for Catalysis, Technion – Israel Institute of Technology, Haifa, 3200009, Israel

**Keywords:** α-boryl lithium, diastereoselectivity, (iodomethyl)cyclopropylboronic esters, ring-opening

## Abstract

α-Boryl carbanions are valuable organoboron intermediates, but controlling their stereoselective reactions remains challenging. Here, we examine the diastereoselective generation and trapping of α-boryl lithium species formed by ring opening of substituted iodomethylcyclopropanes. After lithium–iodine exchange and selective C–C bond cleavage, these intermediates react with a range of electrophiles to give boronic esters bearing vicinal tri- and tetrasubstituted stereocenters in good yields and high diastereoselectivities. Notably, substrates bearing alkyl substituents display the opposite sense of diastereoselectivity to previously studied aryl-substituted analogues. The results suggest that the stereochemical outcome is independent of the initial configuration at C2 and is instead dictated by conformational preferences of the α-boryl lithium intermediate. A simplified steric model is proposed in which lithium coordination induces a pseudo-chelated structure that controls facial selectivity during electrophilic trapping. These findings expand the synthetic utility of α-boryl lithium intermediates and provide insight into the origins of their diastereoselectivity.

## Introduction

**Scheme 1:**
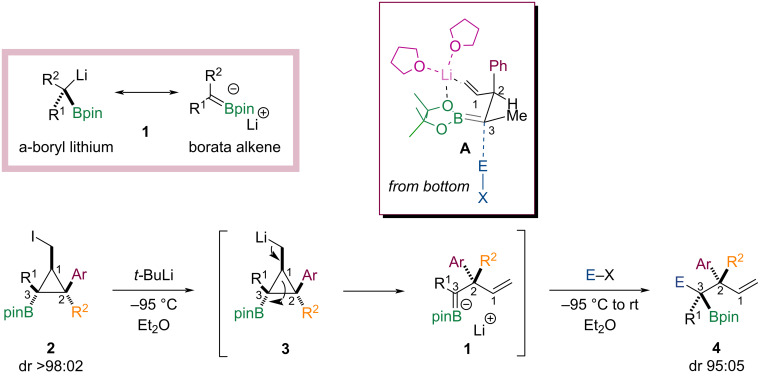
Synthesis and reactivity of α-boryl lithium species.

α-Boryl carbanions **1** (Scheme 1), also referred to as boron alkylidenes or borata alkenes, represent a fascinating class of organoboron intermediates characterized by the delocalization of electron density from the negatively charged carbon center toward the electron-deficient boron atom. This delocalization leads to the formation of a partial π C–B bond (Scheme 1) [1–2], which provides a unique stabilization to the carbanionic center and affects its reactivity towards electrophiles. In recent years, α-boryl carbanions have attracted significant attention as highly versatile intermediates capable of engaging in diverse bond-forming processes, including C–C, C–O and C–N bond formations [3–24]. Despite their growing utility, controlling the stereoselectivity in substitution reactions of α-boryl carbanions remains a formidable task [25–27]. The electronic character of these intermediates, especially for boronic ester derivatives that allow coordination between oxygen atoms and the metal counterion, complicates predictable stereochemical outcome. Developing strategies to harness the exceptional nature of these carbanions in a stereodefined manner would achieve the synthesis of polysubstituted organoboron compounds with adjacent stereocenters. The latter are highly valuable building blocks, given the pivotal role of organoboron compounds in contemporary chemistry as stable, yet reactive, intermediates that enable diverse catalytic and stereoselective transformations [28–33].

In this context, we have recently demonstrated that a broad range of arylated (iodomethyl)cyclopropylboronic ester **2** [34] undergo ring-opening reactions, followed by electrophilic trapping of the resulting α-boryl carbanion derivative **1** to afford product **4** as single diastereomers. Specifically, iodide **2** was subjected to *tert*-butyllithium (*t*-BuLi) in diethyl ether at −95 °C, efficiently generating cyclopropylmethyllithium **3** via lithium–iodine exchange. Subsequent selective C–C bond cleavage of **3**, proceeding exclusively through cleavage of the C1–C3 bond rather than the C1–C2 bond, provided direct access to α-boryl carbanion intermediate **1**. Reaction of **1** with a range of electrophiles furnished tertiary pinacol boranes with a well-defined adjacent stereocenter in excellent diastereoselectivity (Scheme 1).

Theoretical studies revealed that the electrophile preferentially approaches from the face opposite the coordinating lithium cation, with the lowest-energy transition state stabilized by a lithium cation–vinyl interaction (see **A** in Scheme 1) [34].

## Results and Discussion

**Scheme 2:**
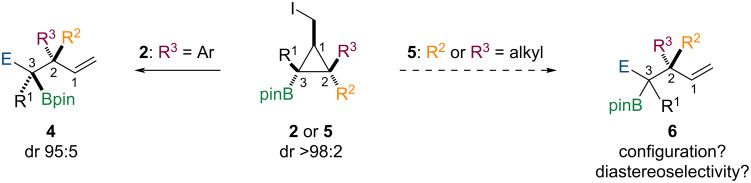
Diastereodivergent electrophilic trapping of α-boryl lithium derivatives.

The intricate interplay of the coordination and steric effects suggests that the adjacent stereocenter at C2 might play a key role in the conformational stability and the stereochemical outcome. We therefore set out to extend the scope of the transformation by probing how substitution at the C2 position of precursor **2** affects the diastereoselectivity of the reaction. We were particularly interested in examining cases with alkyl (**5**) instead of aryl substituents in **2** on C2 (Scheme 2).

**Figure 1:**
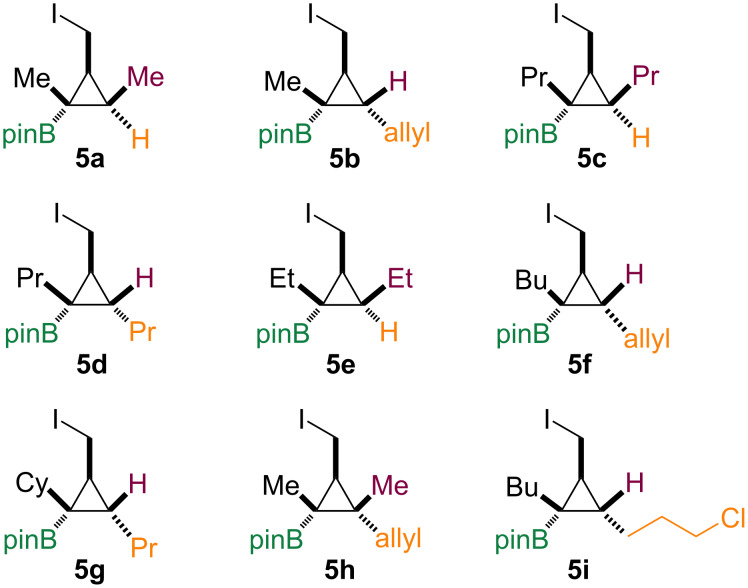
Cyclopropylmethyl iodide starting materials.

We initiated the investigation into the selectivity of the transformation with the synthesis of a library of cyclopropylmethyl iodides **5a**–**i**, using our previously reported methodology [35–37] (Figure 1).

**Scheme 3:**
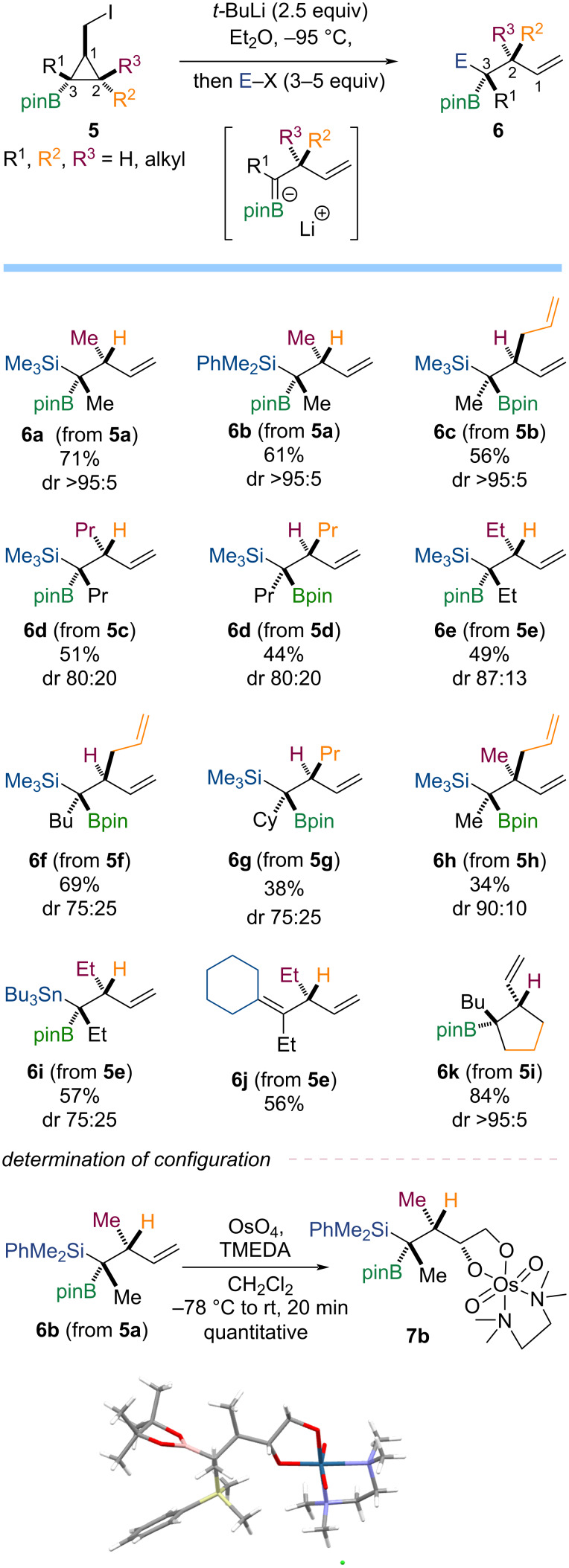
Reactivity of α-boryl lithium intermediates **5** possessing alkyl substituents at the allylic position.

When the substrate **5a** (R^1^ = R^3^ = Me, R^2^ = H) was subjected to the standard reaction conditions, the ring-opening process and trapping with Me_3_SiCl occurred selectively, affording **6a** in an excellent diastereomeric ratio (Scheme 3). Replacing Me_3_SiCl with the bulkier electrophile PhMe_2_SiCl did not alter the selectivity of the transformation, yielding **6b** as a single diastereomer (Scheme 3). The relative configuration of **6b** was determined by X-ray analysis of the osmate ester **7b**, derived from osmium-catalyzed dihydroxylation (Scheme 3) [38], and the other products were assigned by analogy. Interestingly, the relative configuration is reversed compared to the cases with aryl group as R^3^ (Scheme 2) [34].

The presence of an aliphatic substituent as R^2^ instead of R^3^ (R^3^ = H, R^2^ = allyl) has no influence on the diastereoselectivity, as **6c** was obtained with high selectivity. For both **6a**/**6b** and **6c**, the alkyl substituent, whether R^3^ (**6a**, **6b**) or R^2^ (**6c**), has a syn-relationship with the boronic ester group. Furthermore, performing the reaction with diastereomers **5c** (R^1^ = R^3^ = Pr, R^2^ = H) and **5d** (R^1^ = R^2^ = Pr, R^3^ = H), produced product **6d** with the same relative configuration (described as the two enantiomers to maintain an identical configuration of C2), albeit with moderate yields and diastereoselectivities.

This observation supports the hypothesis that the reaction outcome is independent of the initial relative configuration at C2 of the starting material. Increasing the length and branching of the R^1^ substituent (from Me, Et to Bu, and Cy) resulted in a slight decrease in diastereomeric ratios, providing **6e**–**g** in moderate yields and selectivities. To our delight, the reaction proceeded even when three alkyl substituents were present (**5h**, R^1^ = R^3^ = Me, R^2^ = allyl), providing compound **6h** bearing two adjacent fully substituted stereocenters (Scheme 3). Although the yield was relatively low (34%), the diastereomeric remained high *(*dr 90:10). The relative configuration of the major diastereomers of **6h** was assigned by analogy to **6c**, but it could not be definitively confirmed due to the inability to obtain suitable crystals for X-ray analysis. In addition to the nucleophilic substitution of the boryl lithium species with R_3_SiCl, a small range of alternative electrophiles was examined (Scheme 3). Incorporation of a tin-based electrophile furnished stannane **6i** in moderate yields with good diastereoselectivity. Reaction with cyclohexanone led to partial elimination, which proceeded to completion upon warming the reaction mixture to room temperature, affording the bora-Wittig product **6j** in moderate yield [39]. Furthermore, an intramolecular reaction was investigated: cyclopropane **5i** possessing a chloride as a leaving group underwent highly selective cyclization to deliver the stereodefined borylated cyclopentane **6k** in high 84% yield as a single diastereomer. The relative configuration of **6k** was unambiguously established by X-ray crystallographic analysis of an osmylated derivative (see Supporting Information File 1).

**Scheme 4:**
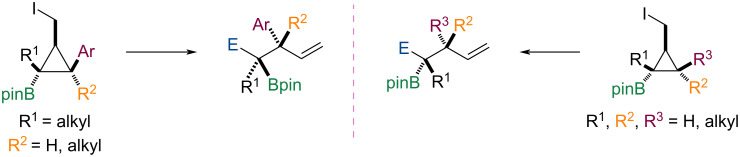
Effect of the nature of the substituent R^3^ on the diastereoselectivity.

As noted above, the diastereoselectivity observed for substrates bearing alkyl substituents at R^2^/R^3^ is opposite to that previously reported for aryl-substituted analogues (Scheme 4).

**Scheme 5:**
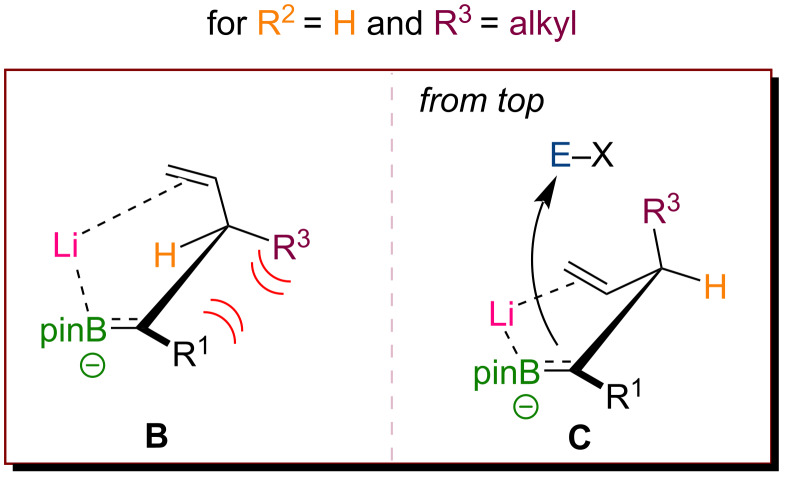
Mechanistic hypothesis to rationalize the diastereoselectivity.

The solvent-coordinated lithium alkylidene model used to rationalize the aryl series [34] does not account for the selectivity trend in the alkyl case, indicating that different stereochemical factors dominate in the absence of an aryl substituent. We therefore propose a simplified, steric-based model to account for the observed diastereoselectivity. Based on previous theoretical calculations, this model assumes initial coordination of both the vinyl group and the Bpin moiety to the lithium counterion. Within this constrained geometry, when R^2^ = H, R^3^ = alkyl, the diastereocontrol is then governed by the relative steric interactions present in these two possible conformers **B** and **C**. This conformational constraint rigidifies the system through formation of a pseudo-ring, leading to potential gauche interactions between R^1^ and R^3^ in conformer **B** (Scheme 5).

In conformer **C**, however, substituents R^1^ and R^3^ are further apart, which expose a free diastereotopic face above the plane of the molecule and allows the electrophile to react anti to the pseudo-chelated metallacyle. When R^2^ = alkyl and R^3^ = H, the corresponding enantiomeric conformer leads to the same diastereomer, as expected from the enantiomeric relationship (see **6d** from **5c** and **5d**, Scheme 3).

## Conclusion

In conclusion, this work establishes α-boryl lithium intermediates as valuable stereogenic platforms for conducting highly diastereoselective nucleophilic transformations. The combination of the anion-stabilizing capability of boron with strain-release-driven ring fragmentation provides exceptional chemo- and regioselectivity. The use of various electrophiles enabled the synthesis of acyclic boronic esters featuring vicinal tri- and tetrasubstituted stereocenters.

## Supporting Information

File 1 Experimental section, characterization data and copies of spectra.

## Data Availability

All data that supports the findings of this study is available in the published article and/or the supporting information of this article. The crystal data have been deposited in the Cambridge Crystallographic Data Centre (https://www.ccdc.cam.ac.uk/) deposition numbers CCDC 2537818 and 2537817.
