# Supplementary material for: Diastereodivergent electrophilic trapping of α-boryl lithium derivatives
Source: Beilstein J Org Chem. 2026 Jun 5;22:882–7. doi: 10.3762/bjoc.22.68 (PMC13245471; doi:10.3762/bjoc.22.68)
Supplement: File 1 — Experimental section, characterization data and copies of spectra. [file Beilstein_J_Org_Chem-22-882-s001.pdf]

BEILSTEIN JOURNAL OF ORGANIC CHEMISTRY

Supporting Information

for

Diastereodivergent electrophilic trapping of α-boryl lithium derivatives

Tereza Pavlíčková, Noam Orbach and Ilan Marek

Beilstein J. Org. Chem. **2026,** *22,* 882–887. doi:10.3762/bjoc.22.68

Experimental section, characterization data and copies of spectra

License and Terms: This is a supporting information file under the terms of the Creative Commons Attribution License (https://creativecommons.org/licenses/by/4.0). Please note that the reuse, redistribution and reproduction in particular requires that the author(s) and source are credited and that individual graphics may be subject to special legal provisions.
The license is subject to the *Beilstein Journal of Organic Chemistry* terms and conditions: (https://www.beilstein-journals.org/bjoc/terms)

Table of contents

1. General information S2

2. Synthesis of starting materials S3

1.1 Compounds **5a**, **5e**, **5f** and **5h** S3

1.2 Compounds **5b** and **5c** S3

1.3 Compounds **5d** and **5g** S5

1.4 Compound **5i** S8

3. Scope of ring opening/silylation S9

4. Determination of the stereochemical outcome S18

5. NMR spectra S21

6. References S84

1. General information

Air- and moisture-sensitive reactions were conducted in flame-dried glassware under a positive pressure of argon. Solvents were dried via distillation according to standard procedures (CH_2Cl_2, Et_3N) or used from a solvent purification system (THF, Et_2O; Pure-Solv.® Purification System) and stored at least 72 hours over activated 4 Å molecular sieves before usage. Commercially available reagents were used as purchased unless otherwise stated. Commercially available organolithium reagents were titrated twice against *N*-benzylbenzamide before usage. Thin-layer chromatography (TLC) was conducted with E. Merck silica gel 60 F254 pre-coated plates (0.25 mm) and visualized by exposure to UV light (254 nm) or stained with acidic *p*-anisaldehyde, cerium molybdate, or potassium permanganate solutions. Column chromatography was performed using Fluka silica gel 60 Å (40–63 mm, 230–400 mesh). PE (60–80 °C boiling range) was used for chromatographic separations. NMR spectra were recorded in $CDCl_3$ on a Bruker Avance instrument. ^{1}H NMR chemical shifts are provided using TMS as external standard (internal reference at δ = 7.26 ppm) and are reported as follows: chemical shift in ppm [multiplicity, coupling constant(s) *J* in Hz, integral]. The following abbreviations were used for peak multiplicities: br = broad, m = multiplet, s = singlet, d = doublet, t = triplet, q = quadruplet, quint = quintuplet, sext = sextuplet, sept = septuplet or combinations thereof. Carbon (^{13}C, APT) chemical shifts are referenced against the residual central solvent peak (δ = 77.16 ppm for $CDCl_3$) and are given in ppm. Boron (^{11}B), fluorine (^{19}F) and phosphorus (^{31}P) shifts are given in ppm using external calibration. High-resolution mass spectrometry (HRMS) was carried out on a FTICR instrument at the Mass Spectrometry Unit of the Schulich Faculty of Chemistry at the Technion – Israel Institute of Technology. Diastereomeric ratios (dr) were determined either by crude ^{1}H NMR (relaxation delay D1 = 6 s) or by GC/FID analysis using an Agilent Technologies 7820A GC with an Agilent Technologies 19091J-413 (30 m × 0.3 mm) column.

2. Synthesis of starting materials

Cyclopropyl iodides were prepared according to literature procedures.[1–3]

1.1 Compounds 5a, 5e, 5f and 5h

Compounds 5a, 5e, 5f, and 5h are known compounds; the experimental results were in agreement with the literature report.[1]

1.2 Compounds 5b and 5c

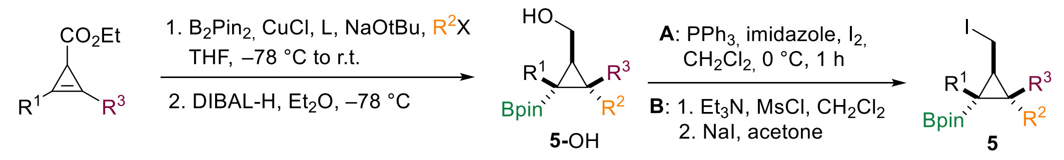

((1*S,2*R**,3*S**)-3-Allyl-2-methyl-2-(4,4,5,5-tetramethyl-1,3,2-dioxaborolan-2-yl)cyclopropyl)methanol (5b-OH)**

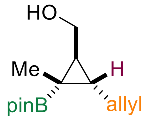

Chemical Formula: $C_{11}H_{20}BO_3$
Molecular Weight: 211,09

Prepared according to a literature procedure[1,2] from ethyl 2-methylcycloprop-2-ene-1-carboxylate (456 mg, 3.63 mmol).

Yield: 410 mg (1.63 mmol, 45% over two steps, dr >95:05 as determined by ^{1}H NMR spectroscopy) as a colorless oil.

R*f* = 0.50 (PE/Et_2O 1:1).

^{1}H NMR (400 MHz, $CDCl_3$) δ 5.81 (ddt, *J* = 16.4, 11.5, 5.9 Hz, 1H), 5.00 (dd, *J* = 17.2, 1.6 Hz, 1H), 4.92 (d, *J* = 10.3 Hz, 1H), 3.73 (dd, *J* = 11.4, 6.3 Hz, 1H), 3.53 (dd, *J* = 11.4, 8.6 Hz, 1H), 2.15 (hept, *J* = 8.3 Hz, 2H), 1.97 (s, 1H), 1.24 – 1.12 (m, 1H), 1.17 (s, 6H), 1.16 (s, 6H), 1.07 (s, 3H), 0.61 (q, *J* = 7.1 Hz, 1H) ppm.

^{13}C NMR (101 MHz, $CDCl_3$) δ 138.6, 114.3, 83.2, 62.5, 34.1, 31.6, 31.0, 25.1, 24.6, 15.8 ppm.
Note: Carbon atom attached to boron is not visible due to quadrupolar relaxation.

^{11}B NMR (128 MHz, $CDCl_3$): δ 33.1 ppm.

HRMS (TOF ESI+) m/z: calcd. for $C_{14}H_{25}BO_3Na^+$ [*M*+Na]$^+$: 275.1974, found: 275.1796.

2-((1*R,2*S**,3*S**)-2-Allyl-3-(iodomethyl)-1-methylcyclopropyl)-4,4,5,5-tetramethyl-1,3,2-dioxaborolane (5b)**

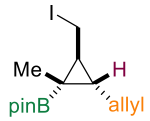

Chemical Formula: $C_{14}H_{24}BIO_2$
Molecular Weight: 362.06

Prepared according to a literature procedure **A**[2] from **5b**-OH (101 mg, 400 µmol).

Yield: 116 mg (320 µmol, 80%; dr >95:05 as determined by ^{1}H NMR spectroscopy using *p*-xylene as internal standard) as a colorless oil.

^{1}H NMR (400 MHz, $CDCl_3$) δ 5.87 (ddt, *J* = 16.4, 10.4, 6.0 Hz, 1H), 5.15 – 4.89 (m, 2H), 3.39 (dd, *J* = 9.7, 8.2 Hz, 1H), 3.28 – 3.19 (m, 1H), 2.37 – 2.24 (m, 1H), 2.21 – 2.10 (m, 1H), 1.53 (td, *J* = 8.2, 5.6 Hz, 1H), 1.22 (s, 6H), 1.20 (s, 6H), 1.11 (s, 3H), 0.66 (q, *J* = 6.6 Hz, 1H) ppm.

^{13}C NMR (101 MHz, $CDCl_3$) δ 138.2, 114.4, 83.3, 37.5, 34.0, 32.9, 25.1, 24.7, 14.9, 9.0 ppm.
Note: Carbon atom attached to boron is not visible due to quadrupolar relaxation.

^{11}B NMR (128 MHz, $CDCl_3$): δ 33.3 ppm.

HRMS (TOF ESI+) m/z: calcd. for $C_{14}H_{24}BIO_2^+$ [*M*+H]$^+$: 363.0993, found: 363.0997.

((1*S,2*R**,3*R**)-2,3-Dipropyl-2-(4,4,5,5-tetramethyl-1,3,2-dioxaborolan-2-yl)cyclopropyl)methanol (5c-OH)**

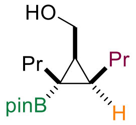

Chemical Formula: $C_{16}H_{31}BO_3$
Molecular Weight: 282,23

Prepared according to a literature procedure[1,2] from ethyl 2,3-dipropylcycloprop-2-ene-1-carboxylate (968 mg, 4.93 mmol).

Yield: 710 mg (2.51 mmol, 51% over two steps, dr >95:05 as determined by ^{1}H NMR spectroscopy) as a colorless oil.

R*f* = 0.40 (PE/Et_2O 1:1).

^{1}H NMR (400 MHz, $CDCl_3$) δ 3.78 – 3.63 (m, 2H), 1.64 – 1.23 (m, 10H), 1.19 (s, 12H), 1.07 – 0.99 (m, 1H), 0.92 (t, *J* = 6.7 Hz, 3H), 0.89 (t, *J* = 6.7 Hz, 3H) ppm.

^{13}C NMR (101 MHz, $CDCl_3$) δ 83.0, 59.7, 27.2, 26.3, 25.9, 24.7, 24.7, 24.7, 23.6, 23.1, 15.0, 14.3 ppm.
Note: Carbon atom attached to boron is not visible due to quadrupolar relaxation.

^{11}B NMR (128 MHz, $CDCl_3$): δ 33.2 ppm.

HRMS (APCI+) m/z: calcd. for $C_{16}H_{31}BIO_2^+$ [*M*–H_2O+H]$^+$: 265.2333, found: 265.2336.

2-((1*R,2*S**,3*R**)-2-(Iodomethyl)-1,3-dipropylcyclopropyl)-4,4,5,5-tetramethyl-1,3,2-dioxaborolane (5c)**

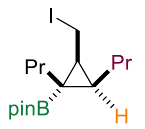

Chemical Formula: $C_{16}H_{30}BIO_2$
Molecular Weight: 392.13

Prepared according to a literature procedure **B**[3] from **5c**-OH (141 mg, 500 µmol).

Yield: 196 mg (500 µmol, quantitative over two steps; dr >95:05 as determined by ^{1}H NMR spectroscopy using *p*-xylene as internal standard) as a colorless oil.

^{1}H NMR (400 MHz, $CDCl_3$) δ 3.42 (dd, *J* = 9.8, 6.5 Hz, 1H), 3.20 (t, *J* = 10.0 Hz, 1H), 1.63 (ddd, *J* = 10.2, 8.5, 6.5 Hz, 1H), 1.56 – 1.22 (m, 8H), 1.19 (s, 6H), 1.19 (s, 6H), 1.09 (dt, *J* = 8.6, 6.5 Hz, 1H), 0.94 (t, *J* = 6.9 Hz, 3H), 0.90 (t, *J* = 7.2 Hz, 3H) ppm.

^{13}C NMR (101 MHz, $CDCl_3$) δ 83.1, 28.1, 27.6, 26.6, 24.7, 24.6, 24.6, 23.5, 22.9, 14.9, 14.3, 5.7 ppm. *Note*: Carbon atom attached to boron is not visible due to quadrupolar relaxation.

^{11}B NMR (128 MHz, $CDCl_3$): δ 32.8 ppm.

HRMS (APCI+) m/z: calcd. for $C_{16}H_{31}BIO_2^+$ [*M*+H]$^+$: 393.1456, found: 393.1479.

1.3 Compounds 5d and 5g

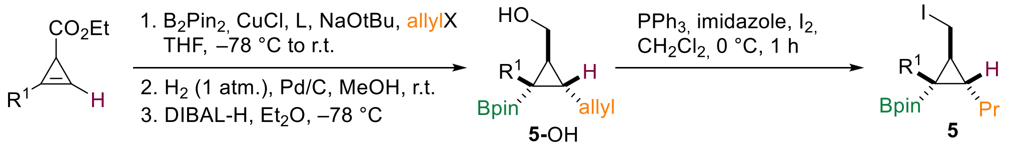

Step 1: Borylation was carried out according to a literature procedure and allyl phosphate as electrophile.[1]

Step 2: The above prepared pinacol borane was dissolved in MeOH (0.1 M) and Pd on charcoal (0.1 equiv w/w) was added. The mixture was stirred at room temperature under positive pressure of hydrogen (one balloon) for 4 h. The suspension was subsequently purged with argon, the solids were filtered and the mixture was concentrated under reduced pressure to give crude product.

Step 3: The crude ester was reduced according to a literature procedure.[1,2]

((1*S,2*R**,3*S**)-2,3-Dipropyl-2-(4,4,5,5-tetramethyl-1,3,2-dioxaborolan-2-yl)cyclopropyl)methanol (5d-OH)**

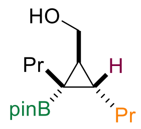

Chemical Formula: $C_{16}H_{31}BO_3$
Molecular Weight: 282,23

Prepared from ethyl 2-propylcycloprop-2-ene-1-carboxylate (342 mg, 2.22 mmol).

Yield: 345 mg (1.22 mmol, 52% over three steps, dr >95:05 as determined by ^{1}H NMR spectroscopy) as a white solid.

R*f* = 0.35 (PE/Et_2O 1:1).

^{1}H NMR (400 MHz, $CDCl_3$) δ 3.74 – 3.58 (m, 2H), 1.55 – 1.45 (m, 1H), 1.35 (td, *J* = 16.7, 8.6 Hz, 8H), 1.22 (s, 6H), 1.19 (s, 6H), 1.17 – 1.09 (m, 1H), 0.88 (t, *J* = 7.6 Hz, 3H), 0.86 (t, *J* = 7.6 Hz, 3H), 0.59 (q, *J* = 6.4 Hz, 1H) ppm.

^{13}C NMR (101 MHz, $CDCl_3$) δ 83.1, 63.0, 34.1, 32.7, 31.7, 31.2, 25.2, 24.6, 23.4, 23.2, 14.8, 14.0 ppm. *Note*: Carbon atom attached to boron is not visible due to quadrupolar relaxation.

^{11}B NMR (128 MHz, $CDCl_3$): δ 33.1 ppm.

HRMS (APCI+) m/z: calcd. for $C_{16}H_{30}BO_2^+$ [*M*–H_2O+H]$^+$: 265.2333, found: 265.2323.

2-((1*R,2*S**,3*S**)-2-(Iodomethyl)-1,3-dipropylcyclopropyl)-4,4,5,5-tetramethyl-1,3,2-dioxaborolane (5d)**

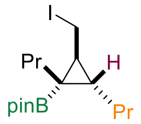

Chemical Formula: $C_{16}H_{30}BIO_2$
Molecular Weight: 392.13

Prepared according to a literature procedure[2] from **5d**-OH (115 mg, 410 µmol).

Yield: 123 mg (315 µmol, 77%; dr >95:05 as determined by ^{1}H NMR spectroscopy using *p*-xylene as internal standard) as a colorless oil.

^{1}H NMR (400 MHz, $CDCl_3$) δ 3.44 (dd, *J* = 9.5, 6.5 Hz, 1H), 3.21 (t, *J* = 9.8 Hz, 1H), 2.09 (t, *J* = 7.1 Hz, 1H), 1.50 – 1.41 (m, 2H), 1.41 – 1.29 (m, 6H), 1.20 (s, 6H), 1.18 (s, 6H), 0.88 (t, *J* = 7.6 Hz, 3H), 0.86 (t, *J* = 7.3 Hz, 3H), 0.58 (q, *J* = 6.5 Hz, 1H) ppm.

^{13}C NMR (101 MHz, $CDCl_3$) δ 83.1, 37.3, 33.8, 33.6, 32.6, 25.2, 24.5, 23.3, 23.2, 14.7, 14.1, 10.4 ppm. *Note*: Carbon atom attached to boron is not visible due to quadrupolar relaxation.

^{11}B NMR (128 MHz, $CDCl_3$): δ 32.8 ppm.

HRMS (APCI+) m/z: calcd. for $C_{16}H_{31}BIO_2^+$ [*M*+H]$^+$: 393.1456, found: 393.1489.

((1*S,2*R**,3*S**)-2-Cyclohexyl-3-propyl-2-(4,4,5,5-tetramethyl-1,3,2-dioxaborolan-2-yl)cyclopropyl)methanol (5g-OH)**

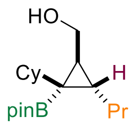

Chemical Formula: $C_{19}H_{35}BO_3$
Molecular Weight: 322,30

Prepared from ethyl 2-cyclohexylcycloprop-2-ene-1-carboxylate (971 mg, 5.00 mmol).

Yield: 451 mg (1.4 mmol, 28% over three steps, dr >95:05 as determined by ^{1}H NMR spectroscopy) as a white solid.

R*f* = 0.40 (PE/Et_2O 1:2).

^{1}H NMR (400 MHz, $CDCl_3$) δ 3.70 (dd, *J* = 11.2, 7.7 Hz, 1H), 3.61 (dd, *J* = 11.3, 7.0 Hz, 1H), 1.89 (s, 1H), 1.71 (q, *J* = 7.5 Hz, 2H), 1.67 – 1.44 (m, 4H), 1.35 (ddd, *J* = 18.6, 13.4, 7.1 Hz, 4H), 1.30 – 1.02 (m, 5H), 1.19 (s, 6H), 1.16 (s, 6H), 0.86 (t, *J* = 6.9 Hz, 3H), 0.64 (ddd, *J* = 11.9, 8.3, 3.5 Hz, 1H), 0.52 (dt, *J* = 11.4, 5.5 Hz, 1H) ppm.

^{13}C NMR (101 MHz, $CDCl_3$) δ 82.8, 62.5, 41.9, 33.4, 32.6, 31.8, 31.6, 30.2, 27.4, 27.1, 26.6, 25.4, 24.4, 23.3, 14.0 ppm.
Note: Carbon atom attached to boron is not visible due to quadrupolar relaxation.

^{11}B NMR (128 MHz, $CDCl_3$): δ 32.7 ppm.

HRMS (APCI+) m/z: calcd. for $C_{19}H_{34}BO_2^+$ [*M*–H_2O+H]$^+$: 305.2655, found: 305.2646.

2-((1*R,2*S**,3*S**)-1-Cyclohexyl-2-(iodomethyl)-3-propylcyclopropyl)-4,4,5,5-tetramethyl-1,3,2-dioxaborolane (5g)**

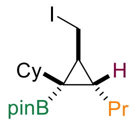

Chemical Formula: $C_{19}H_{34}BIO_2$
Molecular Weight: 432.19

Prepared according to a literature procedure[2] from 5g-OH (121 mg, 375 µmol).

Yield: 121 mg (281 µmol, 75%; dr >95:05 as determined by ^{1}H NMR spectroscopy using *p*-xylene as internal standard) as a colorless oil.

^{1}H NMR (400 MHz, $CDCl_3$) δ 3.59 (dd, *J* = 9.5, 5.5 Hz, 1H), 3.26 – 3.16 (m, 1H), 1.84 – 1.60 (m, 5H), 1.55 (dq, *J* = 13.8, 7.0 Hz, 2H), 1.48 – 1.32 (m, 4H), 1.33 – 1.26 (m, 2H), 1.21 (s, 6H), 1.19 (s, 6H), 1.12 (t, *J* = 12.6 Hz, 3H), 0.90 (t, *J* = 7.1 Hz, 3H), 0.76 – 0.64 (m, 1H), 0.56 (q, *J* = 5.9 Hz, 1H) ppm.

^{13}C NMR (101 MHz, $CDCl_3$) δ 83.0, 42.3, 36.3, 33.7, 33.1, 33.1, 31.4, 27.4, 27.1, 26.5, 25.4, 24.5, 23.2, 14.2, 10.7 ppm.
Note: Carbon atom attached to boron is not visible due to quadrupolar relaxation.

^{11}B NMR (128 MHz, $CDCl_3$): δ 32.5 ppm.

HRMS (APCI+) m/z: calcd. for $C_{19}H_{34}BO_2^+$ [*M*–HI+H]$^+$: 305.2646, found: 305.2641.

1.4 Compound 5i

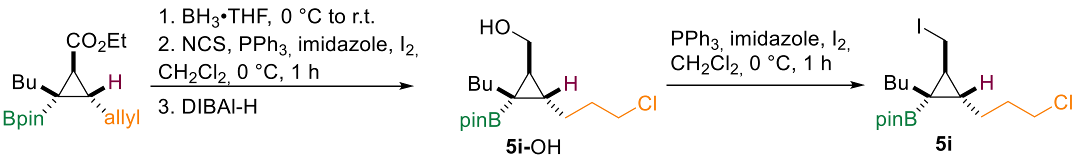

((1*S,2*R**,3*S**)-2-Butyl-3-(3-chloropropyl)-2-(4,4,5,5-tetramethyl-1,3,2-dioxaborolan-2-yl)cyclopropyl)methanol (5i-OH)**

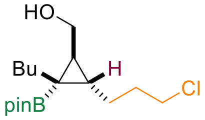

Chemical Formula: $C_{17}H_{32}BClO_3$
Molecular Weight: 330,70

Step 1: A flame-dried Schlenk flask was charged with a solution of pinacolborane **S1**[1] (336 mg, 1.00 mmol) in dry THF (5.0 mL) under argon atmosphere. The solution was cooled to 0 °C and BH_3•THF (1.5 mL, 1.0 M solution in THF) was added dropwise. The mixture was stirred for 1 h when TLC analysis indicated full consumption of **S1**. The mixture was quenched by a dropwise addition of MeOH at 0 °C, until the evolution of hydrogen gas ceased. Sodium perborate (1.09 g, 4.00 mmol) was added, followed by a dropwise addition of water (1.0 mL) at 0 °C and the mixture was stirred for 1 h at this temperature. Na_2SO_3 satd. solution (10 mL) was added, the layers were separated and the aqueous one was extracted with EtOAc (3 × 10 mL). The organic layers were washed with brine, dried over Na_2SO_4, filtered, the filtrate was concentrated under reduced pressure and the crude residue was purified by silica gel column chromatography (PE/EtOAc 99:1 to 7:3) to give the desired alcohol.

Step 2: A solution of PPh_3 (249 mg, 948 µmol) and *N*-chlorosuccinimide (116 mg, 869 µmol) in dry DCM (5 mL) was stirred for 30 min, then cooled to 0 °C and imidazole (11 mg, 157 µmol) was added. A solution of the above prepared ester in dry DCM (2 mL) was added dropwise, the mixture was stirred for 2 h at 0 °C and then overnight at room temperature. It was diluted with PE (20 mL), filtered over a short plug of silica gen and washed with 5% Et_2O in PE. The volatiles were removed under reduced pressure and the crude residue was used in the next step without further purification.

Step 3: The ester was reduced according to a literature procedure.[1,2]

Yield: 100 mg (302 µmol, 30% over three steps, dr >95:05 as determined by ^{1}H NMR spectroscopy) as a colorless oil.

R*f* = 0.65 (PE/Et_2O 1:1).

^{1}H NMR (400 MHz, $CDCl_3$) δ 3.75 – 3.58 (m, 2H), 3.54 (t, *J* = 6.7 Hz, 2H), 1.81 (hept, *J* = 7.3 Hz, 2H), 1.65 – 1.52 (m, 2H), 1.52 – 1.40 (m, 1H), 1.37 – 1.11 (m, 6H), 1.21 (s, 6H), 1.17 (s, 6H), 0.86 (t, *J* = 6.9 Hz, 3H), 0.58 (q, *J* = 7.1 Hz, 1H) ppm.

^{13}C NMR (101 MHz, $CDCl_3$) δ 83.2, 62.7, 45.0, 33.2, 32.3, 31.6, 31.2, 30.3, 28.0, 25.2, 24.6, 23.2, 14.2 ppm.
Note: Carbon atom attached to boron is not visible due to quadrupolar relaxation.

^{11}B NMR (128 MHz, $CDCl_3$): δ 32.9 ppm.

HRMS (TOF ESI+) m/z: calcd. for $C_{17}H_{33}BClNaO_3^+$ [*M*+Na]$^+$: 353.2031, found: 353.2025.

2-((1*R,2*S**,3*S**)-1-Butyl-2-(3-chloropropyl)-3-(iodomethyl)cyclopropyl)-4,4,5,5-tetramethyl-1,3,2-dioxaborolane (5i)**

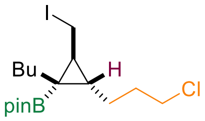

Chemical Formula: $C_{17}H_{31}BClIO_2$
Molecular Weight: 440.60

Prepared according to a literature procedure[2] from **5i**-OH (100 mg, 302 μmol).

Yield: 79 mg (180 μmol, 60%; *dr* > 95:05 as determined by ^{1}H NMR spectroscopy using *p*-xylene as internal standard) as a colorless oil.

^{1}H NMR (400 MHz, $CDCl_3$) δ 3.62 – 3.52 (m, 2H), 3.49 (dd, *J* = 9.6, 6.1 Hz, 1H), 3.19 (t, *J* = 10.0 Hz, 1H), 1.99 – 1.76 (m, 2H), 1.70 – 1.54 (m, 2H), 1.55 – 1.44 (m, 2H), 1.39 – 1.23 (m, 5H), 1.23 (s, 6H), 1.21 (s, 6H), 0.88 (t, *J* = 6.9 Hz, 3H), 0.59 (td, *J* = 7.2, 5.5 Hz, 1H) ppm.

^{13}C NMR (101 MHz, $CDCl_3$) δ 83.3, 45.1, 36.1, 33.5, 32.9, 32.4, 31.1, 27.8, 25.2, 24.6, 23.2, 14.2, 9.9 ppm. *Note*: Carbon atom attached to boron is not visible due to quadrupolar relaxation.

^{11}B NMR (128 MHz, $CDCl_3$): δ 32.9 ppm.

HRMS (APCI+) m/z: calcd. for $C_{17}H_{32}BClIO_2^+$ [*M*+H]$^+$: 441.1229, found: 441.1238.

3. Scope of ring opening/silylation

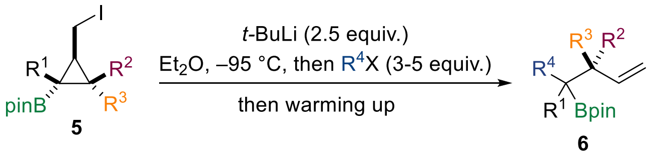

General procedure for Li–halogen exchange-mediated ring opening/silylation (GP1):[3] A flame-dried Schlenk flask was charged with dry Et_2O (0.2 M with respect to **5**, typically 1 mL) under argon and a solution of *t*-BuLi (2.5 equiv, typically 1.4 M in pentane, freshly titrated before use against *N*-benzyl benzamide)[4] was added at −78 °C. The mixture was cooled to −95 °C with good stirring and a solution of iodide **5** (typically 200 μmol) in dry Et_2O (0.2 M) was added via a cannula. A bright yellow precipitate formed. Electrophile (3–5 equiv) was added and the mixture was stirred with warming up to the required temperature while monitored by TLC against the proton-quench product (R*f* ≈ 0.45 vs. 0.70 for silylated compounds **6**). Completion was accompanied by decoloration and was typically reached after 30–40 min of stirring at −30 °C. The reaction mixture was subsequently quenched with MeOH (10 equiv), diluted with Et_2O and filtered over a short plug of silica gel. The crude residue was purified by flash column chromatography (silica gel, gradient PE/Et_2O 999:1 to 99:1; majority of compounds elute at 997:3) to obtain title compounds **6**.

Trimethyl((2*R,3*R**)-3-methyl-2-(4,4,5,5-tetramethyl-1,3,2-dioxaborolan-2-yl)pent-4-en-2-yl)silane (6a)**

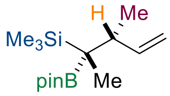

Chemical Formula: $C_{15}H_{31}BO_2Si$
Molecular Weight: 282.31

Prepared according to general procedure **GP1** from **5a** (67 mg, 200 μmol) and Me_3SiCl (63 μL, 500 μmol).

Yield: 40 mg (142 μmol, 71%, dr >95:05 as determined by ^1H NMR spectroscopy) as a colorless oil.

R*f* = 0.65 (PE/Et_2O 40:1).

^{1}H NMR (400 MHz, $CDCl_3$) δ 5.84 (ddd, *J* = 17.1, 10.1, 9.1 Hz, 1H), 4.95 (dd, *J* = 17.1, 1.4 Hz, 1H), 4.89 (dd, *J* = 10.1, 2.0 Hz, 1H), 2.61 – 2.50 (m, 1H), 1.23 (s, 12H), 1.04 (d, *J* = 6.9 Hz, 3H), 0.97 (s, 3H), 0.03 (s, 9H) ppm.

^{13}C NMR (101 MHz, $CDCl_3$) δ 143.6, 113.0, 82.9, 41.3, 25.4, 25.3, 19.7, 11.8, -1.6 ppm.
Note: Carbon atom attached to boron is not visible due to quadrupolar relaxation.

^{29}Si NMR (80 MHz, $CDCl_3$) δ 4.6 ppm.

^{11}B NMR (128 MHz, $CDCl_3$): δ 34.5 ppm.

HRMS (APCI+) m/z: calcd. for $C_{15}H_{32}BO_2Si^+$ [*M*+H]$^+$: 283.2259, found: 283.2254.

Trimethyl((2*R,3*R**)-3-methyl-2-(4,4,5,5-tetramethyl-1,3,2-dioxaborolan-2-yl)pent-4-en-2-yl)silane (6b)**

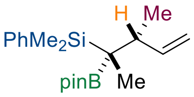

Chemical Formula: $C_{20}H_{33}BO_2Si$
Molecular Weight: 344,38

Prepared according to general procedure **GP1** from **5a** (61 mg, 180 μmol) and $Me_2PhSiCl$ (92 μL, 540 μmol).

Yield: 38 mg (110 μmol, 61%, dr >95:05 as determined by ^1H NMR spectroscopy) as a colorless oil.

R*f* = 0.45 (PE/Et_2O 40:1).

^{1}H NMR (400 MHz, $CDCl_3$) δ 7.59 (m, 2H), 7.36 – 7.26 (m, 3H), 5.79 (ddd, *J* = 17.2, 10.0, 8.9 Hz, 1H), 5.00 – 4.74 (m, 2H), 2.56 (p, *J* = 7.0 Hz, 1H), 1.19 (s, 6H), 1.09 (s, 6H), 1.09 (s, 3H), 1.03 (d, *J* = 6.9 Hz, 3H), 0.39 (s, 3H), 0.33 (s, 3H) ppm.

^{13}C NMR (101 MHz, $CDCl_3$) δ 143.1, 139.1, 135.1, 128.7, 127.3, 113.5, 83.0, 41.2, 25.4, 25.2, 19.5, 12.4, -2.6, -3.0 ppm.
Note: Carbon atom attached to boron is not visible due to quadrupolar relaxation.

^{29}Si NMR (80 MHz, $CDCl_3$) δ -0.8 ppm.

^{11}B NMR (128 MHz, $CDCl_3$): δ 34.5 ppm.

HRMS (APCI+) m/z: calcd. for $C_{20}H_{34}BO_2Si^+$ [*M*+H]$^+$: 345.2416, found: 345.2415.

Trimethyl((2*S,3*S**)-2-(4,4,5,5-tetramethyl-1,3,2-dioxaborolan-2-yl)-3-vinylhex-5-en-2-yl)silane (6c)**

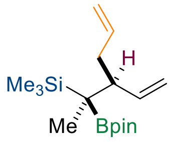

Chemical Formula: $C_{17}H_{33}BO_2Si$
Molecular Weight: 308.34

Prepared according to general procedure **GP1** from **5b** (116 mg, 320 µmol) and Me_3SiCl (203 µL, 1.6 mmol).

Yield: 55 mg (178 µmol, 56%, dr >95:05 as determined by ^{1}H NMR spectroscopy) as a colorless oil.

R*f* = 0.60 (PE/Et_2O 40:1).

^{1}H NMR (400 MHz, $CDCl_3$) δ 5.81 – 5.69 (m, 1H), 5.65 (dt, *J* = 17.1, 9.9 Hz, 1H), 4.99 (dd, *J* = 10.2, 2.2 Hz, 1H), 4.96 – 4.86 (m, 3H), 2.40 (td, *J* = 10.7, 2.6 Hz, 1H), 2.21 (td, *J* = 12.4, 7.0 Hz, 1H), 2.01 (ddt, *J* = 13.6, 7.1, 1.3 Hz, 1H), 1.24 (s, 12H), 1.01 (s, 3H), 0.02 (s, 9H) ppm.

^{13}C NMR (101 MHz, $CDCl_3$) δ 140.4, 138.6, 115.4, 114.8, 83.0, 47.7, 38.6, 25.4, 11.7, -1.6 ppm.
Note: Carbon atom attached to boron is not visible due to quadrupolar relaxation.

^{29}Si NMR (80 MHz, $CDCl_3$) δ 4.4 ppm.

^{11}B NMR (128 MHz, $CDCl_3$): δ 34.5 ppm.

HRMS (APCI+) m/z: calcd. for $C_{17}H_{33}BO_2Si^+$ [*M*+H]$^+$: 309.2422, found: 309.2421.

Trimethyl((4*R,5*R**)-4-(4,4,5,5-tetramethyl-1,3,2-dioxaborolan-2-yl)-5-vinyloctan-4-yl)silane (6d)**

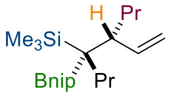

Chemical Formula: $C_{19}H_{39}BO_2Si$
Molecular Weight: 338.41

Prepared according to general procedure **GP1** from **5c** (141 mg, 360 µmol) and Me_3SiCl (137 µL, 1.1 mmol).

Yield: 62 mg (183 µmol, 51%) as a partially separable mixture of diastereomers (*dr* 80:20 as determined by ^{1}H NMR spectroscopy) as a colorless oil.

Prepared according to general procedure **GP1** from **5d** (118 mg, 300 µmol) and Me_3SiCl (190 µL, 1.5 mmol).

Yield: 45 mg (133 µmol, 44%) as a partially separable mixture of diastereomers (dr 80:20 as determined by ^{1}H NMR spectroscopy) as a colorless oil.

R*f* = 0.65 (PE/Et_2O 40:1).

Major diastereomer:
^{1}H NMR (400 MHz, $CDCl_3$) δ 5.73 (dt, *J* = 17.1, 10.0 Hz, 1H), 4.97 (dd, *J* = 10.2, 2.3 Hz, 1H), 4.89 (dd, *J* = 17.1, 2.3 Hz, 1H), 2.41 – 2.24 (m, 1H), 1.63 – 1.34 (m, 6H), 1.23 – 1.21 (m, 1H), 1.22 (s, 6H), 1.21 (s, 6H), 1.16 – 1.02 (m, 1H), 0.88 – 0.83 (m, 6H), 0.06 (s, 9H) ppm.

^{13}**C NMR** (101 MHz, $CDCl_3$) δ 143.0, 114.8, 82.5, 46.3, 33.7, 32.4, 25.6, 25.2, 21.07, 20.5, 15.5, 14.3, 0.8 ppm.
Note: Carbon atom attached to boron is not visible due to quadrupolar relaxation.

^{29}**Si NMR** (80 MHz, $CDCl_3$) δ 4.0 ppm.

Minor diastereomer:
^{1}**H NMR** (400 MHz, $CDCl_3$) δ 5.92 (dt, *J* = 17.1, 10.1 Hz, 1H), 4.97 (dd, *J* = 10.2, 2.3 Hz, 1H), 4.93 – 4.82 (m, 1H), 2.41 – 2.24 (m, 1H), 1.63 – 1.34 (m, 6H), 1.23 – 1.21 (m, 1H), 1.21 (s, 6H), 1.20 (s, 6H), 1.16 – 1.02 (m, 1H), 0.88 – 0.83 (m, 6H), 0.07 (s, 9H) ppm.

^{13}**C NMR** (101 MHz, $CDCl_3$) δ 141.9, 114.7, 82.5, 46.6, 36.4, 32.3, 25.4, 25.2, 21.6, 21.12, 15.6, 14.2, 0.4 ppm.
Note: Carbon atom attached to boron is not visible due to quadrupolar relaxation.

^{29}**Si NMR** (80 MHz, $CDCl_3$) δ 4.2 ppm.

^{11}**B NMR** (128 MHz, $CDCl_3$): δ 34.4 ppm.

HRMS (APCI+) m/z: calcd. for $C_{19}H_{40}BO_2Si^+$ [*M*+H]$^+$: 339.2885, found: 339.2884.

((3*R,4*R**)-4-Ethyl-3-(4,4,5,5-tetramethyl-1,3,2-dioxaborolan-2-yl)hex-5-en-3-yl)trimethylsilane (6e)**

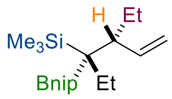

Chemical Formula: $C_{17}H_{35}BO_2Si$
Molecular Weight: 310,36

Prepared according to general procedure **GP1** from **5b** (100 mg, 275 µmol) and Me_3SiI (174 µL, 1.37 mmol).

Yield: 42 mg (137 µmol, 49%) as an inseparable mixture of diastereomers (dr 87:13 as determined by ^{1}H NMR spectroscopy) as a colorless oil.

R*f* = 0.65 (PE/Et_2O 40:1).

Major diastereomer:
^{1}**H NMR** (400 MHz, $CDCl_3$) δ 5.69 (dt, *J* = 17.1, 10.0 Hz, 1H), 5.00 (dd, *J* = 10.2, 2.3 Hz, 1H), 4.92 (dd, *J* = 17.1, 2.3 Hz, 1H), 2.22 – 2.12 (m, 1H), 1.72 (dq, *J* = 14.9, 7.5 Hz, 1H), 1.64 – 1.53 (m, 1H), 1.50 (ddd, *J* = 13.5, 6.9, 3.8 Hz, 1H), 1.38 (tdd, *J* = 14.7, 7.9, 2.9 Hz, 1H), 1.22 (s, 6H), 1.22 (s, 6H), 0.97 (t, *J* = 7.5 Hz, 3H), 0.81 (t, *J* = 7.3 Hz, 3H), 0.07 (s, 9H) ppm.

^{13}**C NMR** (101 MHz, $CDCl_3$) δ 141.4, 115.1, 82.6, 48.8, 26.8, 25.4, 25.2, 22.2, 13.2, 12.7, 0.3 ppm.
Note: Carbon atom attached to boron is not visible due to quadrupolar relaxation.

^{29}**Si NMR** (80 MHz, $CDCl_3$) δ 3.9 ppm.

Minor diastereomer:
^{1}**H NMR** (400 MHz, $CDCl_3$) δ 5.90 (dt, *J* = 17.1, 10.0 Hz, 1H), 5.03 – 4.88 (m, 2H), 2.19 – 2.09 (m, 1H), 1.72 (dq, *J* = 14.9, 7.5 Hz, 1H), 1.64 – 1.45 (m, 2H), 1.45 – 1.31 (m, 1H), 1.22 (s, 6H), 1.20 (s, 6H), 0.92 (t, *J* = 7.5 Hz, 3H), 0.79 (t, *J* = 1.9 Hz, 3H), 0.02 (s, 9H) ppm.

^{13}**C NMR** (101 MHz, $CDCl_3$) δ 142.4, 115.1, 82.5, 48.4, 26.8, 25.6, 25.3, 22.3, 12.8, 12.1, 0.7 ppm.
Note: Carbon atom attached to boron is not visible due to quadrupolar relaxation.

^{29}**Si NMR** (80 MHz, $CDCl_3$) δ 4.2 ppm.

^{11}**B NMR** (128 MHz, $CDCl_3$): δ 34.4 ppm.

HRMS (APCI+) m/z: calcd. for $C_{17}H_{36}BO_2Si^+$ $[M+H]^+$: 311.2572, found: 311.2578.

Trimethyl((4*S,5*S**)-5-(4,4,5,5-tetramethyl-1,3,2-dioxaborolan-2-yl)-4-vinylnon-1-en-5-yl)silane (6f)**

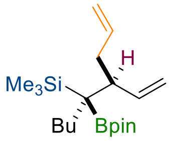

Chemical Formula: $C_{20}H_{39}BO_2Si$
Molecular Weight: 350.43

Prepared according to general procedure **GP1** from **5f** (101 mg, 250 μmol) and Me_3SiCl (159 μL, 1.25 mmol).

Yield: 60 mg (173 μmol, 69%) as a partially separable mixture of diastereomers (dr 75:25 as determined by ^{1}H NMR spectroscopy) as a colorless oil.

R*f* = 0.70 (PE/Et_2O 40:1).

Major diastereomer:
^{1}**H NMR** (400 MHz, $CDCl_3$) δ 5.84 – 5.65 (m, 2H), 5.04 – 4.84 (m, 4H), 2.49 – 2.25 (m, 2H), 2.21 – 2.09 (m, 1H), 1.66 – 1.55 (m, 1H), 1.54 – 1.44 (m, 2H), 1.40 – 1.29 (m, 3H), 1.22 (s, 12H), 0.88 (t, *J* = 7.1 Hz, 3H), 0.08 (s, 9H) ppm.

^{13}**C NMR** (101 MHz, $CDCl_3$) δ 141.0, 139.2, 115.2, 114.5, 82.6, 47.0, 38.6, 29.8, 29.6, 25.4, 25.2, 24.2, 14.4, 0.4 ppm.
Note: Carbon atom attached to boron is not visible due to quadrupolar relaxation.

^{29}**Si NMR** (80 MHz, $CDCl_3$) δ 4.1 ppm.

Minor diastereomer:
^{1}**H NMR** (400 MHz, $CDCl_3$) δ 5.96 (dt, *J* = 17.1, 10.0 Hz, 1H), 5.84 – 5.65 (m, 1H), 5.04 – 4.84 (m, 4H), 2.49 – 2.25 (m, 2H), 2.02 (td, *J* = 12.7, 7.0 Hz, 1H), 1.66 – 1.55 (m, 2H), 1.54 – 1.44 (m, 1H), 1.40 – 1.29 (m, 3H), 1.22 (s, 6H), 1.21 (s, 6H), 0.89 (t, *J* = 7.1 Hz, 3H), 0.08 (s, 9H) ppm.

^{13}**C NMR** (101 MHz, $CDCl_3$) δ 142.2, 139.1, 115.3, 114.7, 82.6, 46.9, 36.3, 29.7, 29.3, 25.5, 25.2, 24.1, 14.4, 0.7 ppm.
Note: Carbon atom attached to boron is not visible due to quadrupolar relaxation.

^{29}**Si NMR** (80 MHz, $CDCl_3$) δ 4.3 ppm.

^{11}**B NMR** (128 MHz, $CDCl_3$): δ 34.4 ppm.

HRMS (APCI+) m/z: calcd. for $C_{20}H_{40}BO_2Si^+$ $[M+H]^+$: 351.2885, found: 351.2903.

((1*S,2*S**)-1-Cyclohexyl-1-(4,4,5,5-tetramethyl-1,3,2-dioxaborolan-2-yl)-2-vinylpentyl)trimethylsilane (6g)**

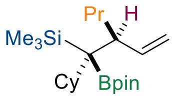

Chemical Formula: $C_{22}H_{43}BO_2Si$
Molecular Weight: 378.48

Prepared according to general procedure **GP1** from **5g** (121 mg, 281 μmol) and Me_3SiI (120 μL, 843 μmol).

Yield: 40 mg (106 μmol, 38%) as a partially separable mixture of diastereomers (dr 75:25 as determined by ^1H NMR spectroscopy) as a colorless oil.

R*f* = 0.45 (PE/Et_2O 40:1).

Major diastereomer:
^1H NMR (400 MHz, $CDCl_3$) δ 5.86 (dt, *J* = 17.0, 10.1 Hz, 1H), 4.96 – 4.80 (m, 2H), 2.44 (t, *J* = 10.5 Hz, 1H), 1.85 – 1.64 (m, 4H), 1.63 – 1.46 (m, 6H), 1.39 – 1.07 (m, 5H), 1.22 (s, 6H), 1.21 (s, 6H), 0.86 (t, *J* = 7.2 Hz, 3H), 0.13 (s, 9H) ppm.

^{13}C NMR (101 MHz, $CDCl_3$) δ 144.3, 114.5, 82.5, 46.7, 42.5, 36.3, 32.8, 31.9, 28.2, 28.0, 26.7, 25.7, 25.3, 21.3, 14.3, 2.7 ppm.
Note: Carbon atom attached to boron is not visible due to quadrupolar relaxation.

^{29}Si NMR (80 MHz, $CDCl_3$) δ 3.9 ppm.

Minor diastereomer:
^1H NMR (400 MHz, $CDCl_3$) δ 5.96 (dt, *J* = 17.3, 9.9 Hz, 1H), 4.96 – 4.80 (m, 2H), 2.28 (t, *J* = 10.1 Hz, 1H), 1.85 – 1.64 (m, 4H), 1.63 – 1.46 (m, 6H), 1.39 – 1.07 (m, 5H), 1.24 (s, 6H), 1.22 (s, 6H), 0.85 (t, *J* = 7.2 Hz, 3H), 0.12 (s, 9H) ppm.

^{13}C NMR (101 MHz, $CDCl_3$) δ 145.1, 113.6, 82.5, 46.3, 42.7, 36.4, 33.4, 30.9, 28.3, 27.8, 26.7, 25.8, 25.6, 21.6, 14.2, 2.0 ppm.
Note: Carbon atom attached to boron is not visible due to quadrupolar relaxation.

^{29}Si NMR (80 MHz, $CDCl_3$) δ 5.5 ppm.

^{11}B NMR (128 MHz, $CDCl_3$): δ 33.6 ppm.

HRMS (APCI+) m/z: calcd. for $C_{22}H_{44}BO_2Si^+$ [*M*+H]$^+$: 379.3198, found: 378.3199.

Trimethyl((2*S*,3*S*)-3-methyl-2-(4,4,5,5-tetramethyl-1,3,2-dioxaborolan-2-yl)-3-vinylhex-5-en-2-yl)silane (6h)

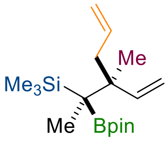

Chemical Formula: $C_{18}H_{35}BO_2Si$
Molecular Weight: 322.37

Prepared according to general procedure **GP1** from **5h** (41mg, 110 μmol) and Me_3SiCl (70 μL, 550 μmol).

Yield: 12 mg (37 μmol, 34%) as an inseparable mixture of diastereomers (dr 90:10 as determined by ^1H NMR spectroscopy) as a colorless oil.

R*f* = 0.35 (PE/Et_2O 40:1).

Major diastereomer:
^1H NMR (400 MHz, $CDCl_3$) δ 5.84 (dd, *J* = 17.5, 10.8 Hz, 1H), 5.74 – 5.58 (m, 1H), 5.06 – 4.88 (m, 3H), 4.80 (dd, *J* = 17.5, 1.5 Hz, 1H), 2.68 (dd, *J* = 13.6, 5.7 Hz, 1H), 2.12 (dd, *J* = 13.6, 8.5 Hz, 1H), 1.23 (s, 12H), 1.03 (s, 3H), 1.02 (s, 3H), 0.09 (s, 9H) ppm.

^{13}C NMR (101 MHz, $CDCl_3$) δ 145.8, 137.1, 116.0, 112.2, 82.9, 44.3, 29.9, 25.6, 25.2, 20.5, 14.2, 1.1 ppm. *Note*: Carbon atom attached to boron is not visible due to quadrupolar relaxation.

^{29}Si NMR (80 MHz, $CDCl_3$) δ 3.7 ppm.

Minor diastereomer:
^1H NMR (400 MHz, $CDCl_3$) δ 6.06 (dd, *J* = 16.6, 10.6 Hz, 1H), 5.74 – 5.58 (m, 1H), 5.24 – 5.07 (m, 1H), 5.06 – 4.88 (m, 2H), 4.82 (dd, *J* = 17.6, 1.5 Hz, 1H), 2.37 (dd, *J* = 13.4, 8.7 Hz, 1H), 2.12 (dd, *J* = 13.6, 8.5 Hz, 1H), 1.25 (s, 12H), 1.02 (s, 3H), 1.01 (s, 3H), 0.08 (s, 9H) ppm.

^{13}C NMR (101 MHz, $CDCl_3$) δ 146.6, 136.9, 116.0, 111.9, 82.9, 44.5, 29.9, 25.6, 25.2, 20.2, 14.3, 1.0 ppm. *Note*: Carbon atom attached to boron is not visible due to quadrupolar relaxation.

^{29}Si NMR (80 MHz, $CDCl_3$) δ 3.3 ppm.

^{11}B NMR (128 MHz, $CDCl_3$): δ 34.3 ppm.

HRMS (APCI+) m/z: calcd. for $C_{18}H_{36}BO_2Si^+$ $[M+H]^+$: 323.2572, found: 323.2572.

Tributyl((3*R,4*R**)-4-ethyl-3-(4,4,5,5-tetramethyl-1,3,2-dioxaborolan-2-yl)hex-5-en-3-yl)stannane (6i)**

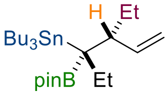

Chemical Formula: $C_{26}H_{53}BO_2Sn$
Molecular Weight: 527.23

Prepared according to general procedure **GP1** from **5e** (73 mg, 200 μmol) and *n*-Bu_3SnCl (163 μL, 600 μmol).

Yield: 60 mg (114 μmol, 57%) as a partially separable mixture of diastereomers (dr 75:25 as determined by ^1H NMR spectroscopy) as a colorless oil.

R*f* = 0.65 (PE/Et_2O 40:1).

Major diastereomer:
^{1}**H NMR** (400 MHz, $CDCl_3$) δ 5.58 (dt, *J* = 17.1, 9.9 Hz, 1H), 5.04 – 4.82 (m, 2H), 2.25 – 2.14 (m, 1H), 1.90 – 1.71 (m, 2H), 1.67 – 1.58 (m, 1H), 1.56 – 1.38 (m, 6H), 1.31 (ddt, *J* = 10.4, 7.3, 3.3 Hz, 6H), 1.24 – 1.15 (m, 1H), 1.21 (s, 6H), 1.19 (s, 6H), 0.94 – 0.83 (m, 18H), 0.82 (t, *J* = 7.2 Hz, 3H) ppm.

^{13}**C NMR** (101 MHz, $CDCl_3$) δ 142.1, 114.8, 82.3, 49.4, 29.48, 28.2, 27.8, 25.6, 25.0, 23.7, 13.8, 13.3, 13.0, 10.9 ppm.
Note: Carbon atom attached to boron is not visible due to quadrupolar relaxation.

Minor diastereomer:
^{1}**H NMR** (400 MHz, $CDCl_3$) δ 5.90 (dt, *J* = 17.1, 10.0 Hz, 1H), 5.02 – 4.92 (m, 2H), 2.25 – 2.14 (m, 1H), 1.90 – 1.71 (m, 2H), 1.67 – 1.58 (m, 1H), 1.56 – 1.38 (m, 6H), 1.31 (ddt, *J* = 10.4, 7.3, 3.3 Hz, 6H), 1.24 – 1.15 (m, 1H), 1.21 (s, 6H), 1.19 (s, 6H), 0.94 – 0.83 (m, 18H), 0.80 (t, *J* = 7.2 Hz, 3H) ppm.

^{13}**C NMR** (101 MHz, $CDCl_3$) δ 143.2, 114.6, 82.3, 49.1, 29.51, 28.2, 27.9, 25.6, 25.0, 23.7, 14.0, 13.6, 13.0, 11.1 ppm.
Note: Carbon atom attached to boron is not visible due to quadrupolar relaxation.

^{11}**B NMR** (128 MHz, $CDCl_3$): δ 33.6 ppm.

(4-Ethylhex-5-en-3-ylidene)cyclohexane (6j)

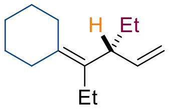

Chemical Formula: $C_{14}H_{24}$
Molecular Weight: 192.35

Prepared according to general procedure **GP1** from **5e** (55 mg, 150 µmol) and cyclohexanone (79 µL, 750 µmol). The reaction mixture was and stirred overnight at rt before being quenched by MeOH.

Yield: 16 mg (84 µmol, 56%) as a colorless oil.

R*f* = 0.75 (PE).

^{1}**H NMR** (400 MHz, $CDCl_3$) δ 5.82 (ddd, *J* = 17.2, 10.6, 6.8 Hz, 1H), 4.98 (dt, *J* = 8.3, 1.4 Hz, 1H), 4.94 (d, *J* = 1.3 Hz, 1H), 3.15 (q, *J* = 6.8 Hz, 1H), 2.16 (dddd, *J* = 24.7, 20.5, 13.0, 4.9 Hz, 4H), 1.96 (q, *J* = 7.5 Hz, 2H), 1.64 – 1.46 (m, 4H), 1.42 (dq, *J* = 20.9, 7.5 Hz, 4H), 0.93 (t, *J* = 7.5 Hz, 3H), 0.84 (t, *J* = 7.4 Hz, 3H) ppm.

^{13}**C NMR** (101 MHz, $CDCl_3$) δ 142.4, 135.0, 132.1, 113.4, 47.2, 31.3, 30.6, 28.8, 28.7, 27.3, 25.5, 21.4, 15.7, 12.5 ppm.

HRMS (APCI+) m/z: calcd. for $C_{14}H_{23}$ $[M–H]^+$: 191.1794, found: 191.1794.

2-((1*R,2*S**)-1-Butyl-2-vinylcyclopentyl)-4,4,5,5-tetramethyl-1,3,2-dioxaborolane (6k)**

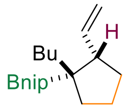

Chemical Formula: $C_{17}H_{31}BO_2$
Molecular Weight: 278.24

Prepared according to general procedure **GP1** from **5i** (66 mg, 150 μmol).

Yield: 35 mg (126 μmol, 84%, dr >95:05 as determined by ^{1}H NMR spectroscopy) as a colorless oil.

R*f* = 0.50 (PE/Et_2O 40:1).

^{1}H NMR (400 MHz, $CDCl_3$) δ 5.89 (ddd, *J* = 17.1, 10.0, 8.8 Hz, 1H), 5.05 – 4.94 (m, 1H), 4.90 (dd, *J* = 10.1, 2.1 Hz, 1H), 2.07 (q, *J* = 8.8 Hz, 1H), 1.95 (ddd, *J* = 12.8, 8.5, 4.8 Hz, 1H), 1.79 (td, *J* = 8.4, 4.7 Hz, 2H), 1.62 (dddd, *J* = 19.4, 16.4, 9.4, 4.7 Hz, 2H), 1.52 – 1.43 (m, 1H), 1.31 – 1.25 (m, 6H), 1.22 (s, 6H), 1.21 (s, 6H), 0.87 (t, *J* = 7.0 Hz, 3H) ppm.

^{13}C NMR (101 MHz, $CDCl_3$) δ 142.0, 113.7, 83.0, 55.8, 38.2, 34.4, 32.0, 29.9, 25.2, 24.9, 23.8, 23.0, 14.3 ppm.
Note: Carbon atom attached to boron is not visible due to quadrupolar relaxation.

^{11}B NMR (128 MHz, $CDCl_3$): δ 34.6 ppm.

HRMS (APCI+) m/z: calcd. for $C_{17}H_{32}BO_2^+$ [*M*+H]$^+$: 279.2490, found: 279.2498.

4. Determination of the stereochemical outcome

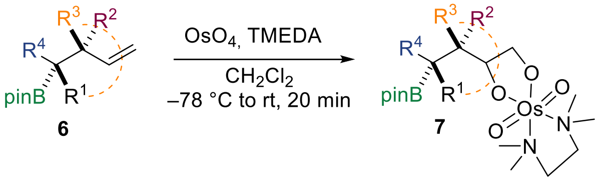

Dioxo[*N*,*N*,*N*,*N*-tetramethylethane-1,2-diaminetetramethylehylendiamine][(2*R,3*R**,4*R**)-4-(dimethyl(phenyl)silyl)-3-methyl-4-(4,4,5,5-tetramethyl-1,3,2-dioxaborolan-2-yl)pentane-1,2-diol]osmium complex (7b)**

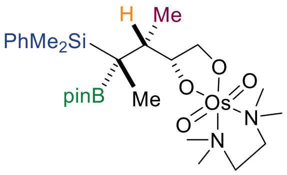

Chemical Formula: $C_{26}H_{49}BN_2O_6OsSi$
Molecular Weight: 714.81

Prepared by according to a literature procedure[5] from **6b** (36 mg, 105 μmol).

Yield: 75 mg (105 μmol, quantitative; dr >95:05 as determined by ^{1}H NMR spectroscopy) as a dark red solid.

R*f* = 0.30 (DCM/MeOH 9:1).

^{1}H NMR (400 MHz, $CDCl_3$) δ 7.64 (dd, *J* = 6.4, 2.9 Hz, 2H), 7.27 (dd, *J* = 5.3, 2.7 Hz, 3H), 4.53 (ddd, *J* = 10.7, 6.2, 4.5 Hz, 1H), 4.14 (dd, *J* = 9.7, 4.4 Hz, 1H), 4.05 (t, *J* = 10.2 Hz, 1H), 3.02 (ddd, *J* = 24.1, 19.7, 12.1 Hz, 4H), 2.82 (s, 6H), 2.81 (s, 3H), 2.79 (s, 3H), 2.51 (p, *J* = 7.0 Hz, 1H), 1.23 (s, 3H), 1.12 (s, 6H), 1.08 (d, *J* = 7.1 Hz, 3H), 0.98 (s, 6H), 0.53 (s, 3H), 0.37 (s, 3H) ppm.

^{13}C NMR (101 MHz, $CDCl_3$) δ 140.6, 135.2, 128.4, 127.3, 91.6, 83.3, 82.8, 64.6, 64.1, 52.2, 51.9, 51.4, 51.1, 40.1, 25.5, 25.2, 16.4, 13.8, -1.7, -2.4 ppm.

^{11}B NMR (128 MHz, $CDCl_3$): δ 36.2 ppm.

^{29}Si NMR (80 MHz, $CDCl_3$) δ -0.9 ppm.

HRMS (APCI+) m/z: calcd. for $C_{26}H_{50}BN_2O_6OsSi^+$ [*M*+H]$^+$: 717.3141, found: 717.3126.

Recrystallization by vapour diffusion (THF with pentane as antisolvent) yielded X-ray quality crystals.

Dioxo[*N*,*N*,*N*,*N*-tetramethylethane-1,2-diaminetetramethylehylendiamine][(1-((1*R,2*R**)-2-butyl-2-(4,4,5,5-tetramethyl-1,3,2-dioxaborolan-2-yl)cyclopentyl)ethane-1,2-diol]osmium complex (7k)**

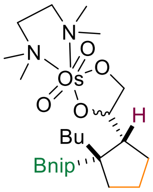

Chemical Formula: $C_{23}H_{47}BN_2O_6Os$
Molecular Weight: 648.68

Prepared by according to a literature procedure[5] from **6k** (28 mg, 90 µmol).

Yield: 58 mg (89 µmol, quantitative) as a partially separable mixture of diastereomers (dr 75:25 as determined by ^{1}H NMR spectroscopy) as a dark red solid.

R*f* = 0.35 (DCM/MeOH 9:1).

Major diastereomer:
^{1}H NMR (400 MHz, $CDCl_3$) δ 4.50 – 4.43 (m, 1H), 4.37 (dd, *J* = 9.8, 4.7 Hz, 1H), 4.13 – 4.05 (m, 1H), 3.14 – 2.97 (m, 4H), 2.87 – 2.81 (m, 12H), 2.17 (q, *J* = 7.2 Hz, 1H), 1.99 – 1.77 (m, 3H), 1.73 – 1.55 (m, 4H), 1.55 – 1.45 (m, 1H), 1.45 – 1.37 (m, 1H), 1.32 – 1.15 (m, 3H), 1.22 (s, 6H), 1.20 (s, 6H), 0.85 (t, *J* = 6.8 Hz, 3H) ppm.

^{13}C NMR (101 MHz, $CDCl_3$) δ 91.7, 85.1, 82.9, 64.4, 64.2, 52.2, 52.0, 51.3, 51.3, 51.1, 38.7, 34.4, 29.5, 28.6, 25.2, 25.1, 23.9, 23.8, 14.3 ppm.
Note: Carbon atom attached to boron is not visible due to quadrupolar relaxation.

Minor diastereomer:
^{1}H NMR (400 MHz, $CDCl_3$) δ 4.45 – 4.40 (m, 1H), 4.25 (dd, *J* = 9.7, 4.4 Hz, 1H), 3.97 (t, *J* = 9.1 Hz, 1H), 3.14 – 2.97 (m, 4H), 2.87 – 2.81 (m, 12H), 2.17 (q, *J* = 7.2 Hz, 1H), 1.99 – 1.77 (m, 3H), 1.73 – 1.55 (m, 4H), 1.55 – 1.45 (m, 1H), 1.45 – 1.37 (m, 1H), 1.32 – 1.15 (m, 3H), 1.18 (s, 12H), 0.84 (t, *J* = 7.5 Hz, 3H) ppm.

^{13}C NMR (101 MHz, $CDCl_3$) δ 93.3, 85.9, 82.4, 64.5, 64.2, 52.5, 52.4, 51.6, 51.0, 50.9, 39.4, 35.5, 31.4, 29.0, 25.3, 24.8, 24.7, 24.1, 14.4 ppm.
Note: Carbon atom attached to boron is not visible due to quadrupolar relaxation.

^{11}B NMR (128 MHz, $CDCl_3$): δ 36.5 ppm.

HRMS (APCI+) m/z: calcd. for $C_{23}H_{48}BN_2O_6Os^+$ [*M*+H]$^+$: 651.3215, found: 651.3229.

Recrystallization by vapour diffusion (DCM/Et_2O 1:3 with pentane as antisolvent) yielded X-ray quality crystals.

Crystal structures of 7b and 7k
The single crystal of light brown plate (**7b**, Marek15R) from THF/pentane and the single crystal of light brown plate (**7k**, Marek44R) from Et_2O/DCM/pentane, in Paratone–N oil and mounted on a Rigaku Oxford Diffraction - XtaLAB Synergy-S at 100 K. Data collection was performed using monochromated Mo Kα radiation, λ = 0.71073 Å. Accurate cell parameters were obtained with the amount of indicated reflections. Using Olex2[6], the structure was solved with the olex2.solve[7] structure solution program using Charge Flipping and refined with the ShelXL[8] refinement package using Least Squares minimisation. All non-hydrogen atoms were refined with anisotropic displacement parameters. The hydrogen atoms were refined isotropically in calculated positions using a riding model with their U_{iso} values constrained to 1.5 times the

U_{eq} of their pivot atoms for terminal sp^3 carbon atoms and 1.2 times for all other carbon atoms. Software used for molecular graphics: Mercury 2022.3.0.[9]

Crystal structure determination of 7b and 7k

Crystal data	Marek15R (7b)	Marek44R (7k)
CCDC code	**2537818**	**2537817**
Empirical formula	$C_{26}H_{49}BClN_2O_6OsSi$	$C_{46}H_{94}B_2N_4O_{12}Os_2$
Formula weight	750.22	1297.34
Temperature (K)	100.15	100.15
Wavelength (Å)	0.71073	0.71073
Crystal system,	triclinic	triclinic
space group	P-1	P-1
a (Å)	9.35848(18)	9.9793(6)
b (Å)	12.2804(3)	12.4650(6)
c (Å)	29.0612(6)	21.9750(13)
alpha	84.4348(18)	87.307(4)
beta	85.4484(17)	84.945(5)
gamma	89.9944(17)	89.968(4)
Volume (Å^3)	3313.57(12)	2719.9(3)
Z	4	2
Calculated density (mg/m^3)	1.504	1.584
Absorption coefficient (mm^{-1})	4.003	4.726
F(000)	1516	1312
Crystal size (mm)	0.24 × 0.18 × 0.15	0.18 × 0.18 × 0.09
2Theta range	4.574 - 59.82	4.648 - 60.3
Reflection collected/unique	47960 / 15023	34192 / 12525
Rint	0.0605	0.0832
Completeness (%)	98.9	99.8
Absorption correction	semi-empirical	semi-empirical
Data/restraints/ parameters	15023 / 1164 / 814	12525 / 626 / 614
Goodness-of-fit on F^2	1.030	1.033
R1, wR2 [I>2sigma(I)]	0.0615, 0.1629	0.0668, 0.1658
R1, wR2 (all data)	0.0765, 0.1728	0.0928, 0.1804
Largest diff. peak and hole	3.73/-3.15	3.04/-2.40
Diffractometer	XtaLAB Synergy-S	XtaLAB Synergy-S

5. NMR spectra

((1*S,2*R**,3*S**)-3-Allyl-2-methyl-2-(4,4,5,5-tetramethyl-1,3,2-dioxaborolan-2-yl)cyclopropyl)methanol (5b-OH)**

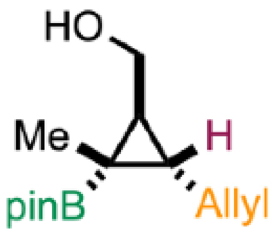

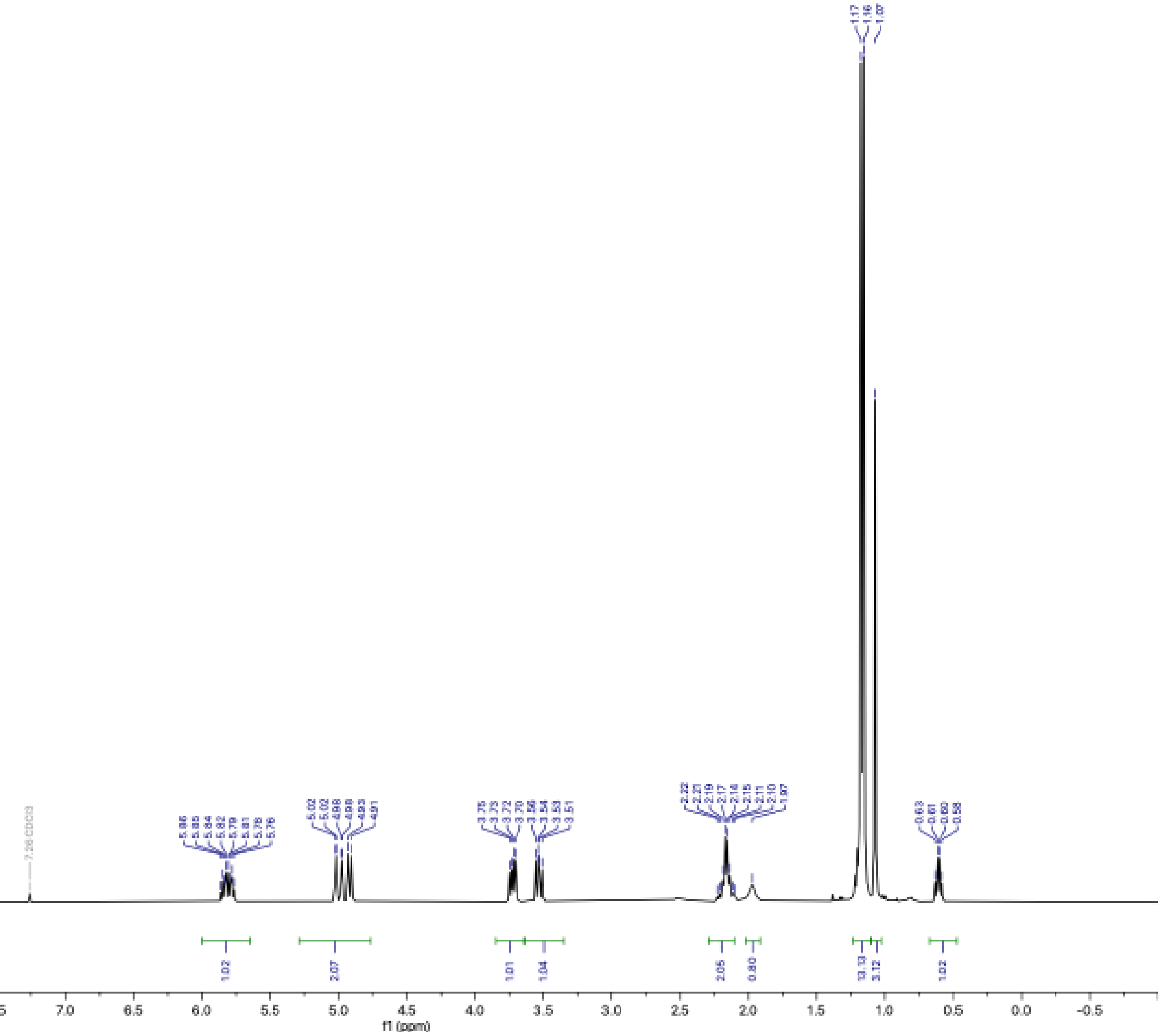

^{1}H NMR spectrum (400 MHz, $CDCl_3$)

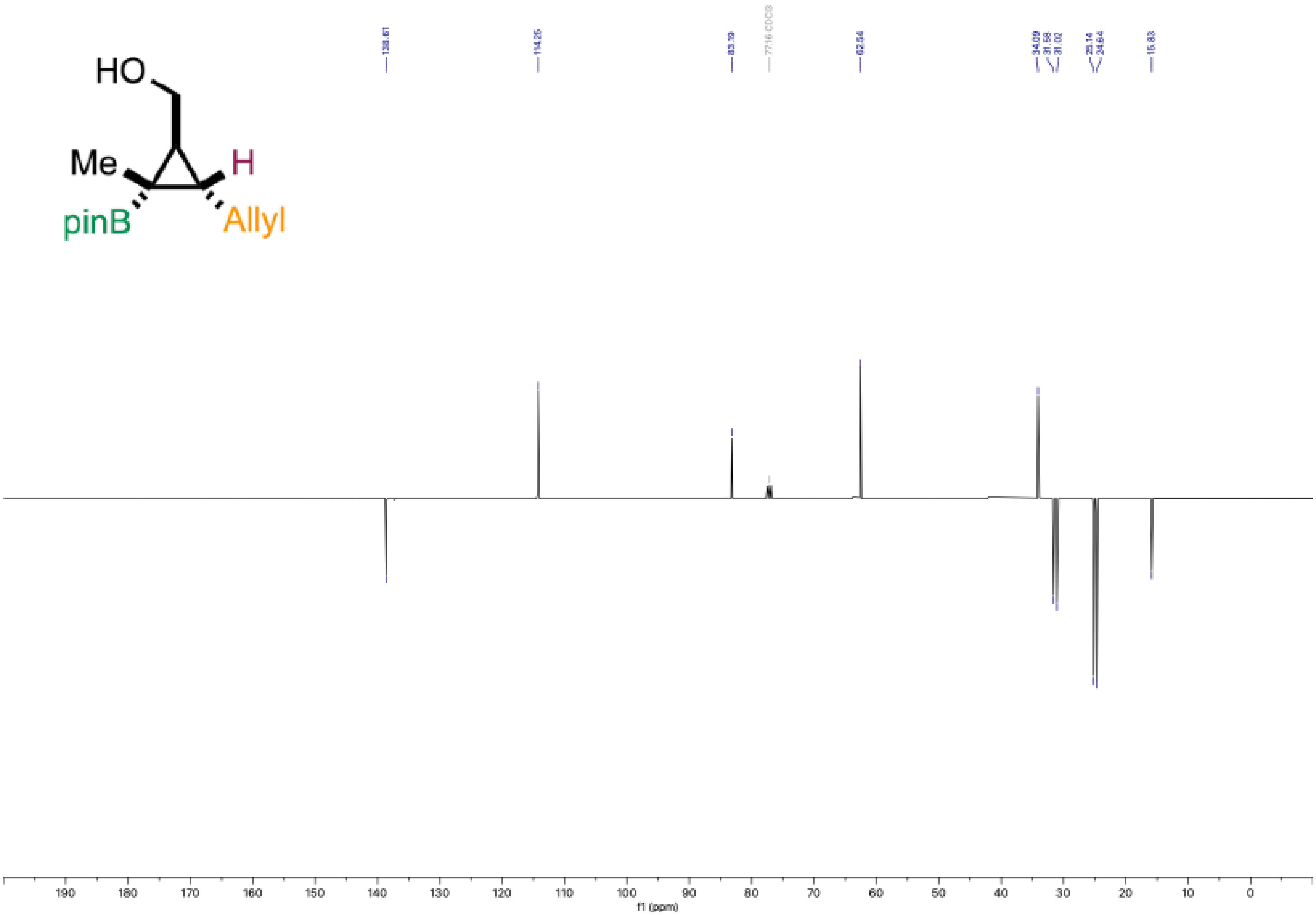

^{13}**C NMR** spectrum (101 MHz, $CDCl_3$)

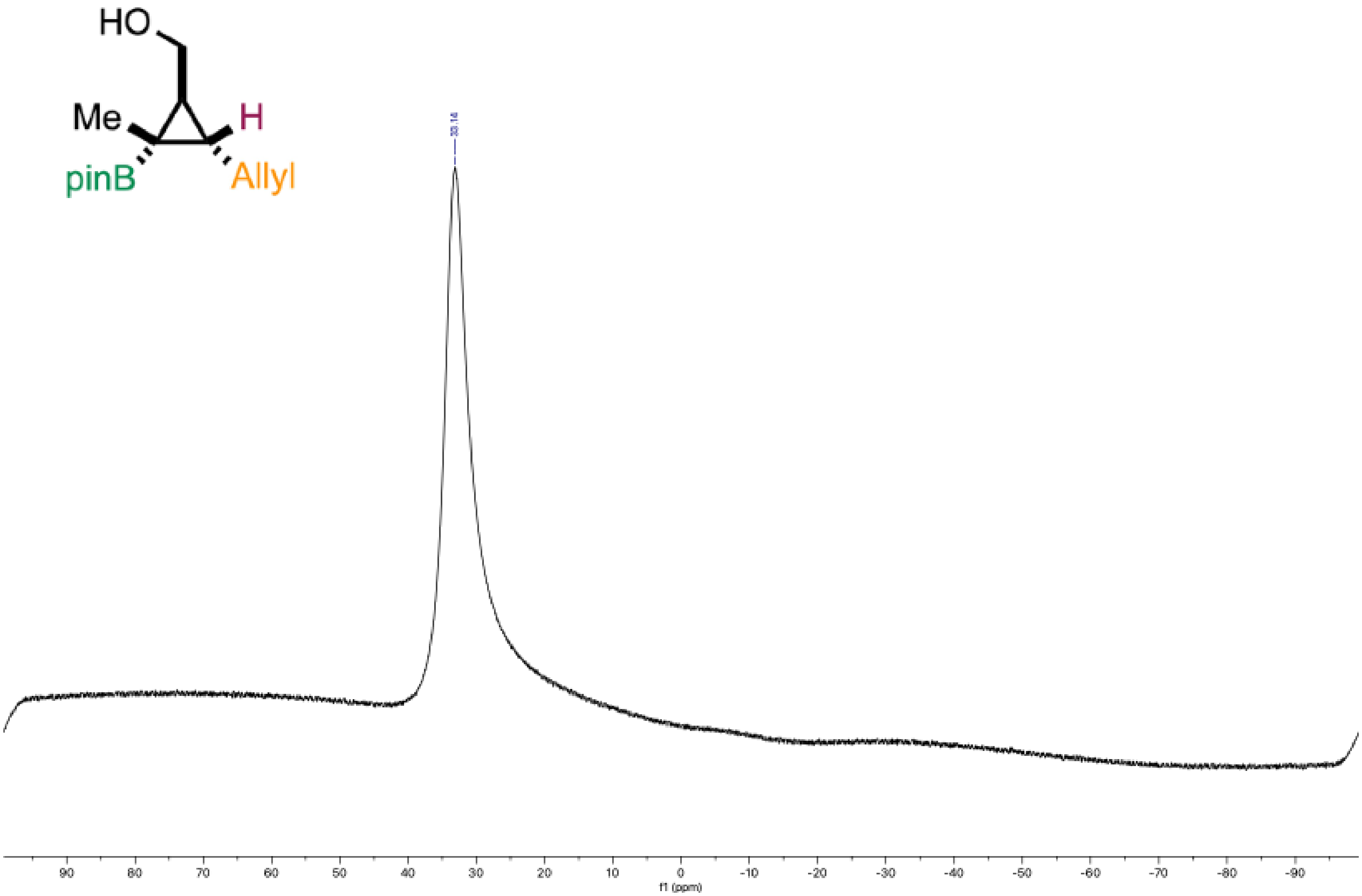

[11]**B NMR** spectrum (128 MHz, $CDCl_3$)

((1*S,2*R**,3*R**)-2,3-dipropyl-2-(4,4,5,5-tetramethyl-1,3,2-dioxaborolan-2-yl)cyclopropyl)methanol**
5c-OH

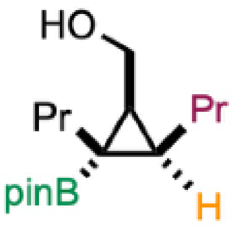

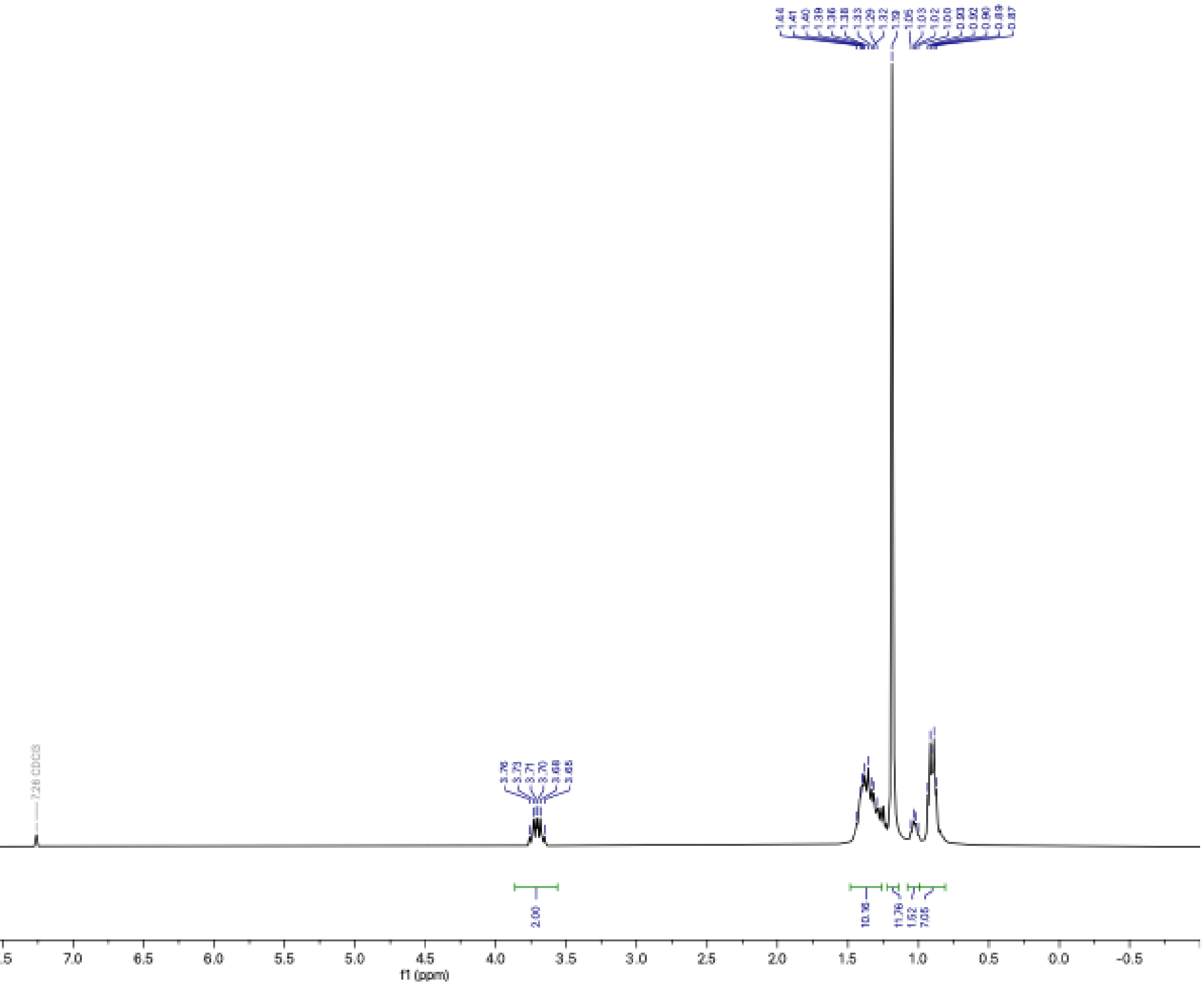

^{1}H NMR spectrum (400 MHz, $CDCl_3$)

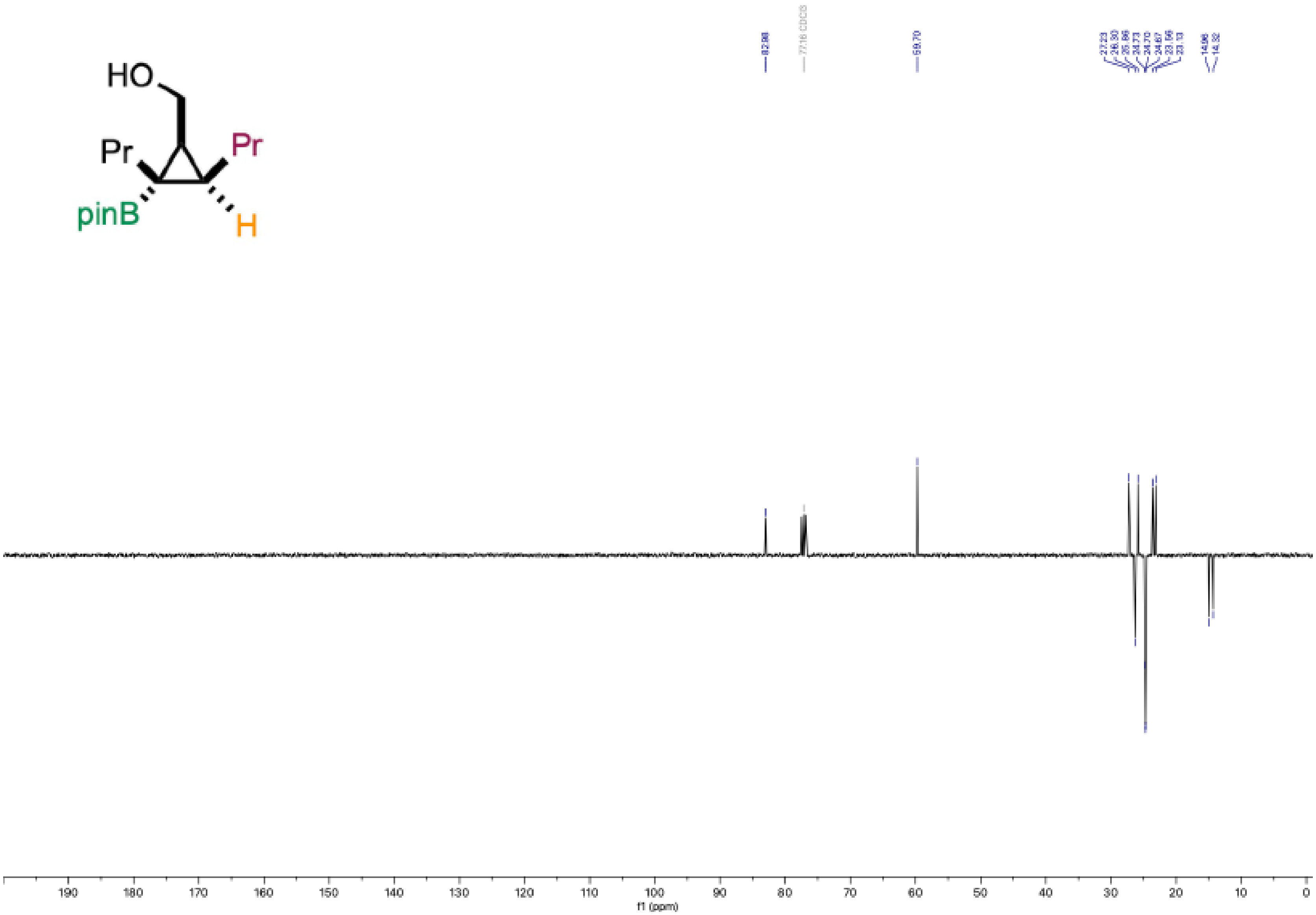

^{13}C NMR spectrum (101 MHz, $CDCl_3$)

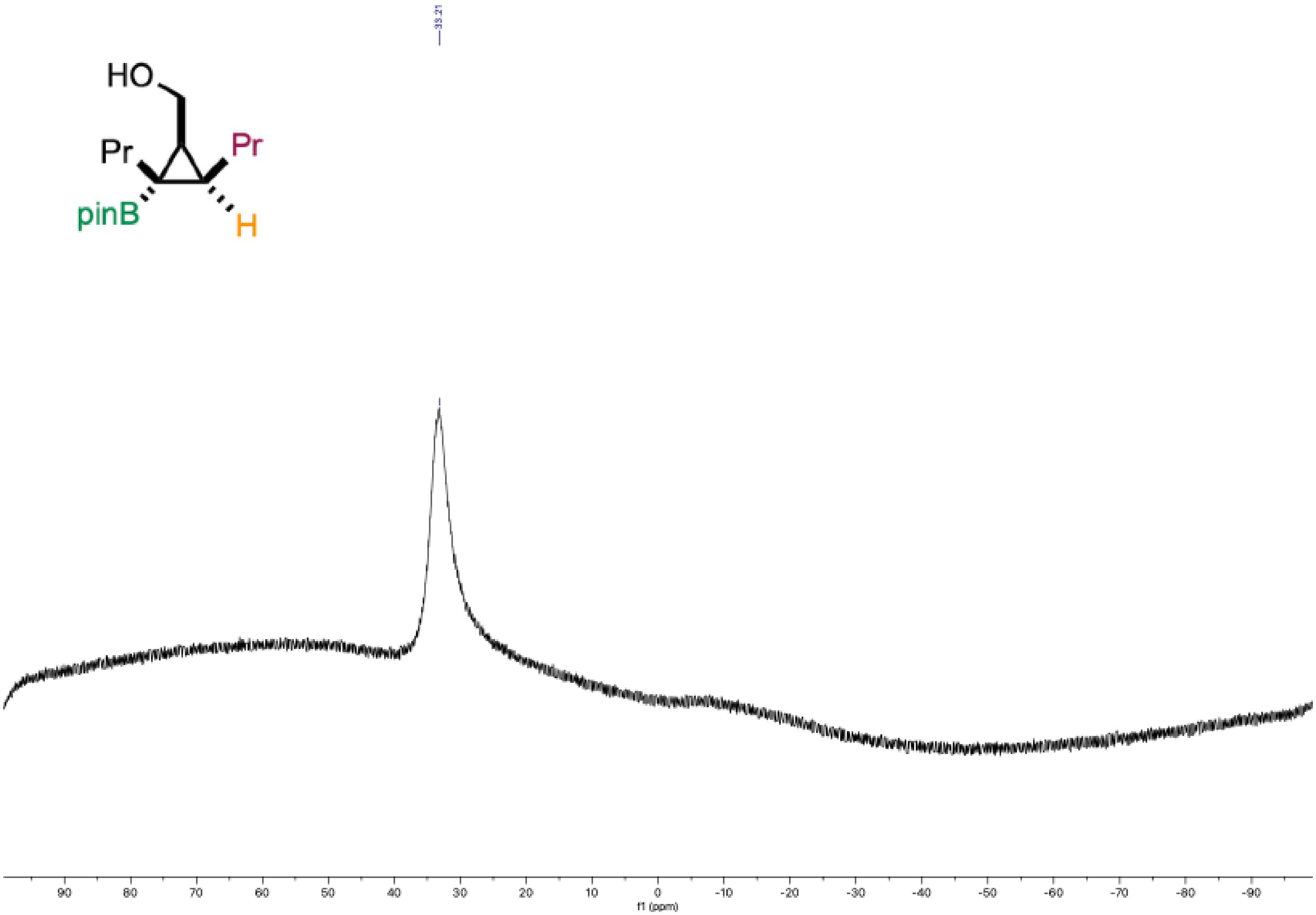

^{11}B NMR spectrum (128 MHz, $CDCl_3$)

((1*S,2*R**,3*S**)-2,3-Dipropyl-2-(4,4,5,5-tetramethyl-1,3,2-dioxaborolan-2-yl)cyclopropyl)methanol (5d-OH)**

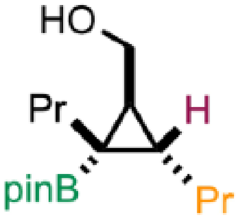

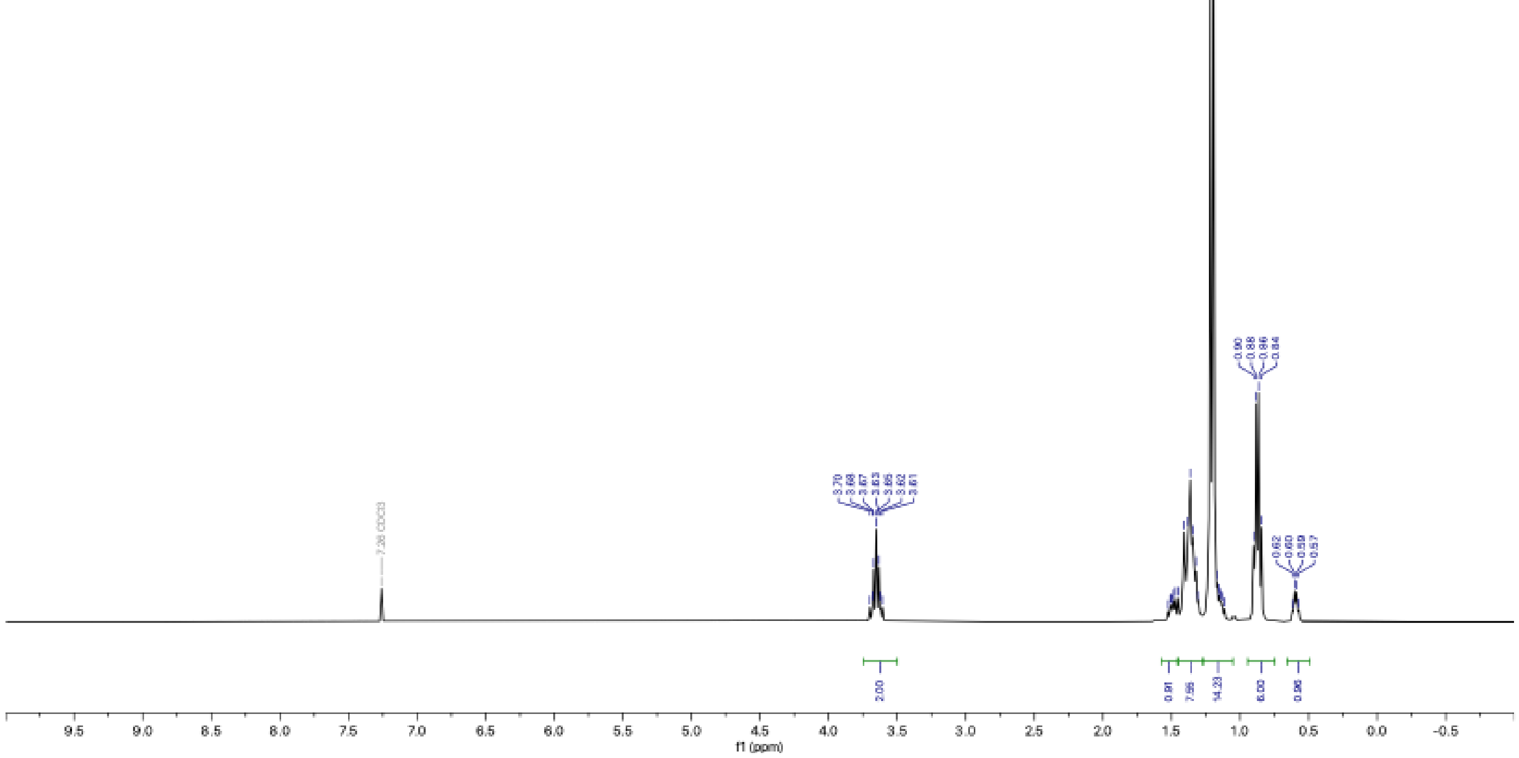

^{1}H NMR spectrum (400 MHz, $CDCl_3$)

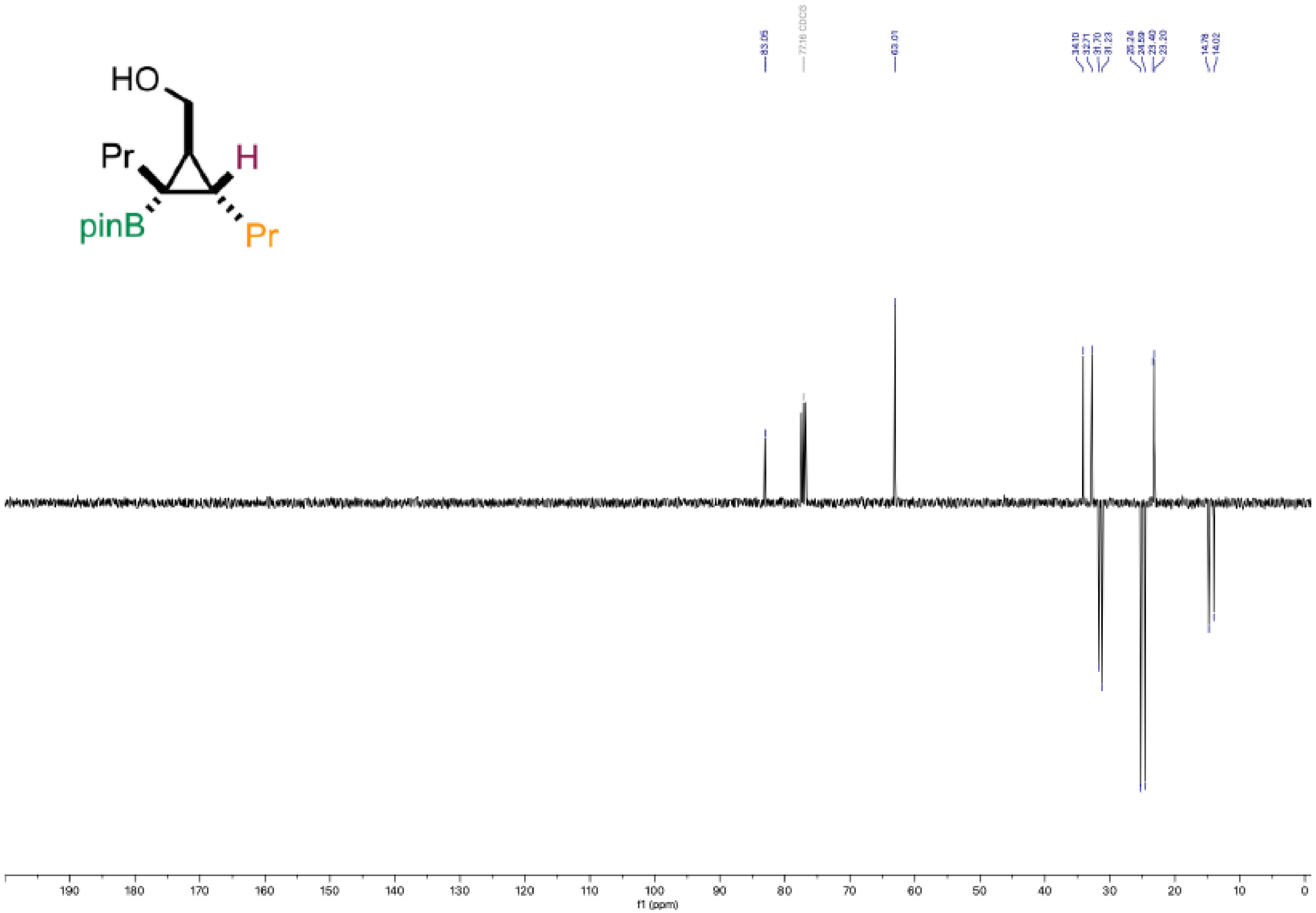

^{13}C NMR spectrum (101 MHz, $CDCl_3$)

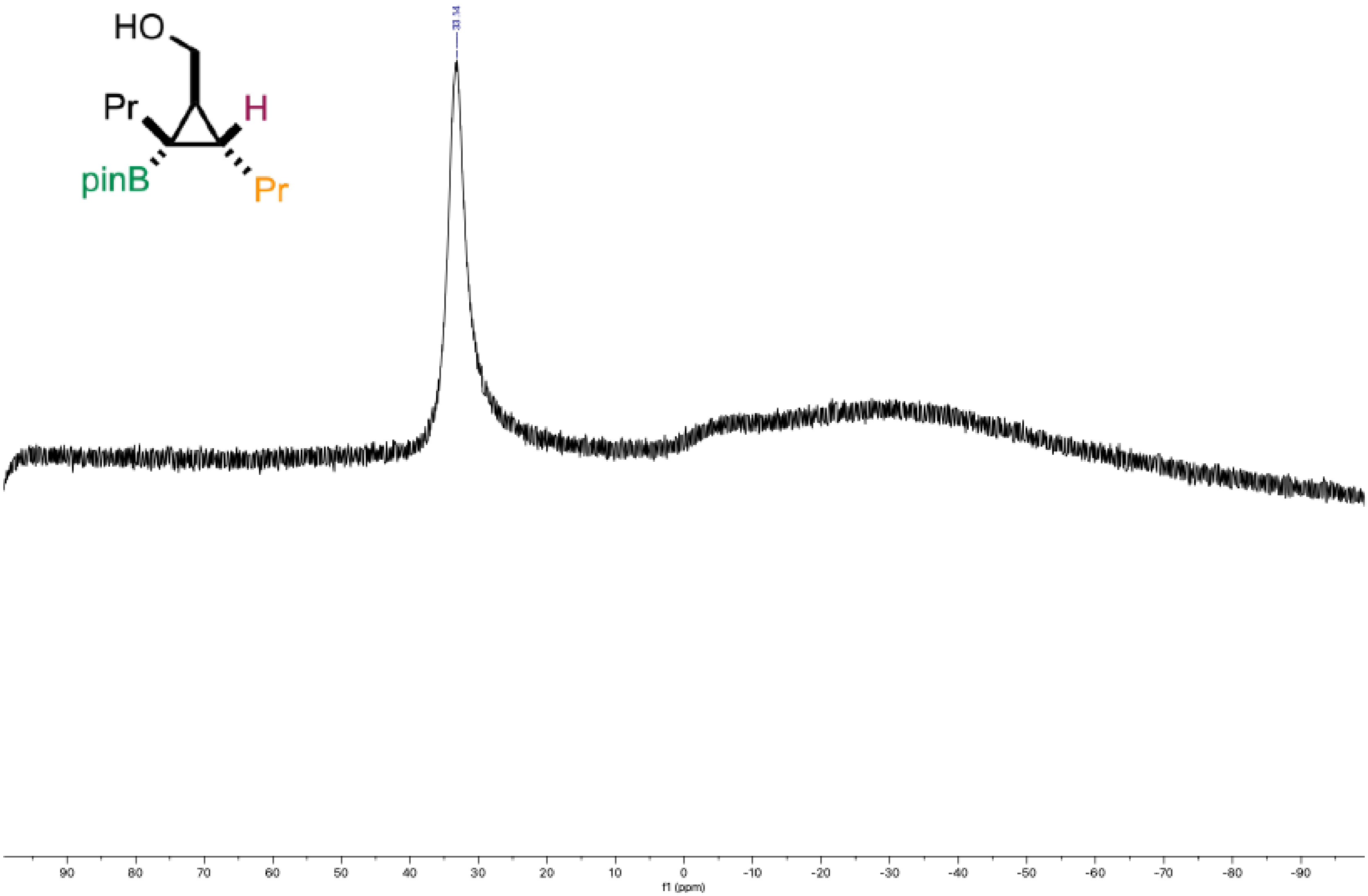

^{11}B NMR spectrum (128 MHz, $CDCl_3$)

((1*S,2*R**,3*S**)-2-Cyclohexyl-3-propyl-2- (4,4,5,5-tetramethyl-1,3,2-dioxaborolan-2-yl)cyclopropyl)methanol (5g-OH)**

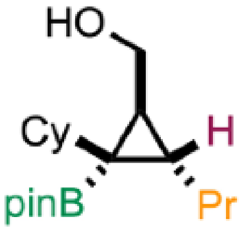

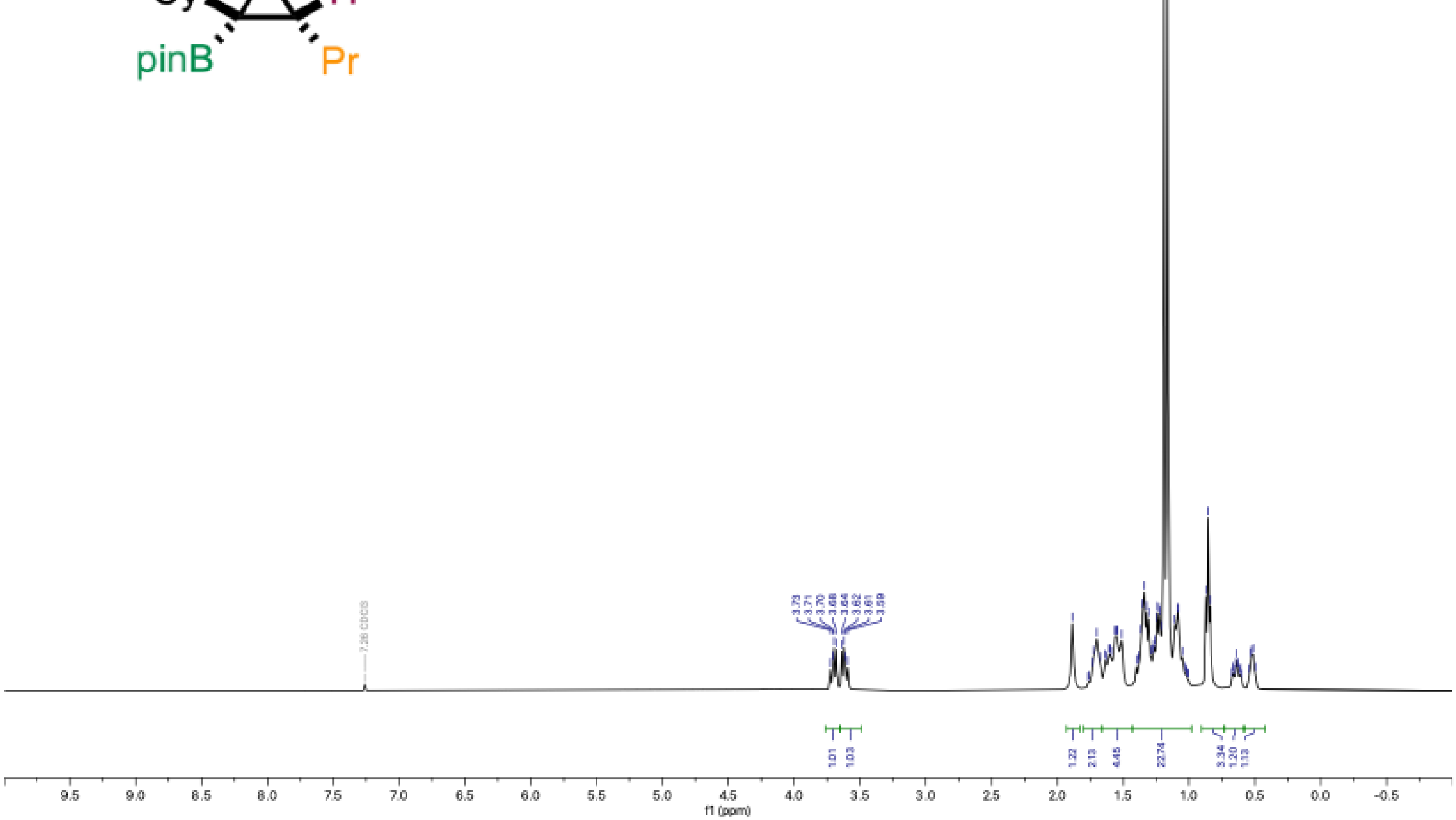

^{1}H NMR spectrum (400 MHz, $CDCl_3$)

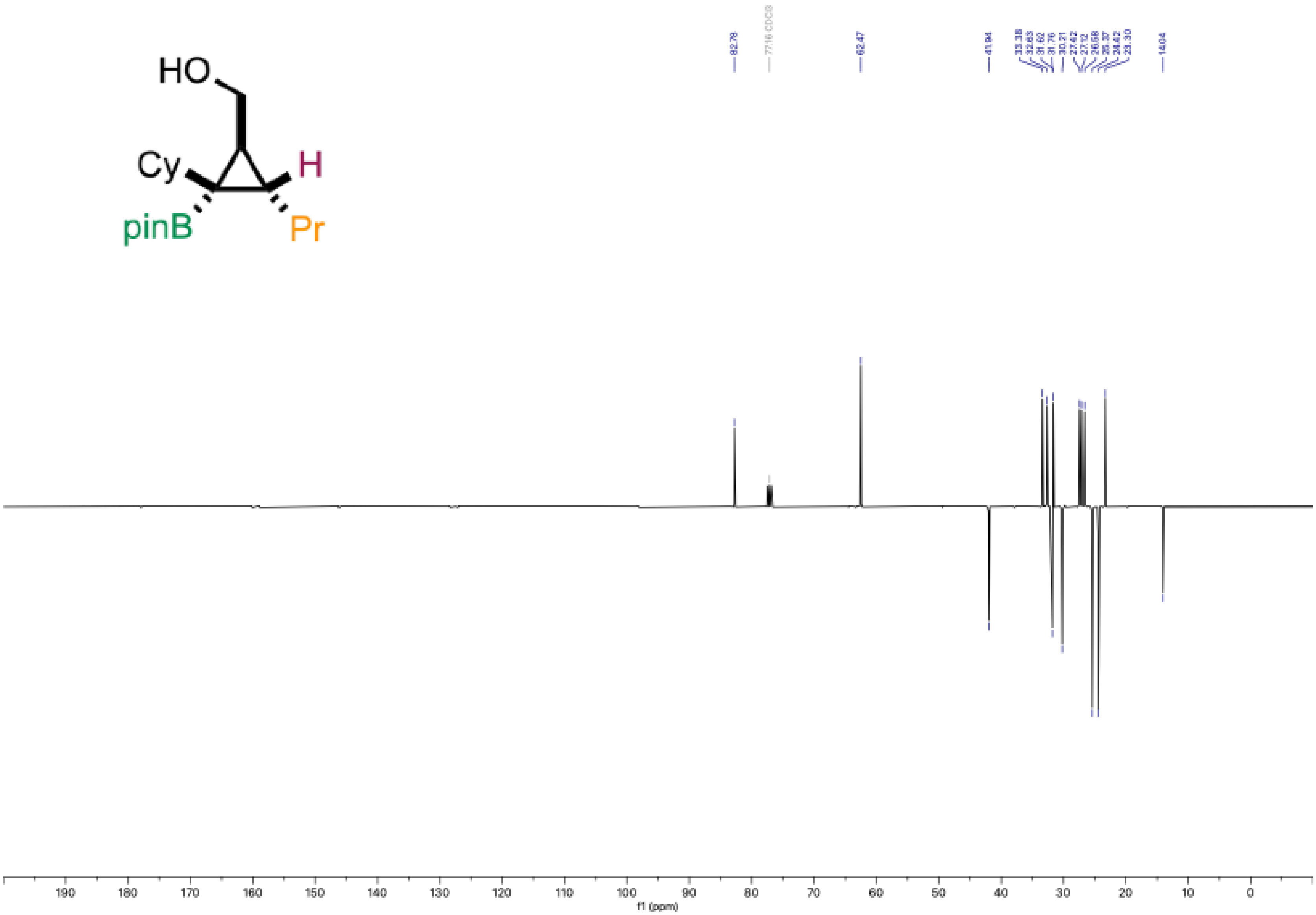

[13]**C NMR** spectrum (101 MHz, $CDCl_3$)

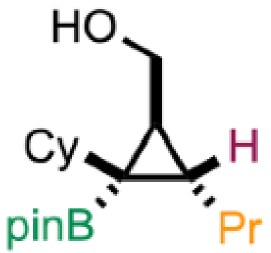

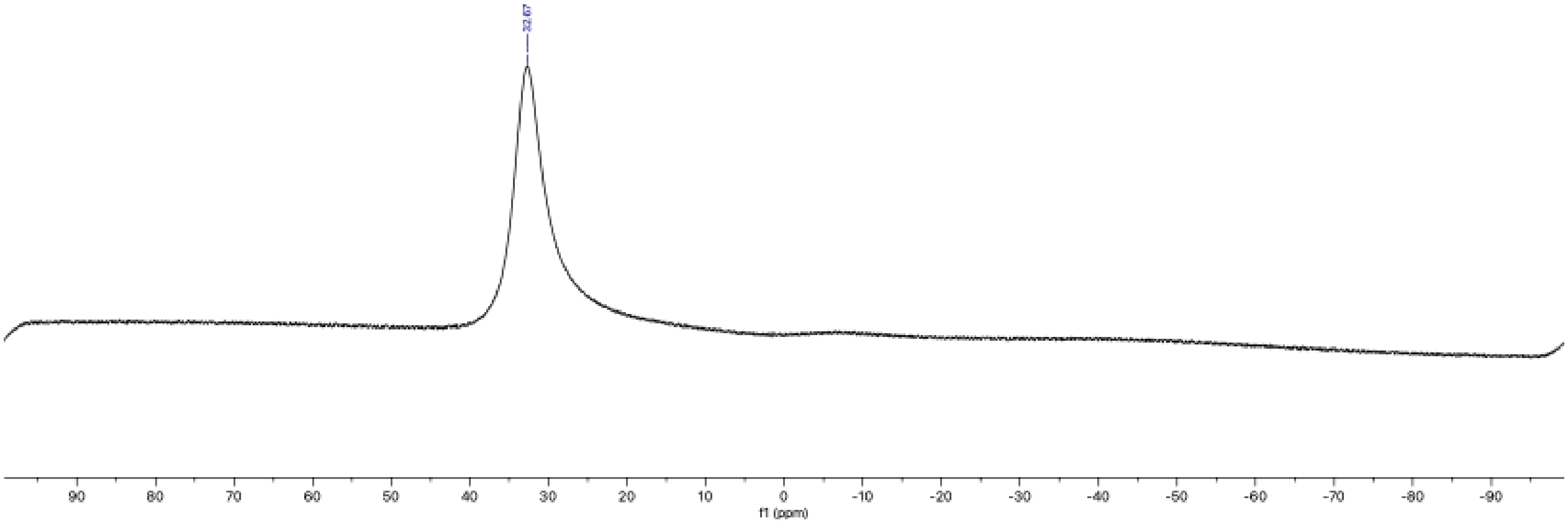

^{11}B NMR spectrum (128 MHz, $CDCl_3$)

((1*S,2*R**,3*S**)-2-Butyl-3-(3-chloropropyl)-2-(4,4,5,5-tetramethyl-1,3,2-dioxaborolan-2-yl)cyclopropyl)methanol (5i-OH)**

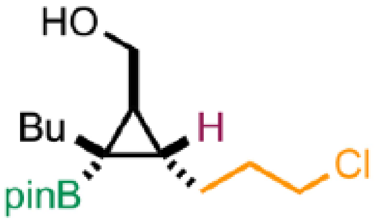

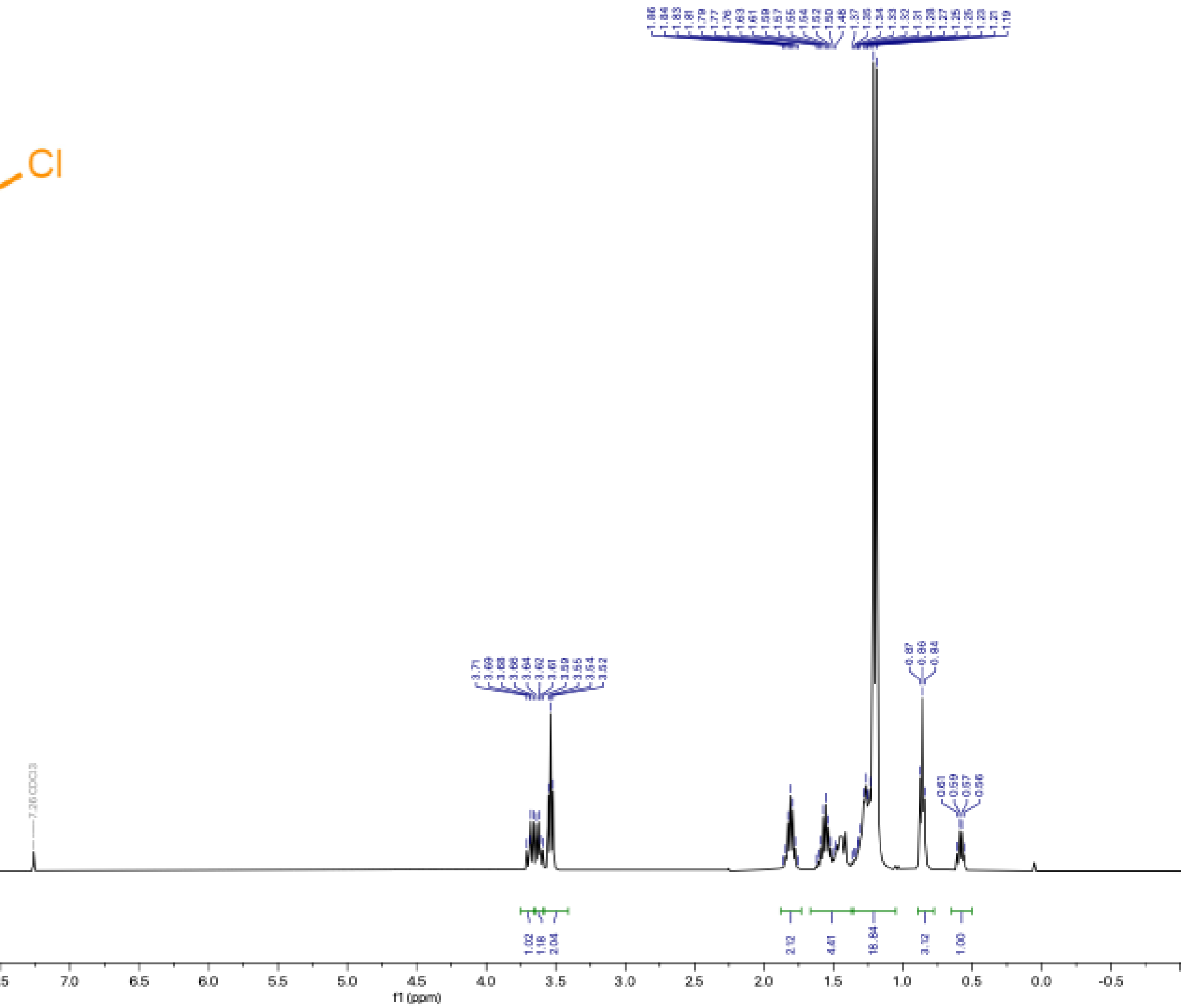

^{1}H NMR spectrum (400 MHz, $CDCl_3$)

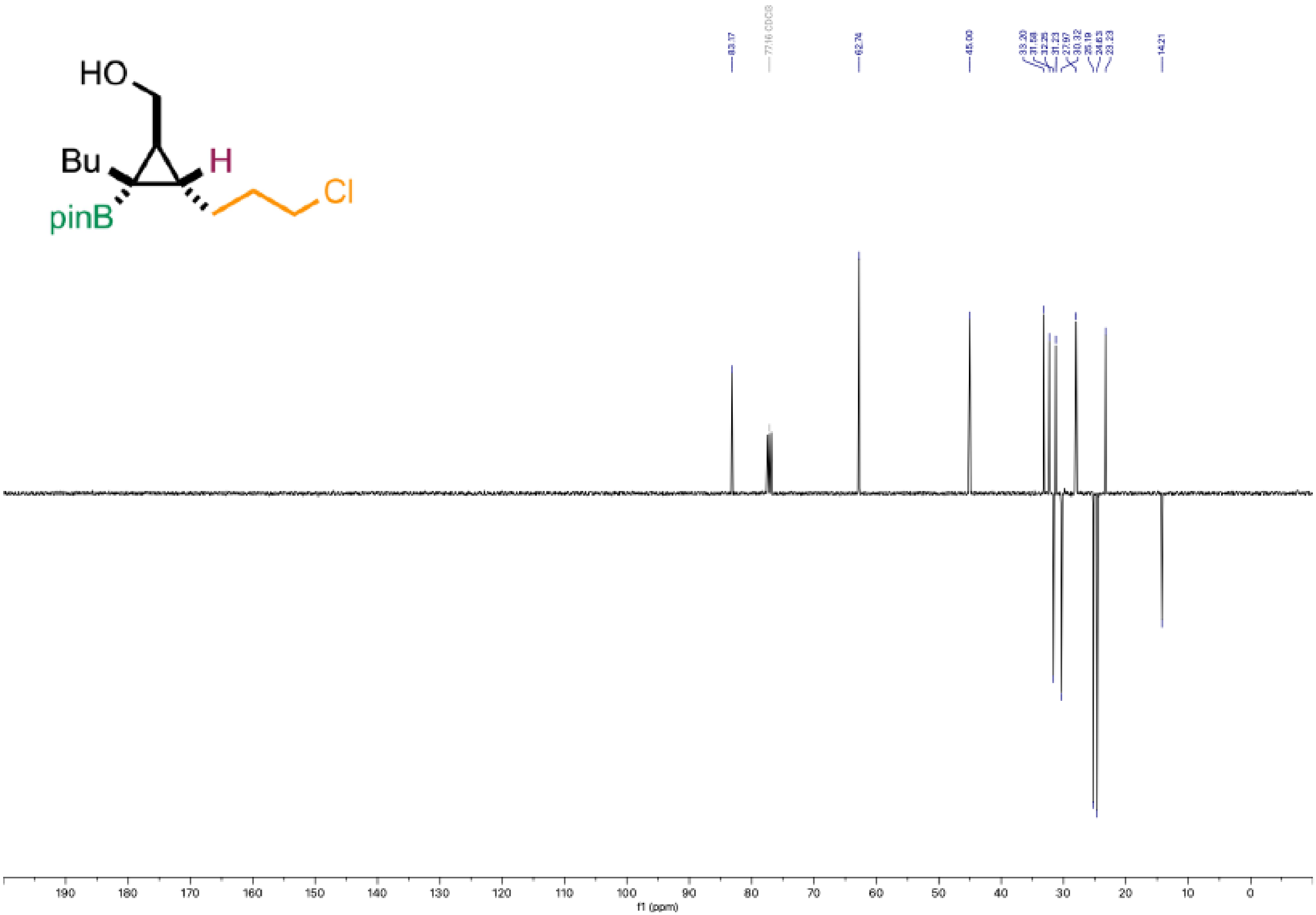

^{13}C NMR spectrum (101 MHz, $CDCl_3$)

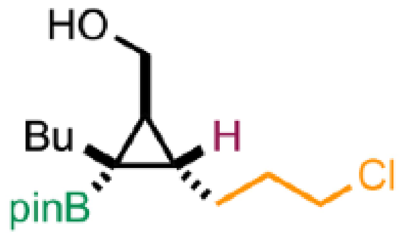

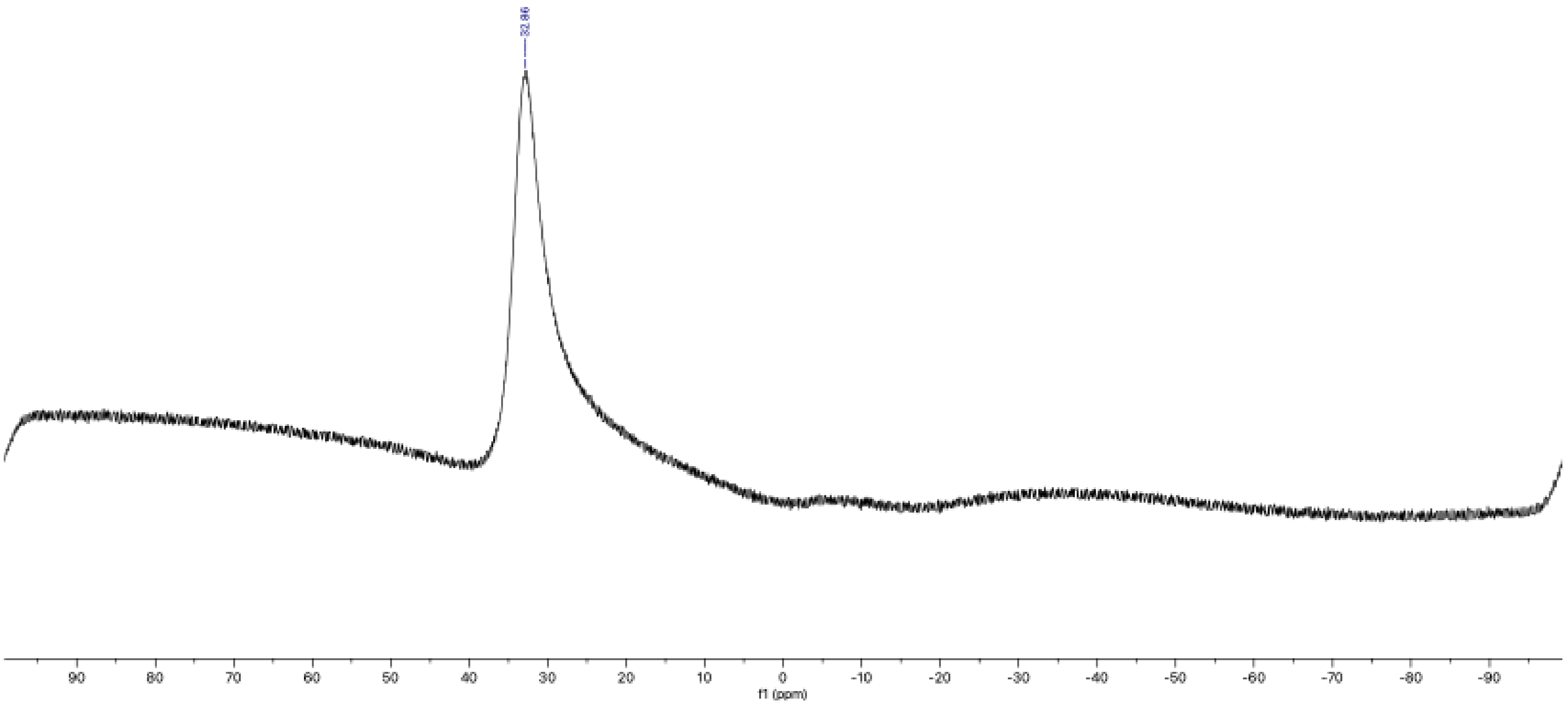

^{11}B NMR spectrum (128 MHz, $CDCl_3$)

Trimethyl((2*R,3*R**)-3-methyl-2-(4,4,5,5-tetramethyl-1,3,2-dioxaborolan-2-yl)pent-4-en-2-yl)silane (6a)**

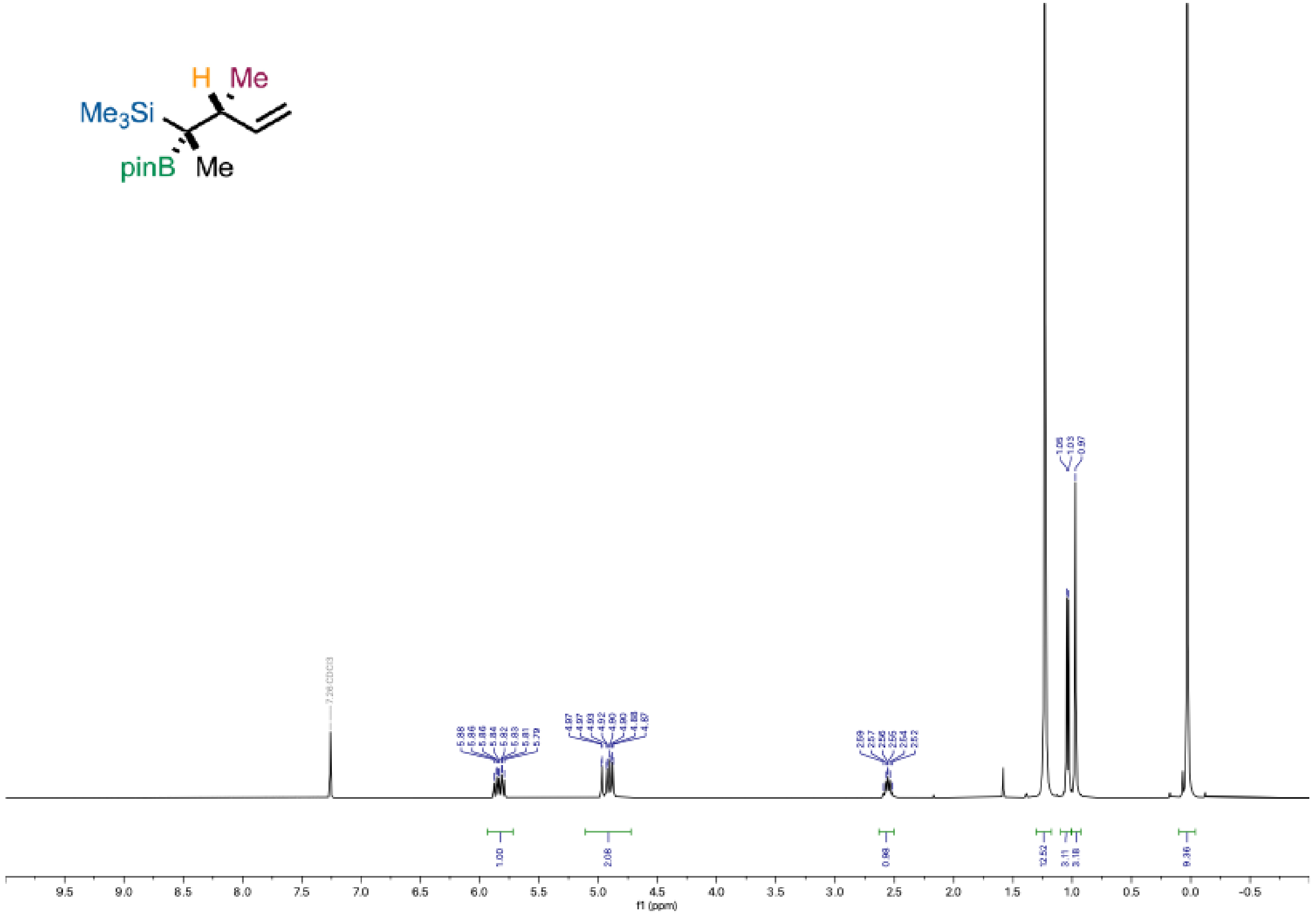

^{1}H NMR spectrum (400 MHz, $CDCl_3$)

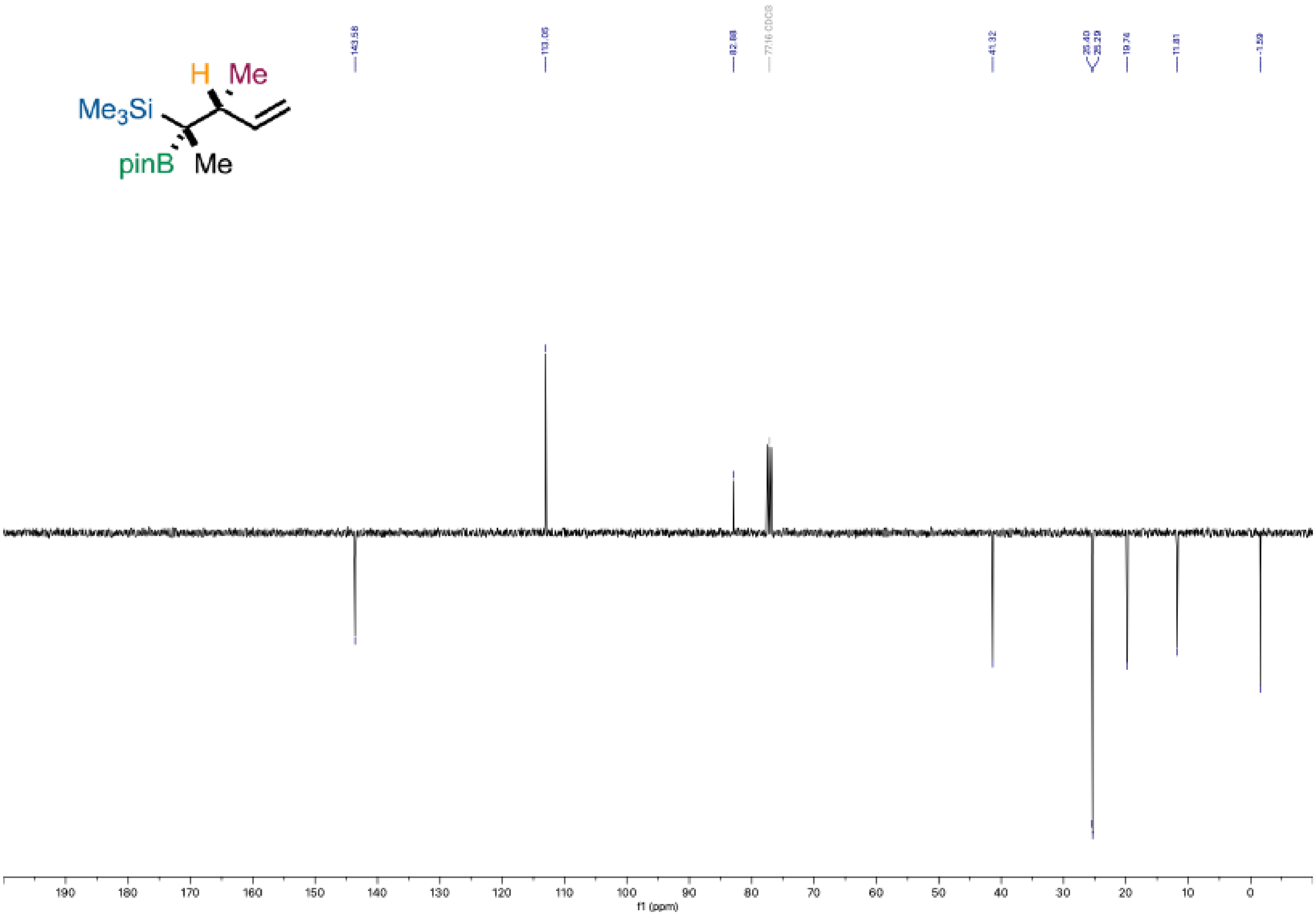

^{13}**C NMR** spectrum (101 MHz, $CDCl_3$)

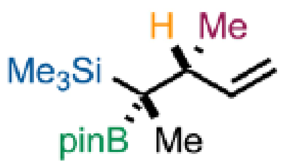

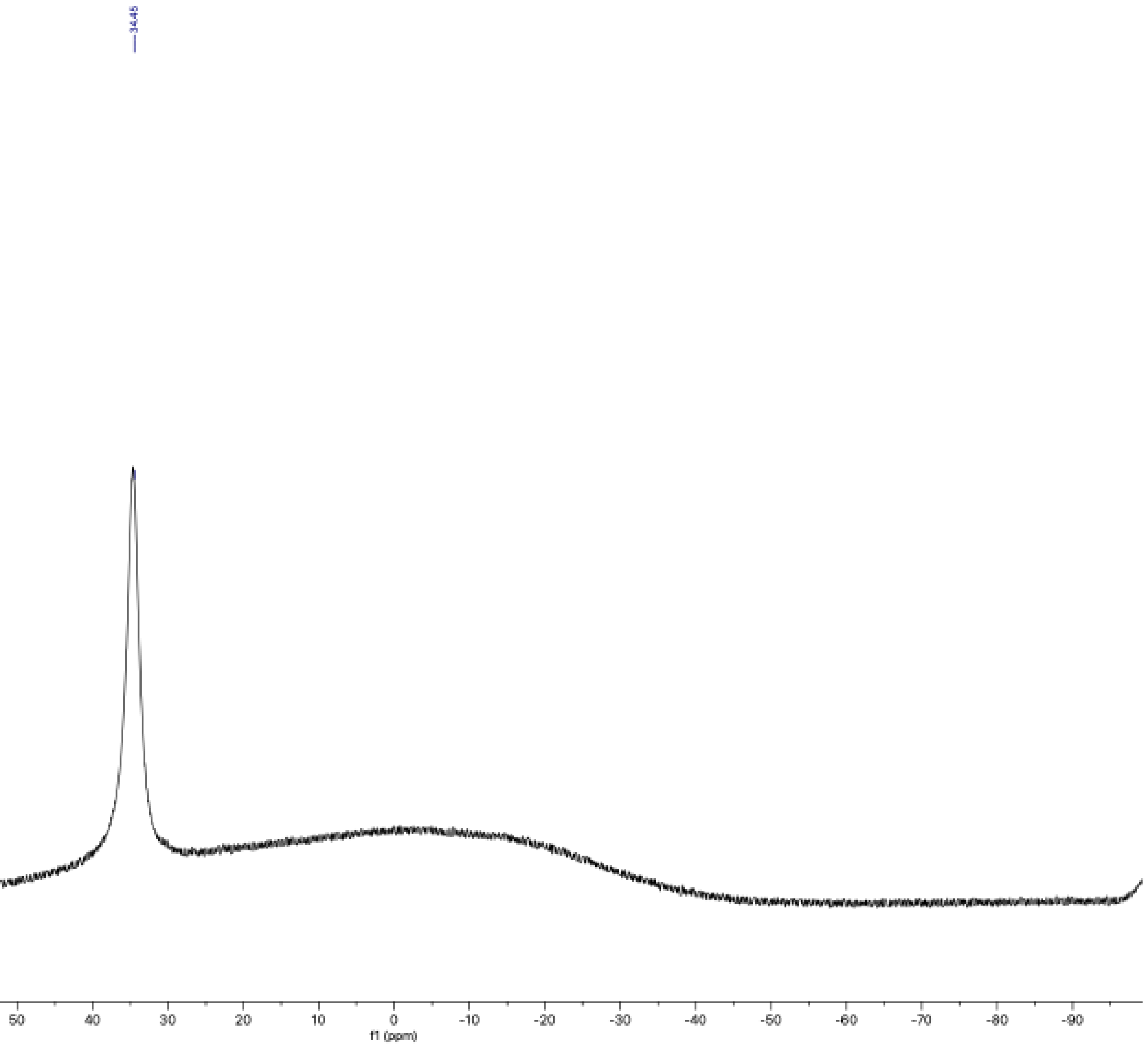

^{11}B NMR spectrum (128 MHz, $CDCl_3$)

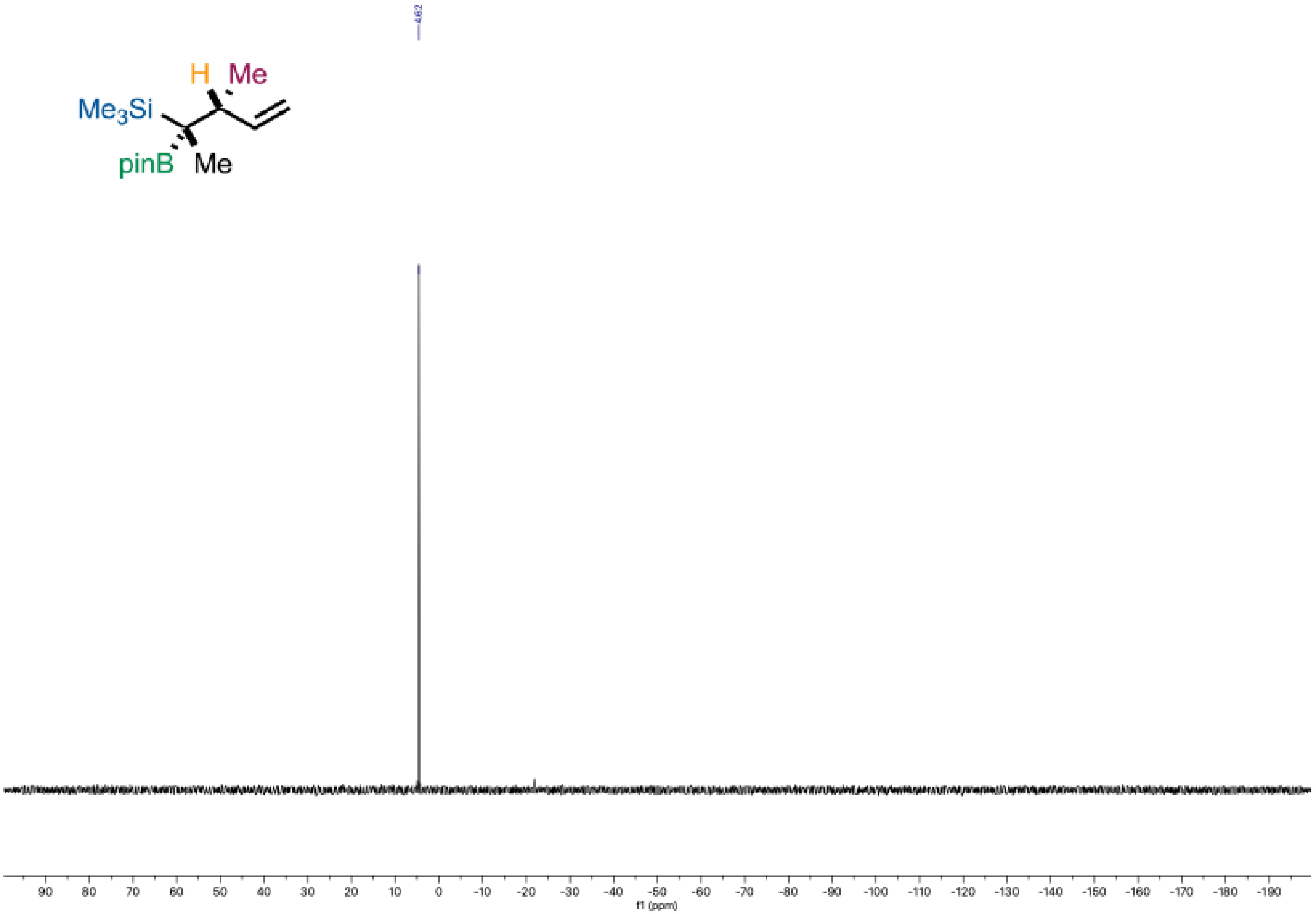

^{29}**Si NMR** spectrum (80 MHz, $CDCl_3$)

Trimethyl((2*R,3*R**)-3-methyl-2-(4,4,5,5-tetramethyl-1,3,2-dioxaborolan-2-yl)pent-4-en-2-yl)silane (6b)**

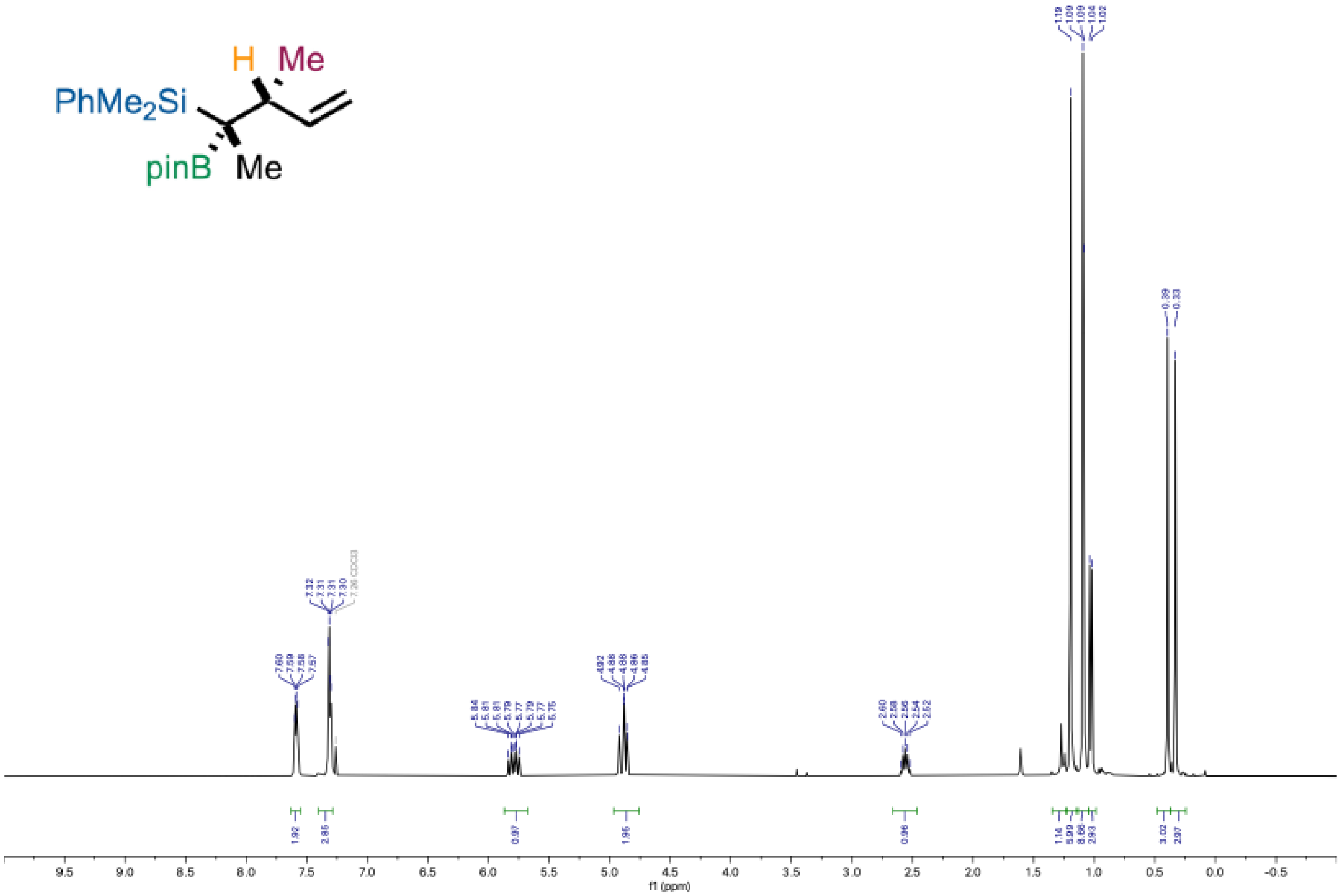

¹H NMR spectrum (400 MHz, $CDCl_3$)

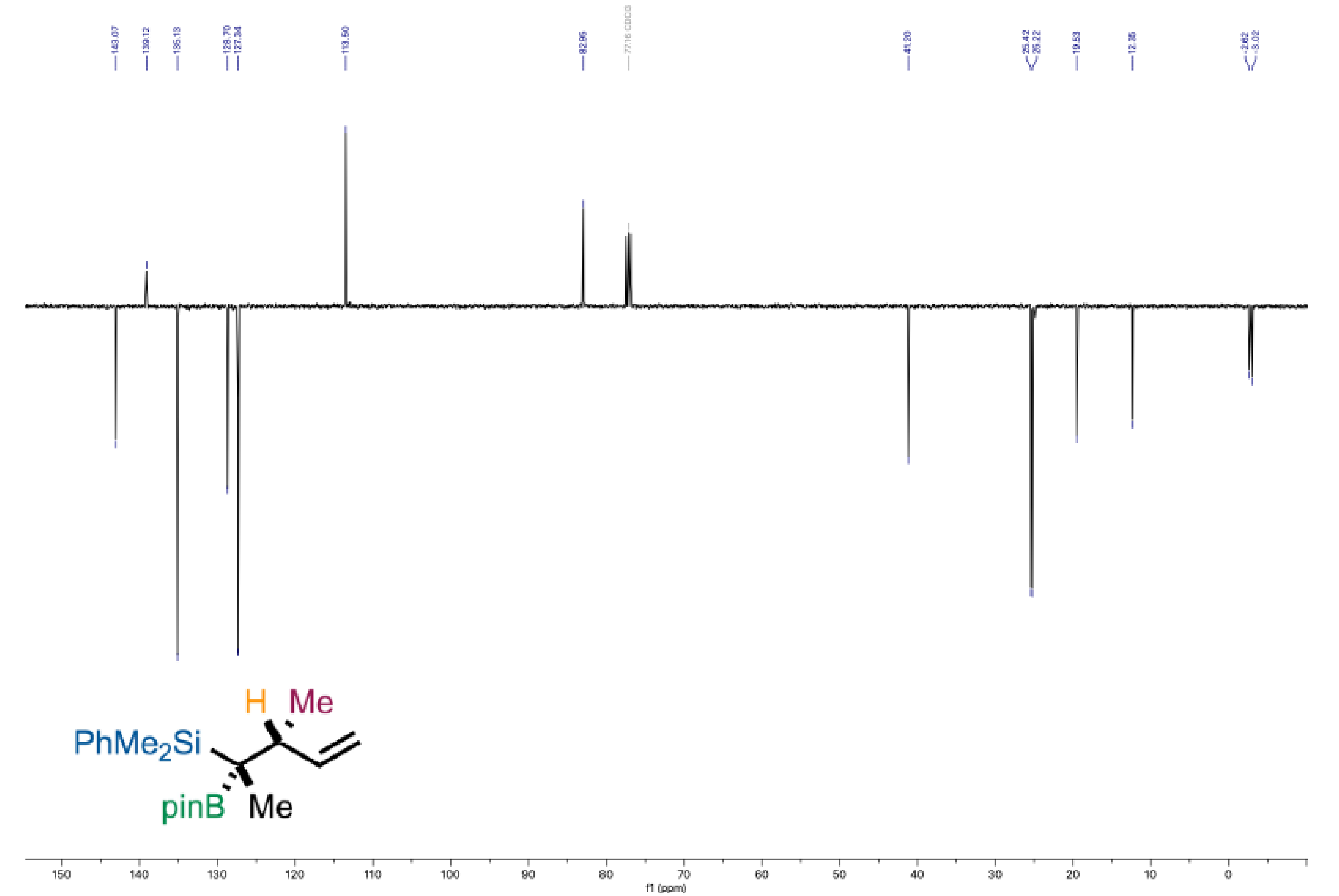

^{13}**C NMR** spectrum (101 MHz, $CDCl_3$)

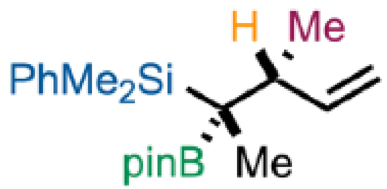

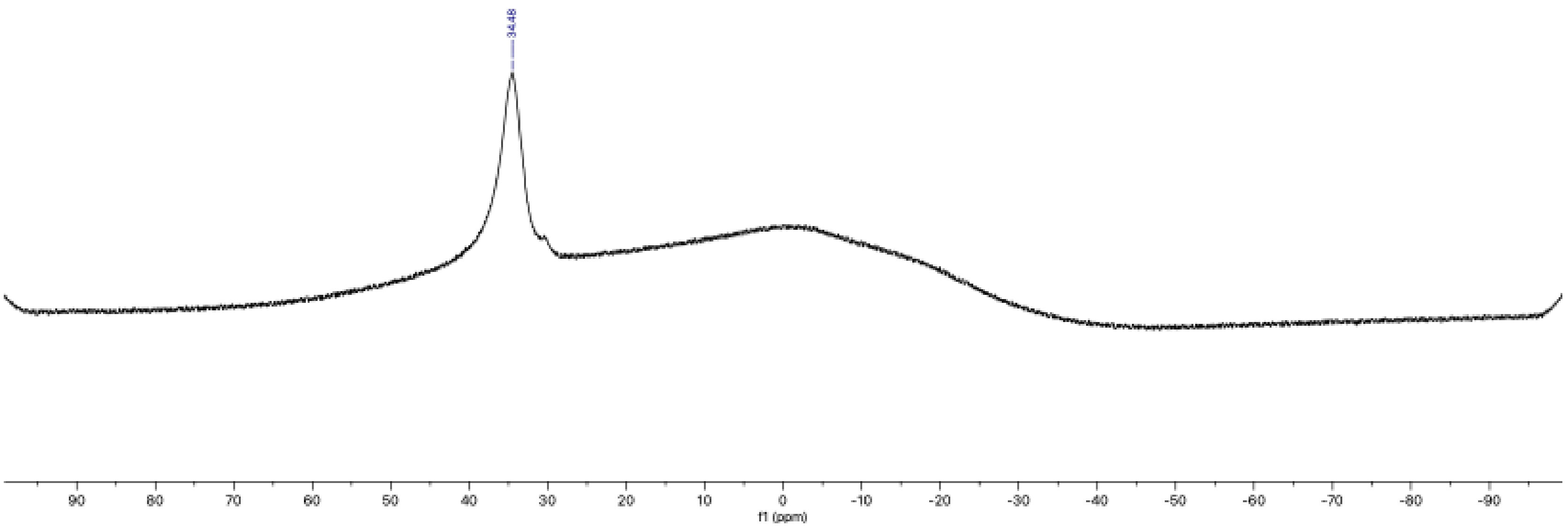

^{11}B NMR spectrum (128 MHz, $CDCl_3$)

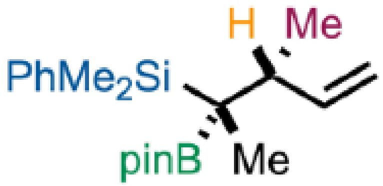

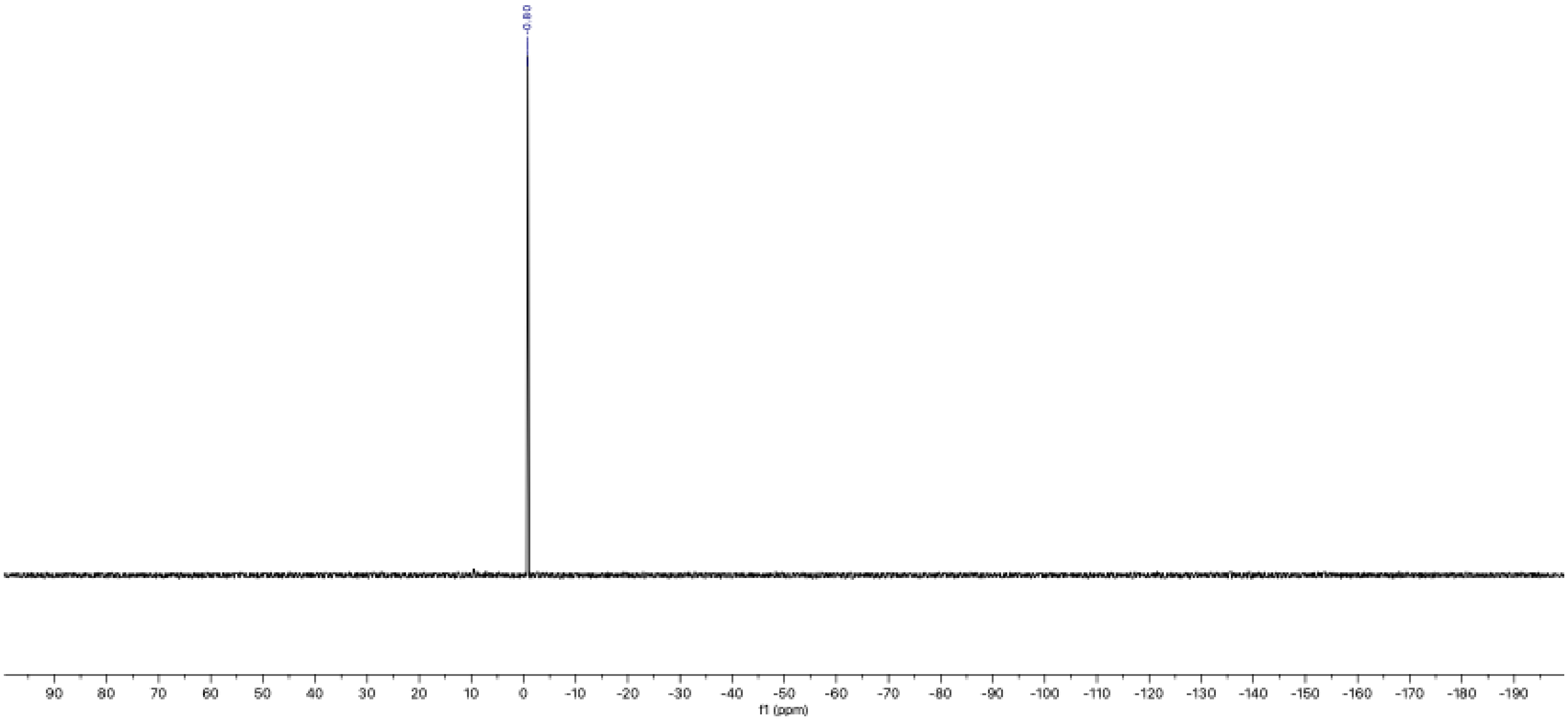

[29]**Si NMR** spectrum (80 MHz, $CDCl_3$)

Trimethyl((2*S,3*S**)-2-(4,4,5,5-tetramethyl-1,3,2-dioxaborolan-2-yl)-3-vinylhex-5-en-2-yl)silane (6c)**

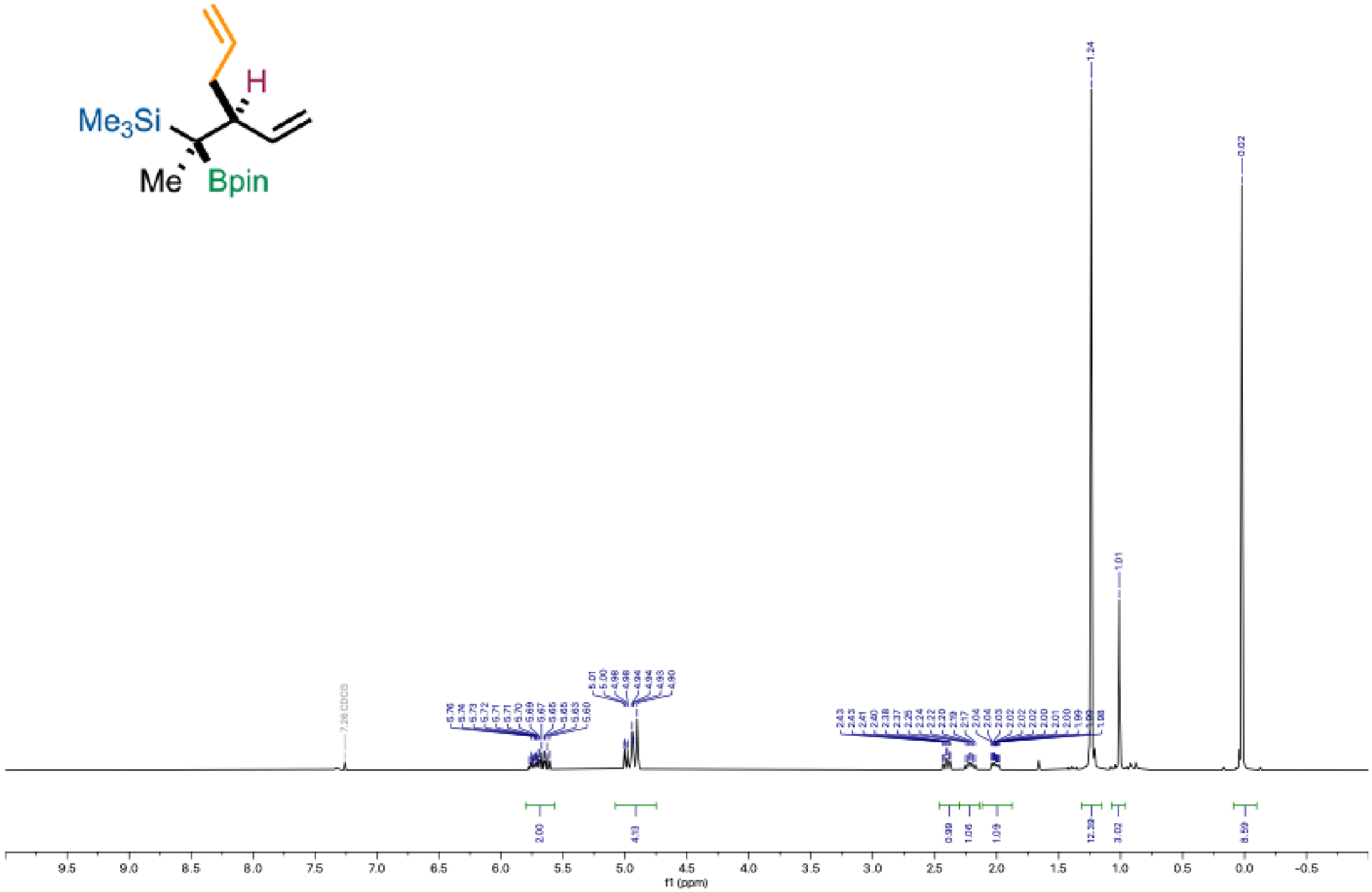

¹H NMR spectrum (400 MHz, $CDCl_3$)

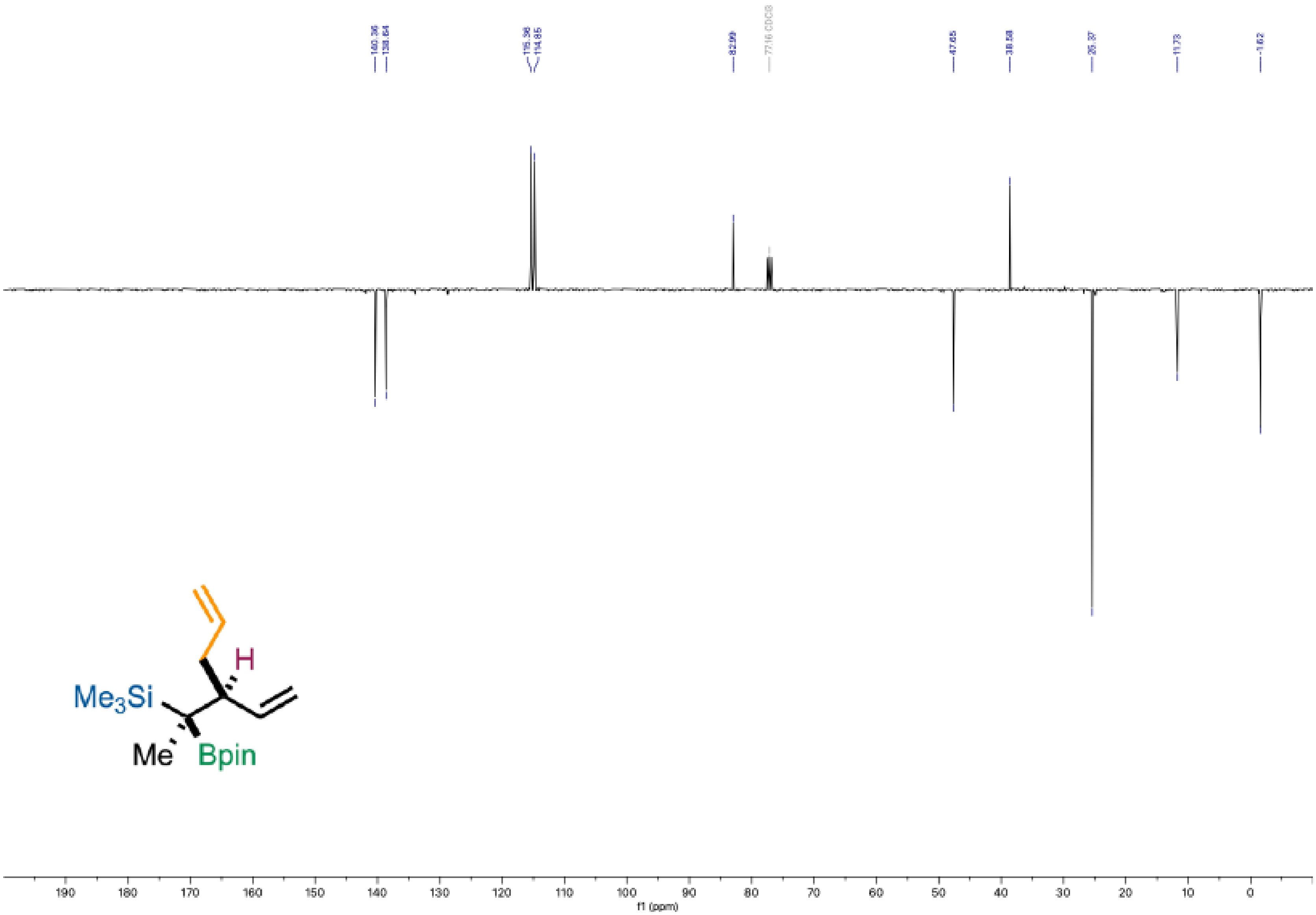

^{13}C NMR spectrum (101 MHz, $CDCl_3$)

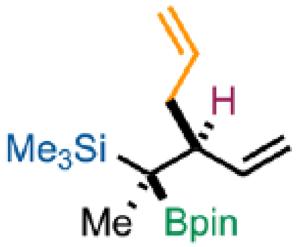

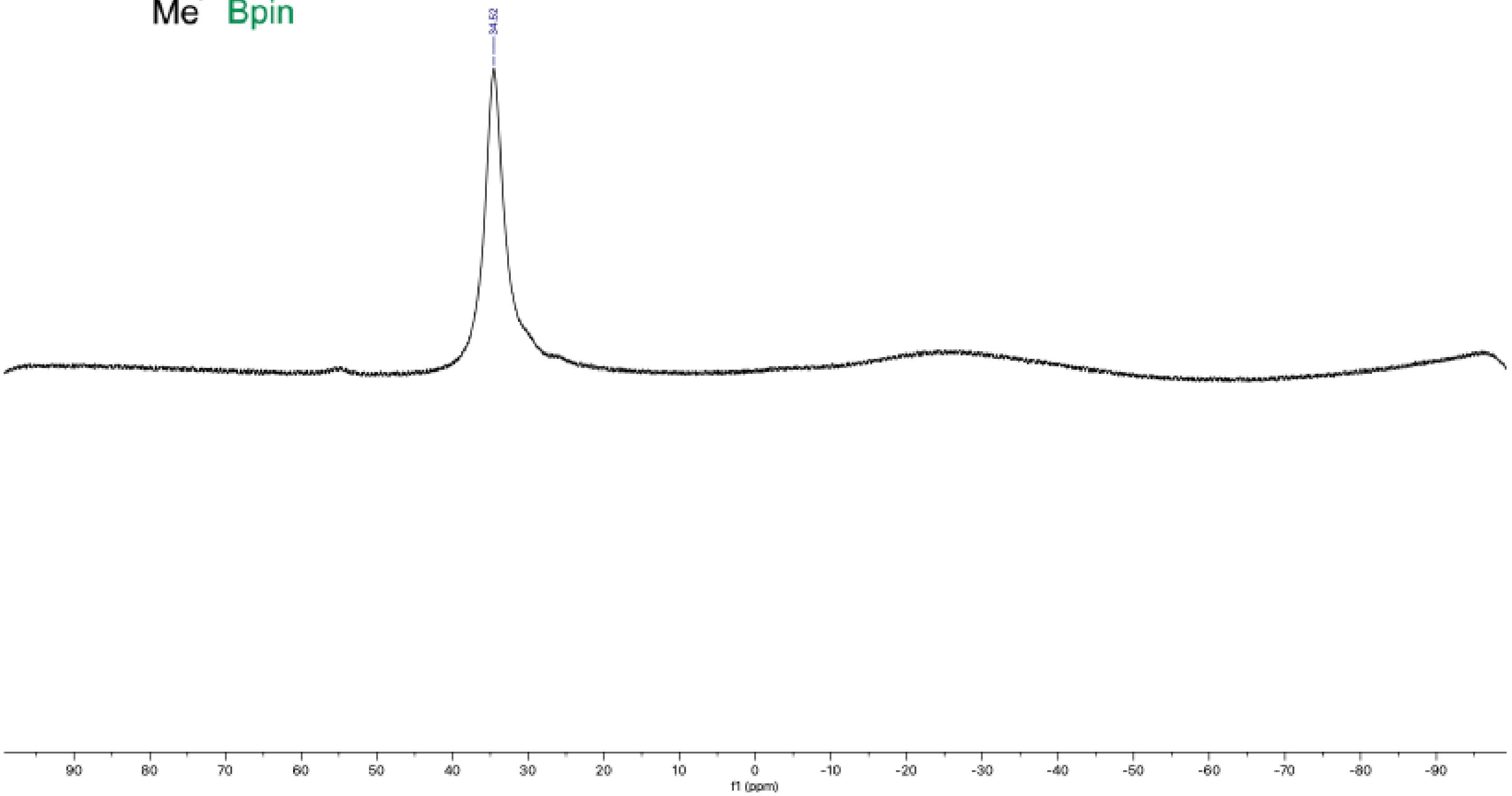

^{11}B NMR spectrum (128 MHz, $CDCl_3$)

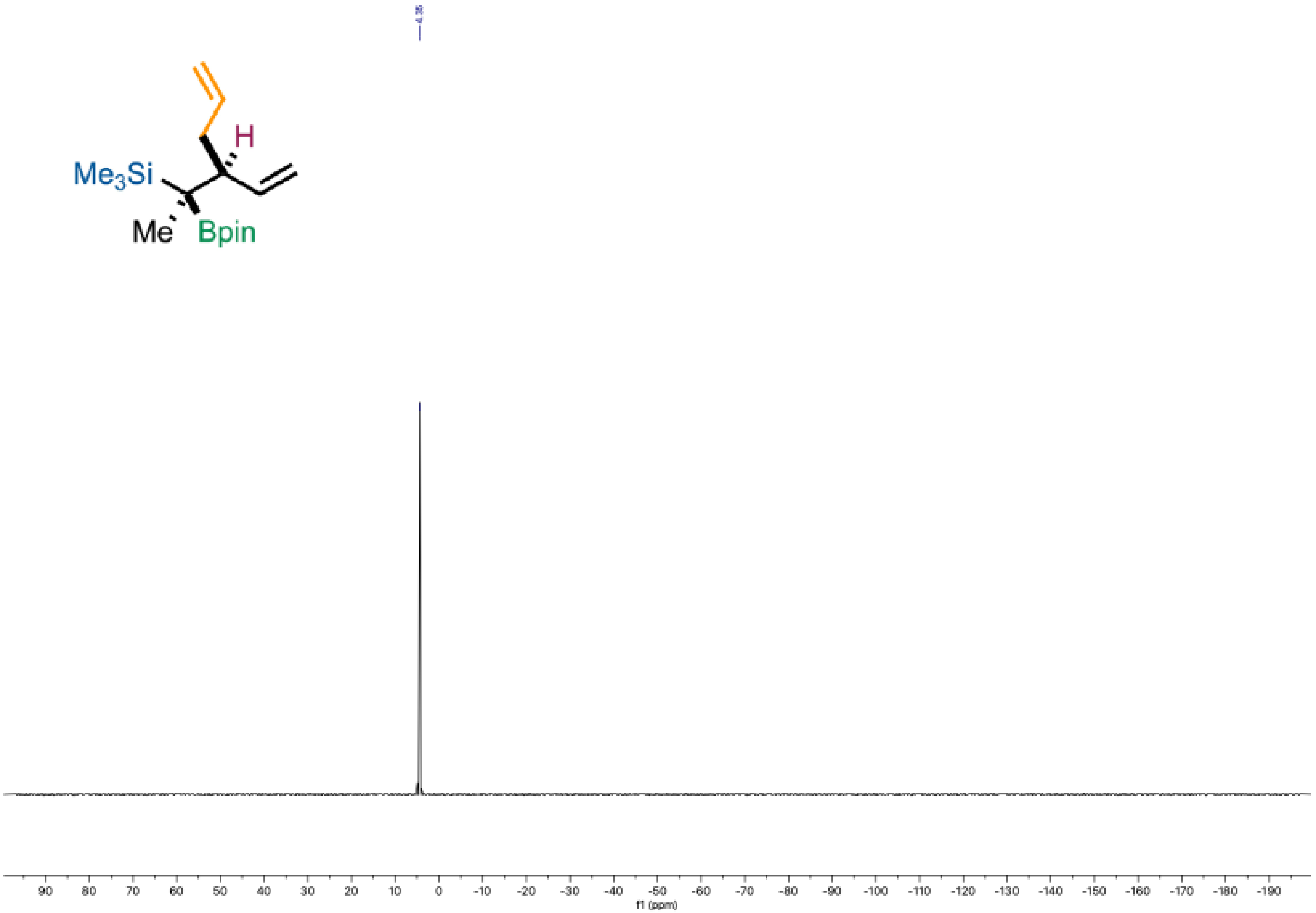

^{29}Si NMR spectrum (80 MHz, $CDCl_3$)

Trimethyl((4*R,5*R**)-4-(4,4,5,5-tetramethyl-1,3,2-dioxaborolan-2-yl)-5-vinyloctan-4-yl)silane (6d)**

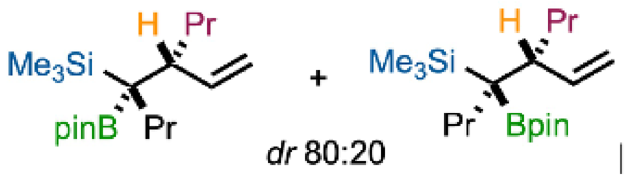

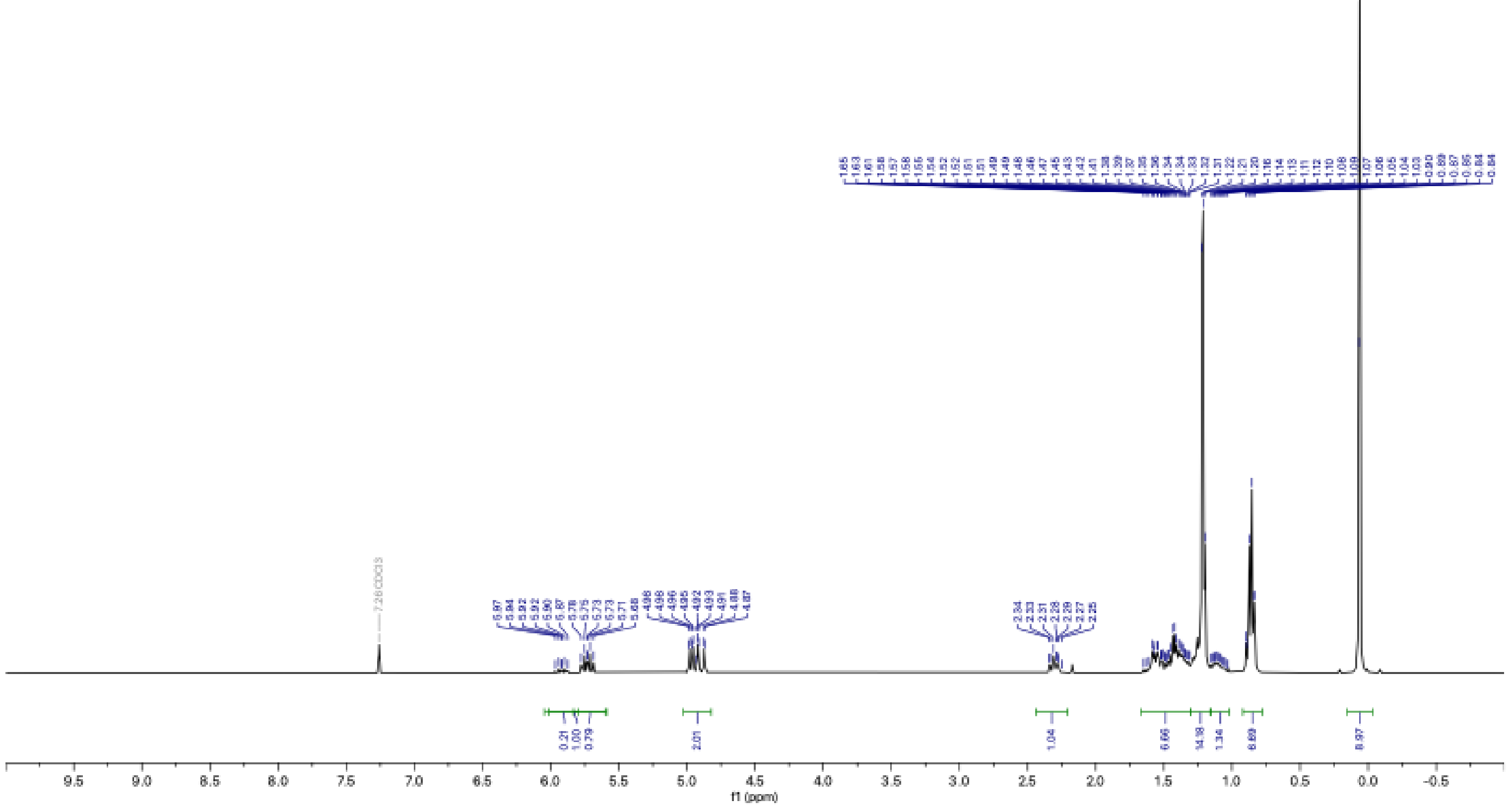

[1]**H NMR** spectrum (400 MHz, $CDCl_3$)

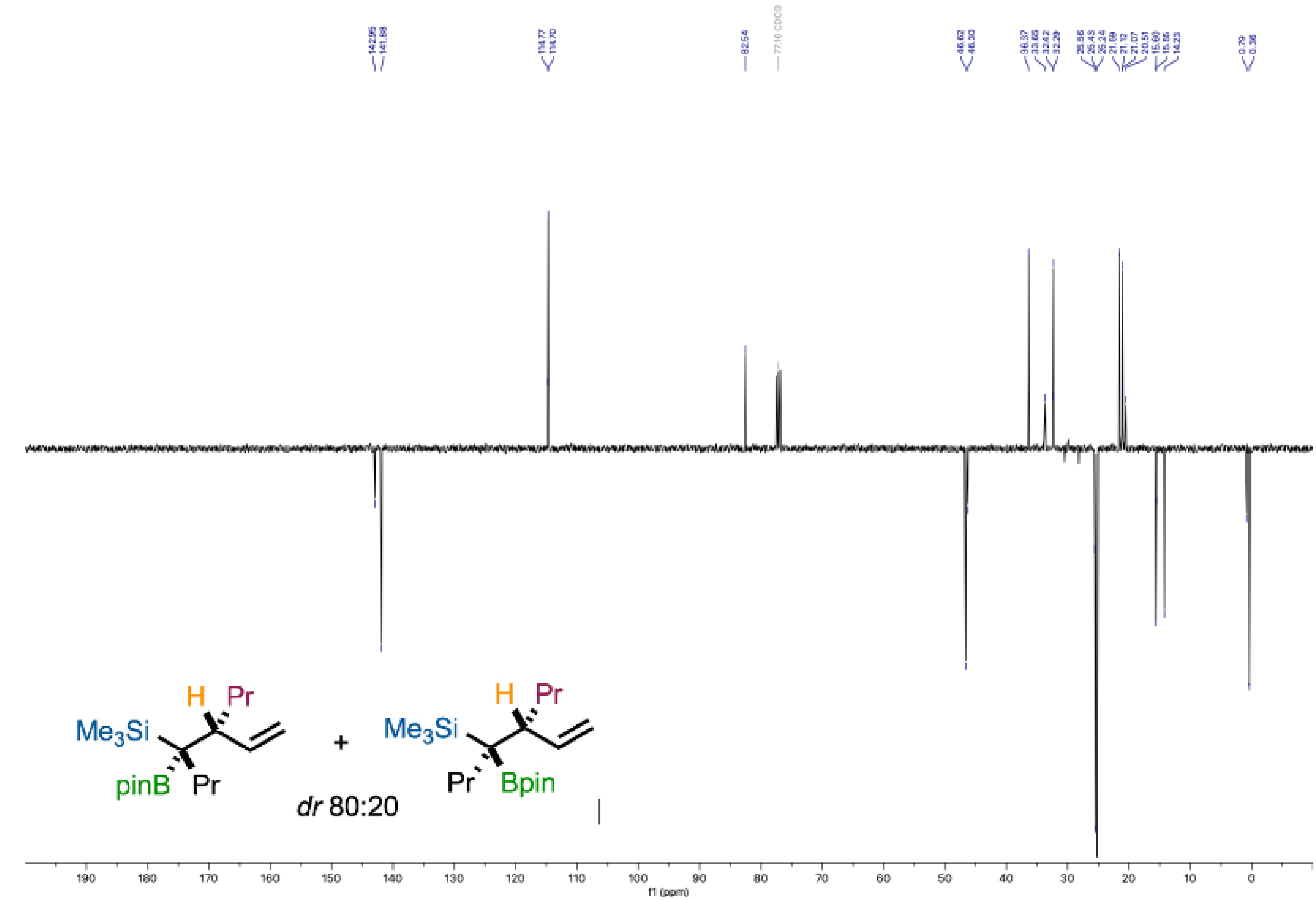

^{13}C NMR spectrum (101 MHz, $CDCl_3$)

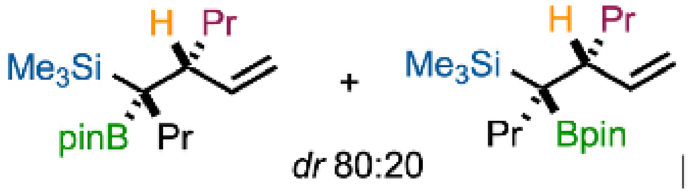

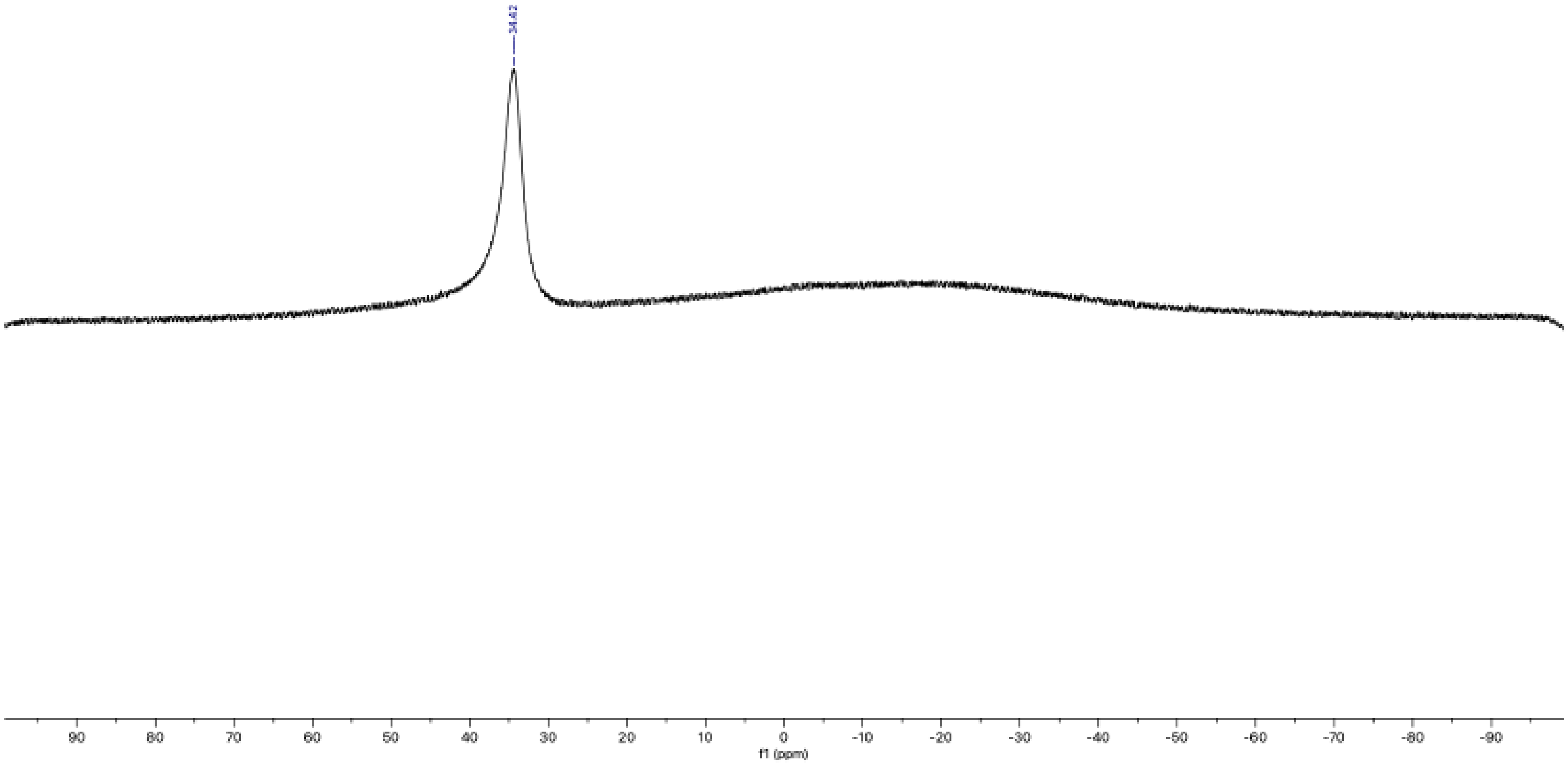

^{11}B NMR spectrum (128 MHz, $CDCl_3$)

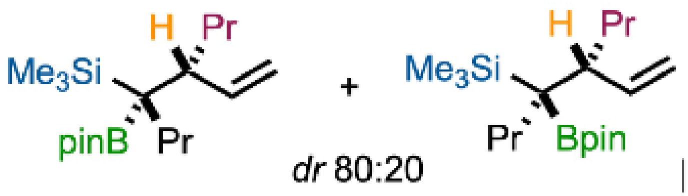

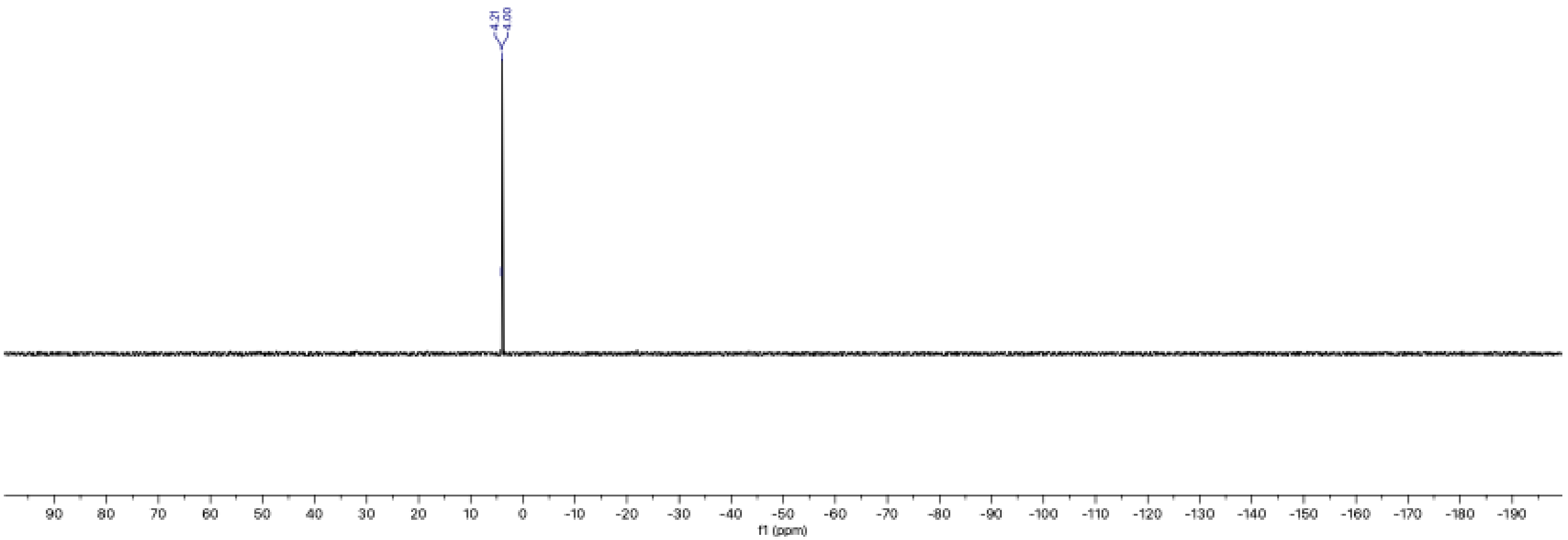

^{29}Si NMR spectrum (80 MHz, $CDCl_3$)

((3*R,4*R**)-4-Ethyl-3-(4,4,5,5-tetramethyl-1,3,2-dioxaborolan-2-yl)hex-5-en-3-yl)trimethylsilane (6e)**

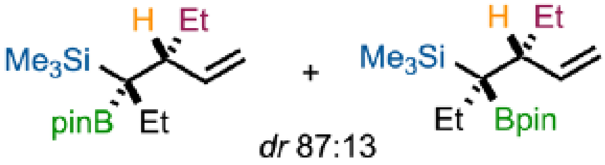

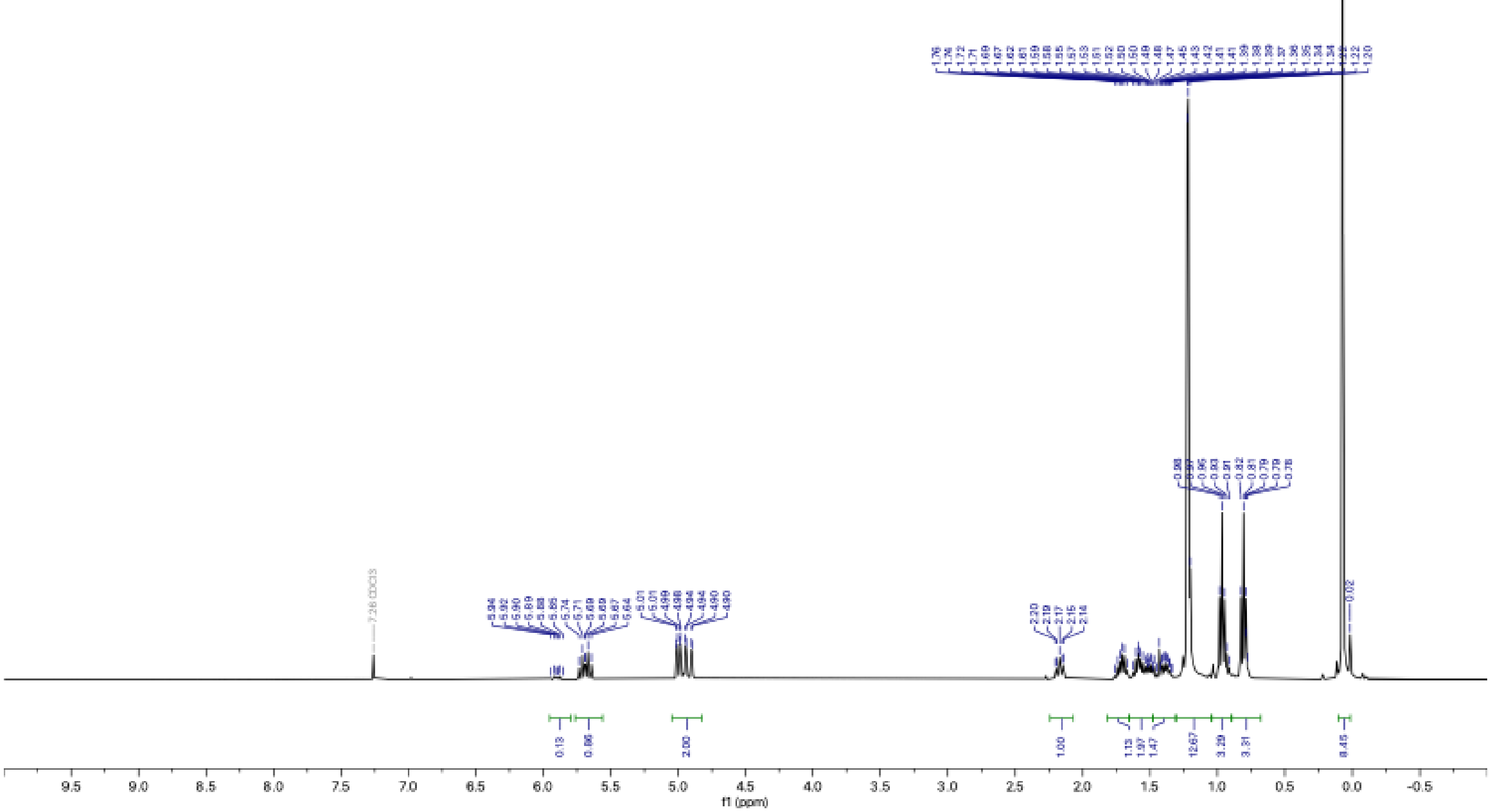

^{1}H NMR spectrum (400 MHz, $CDCl_3$)

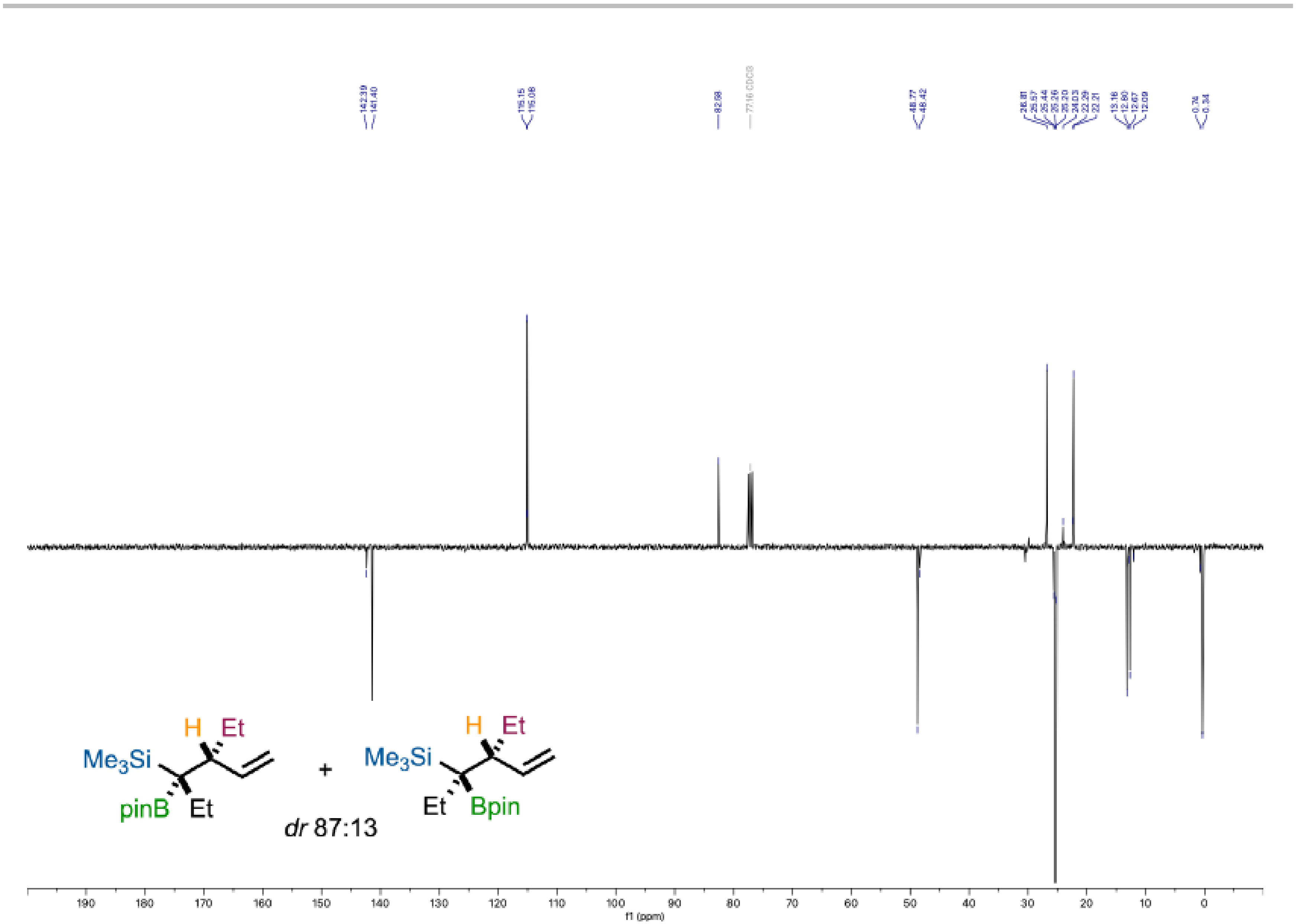

^{13}**C NMR** spectrum (101 MHz, $CDCl_3$)

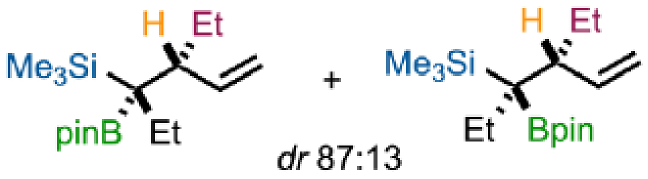

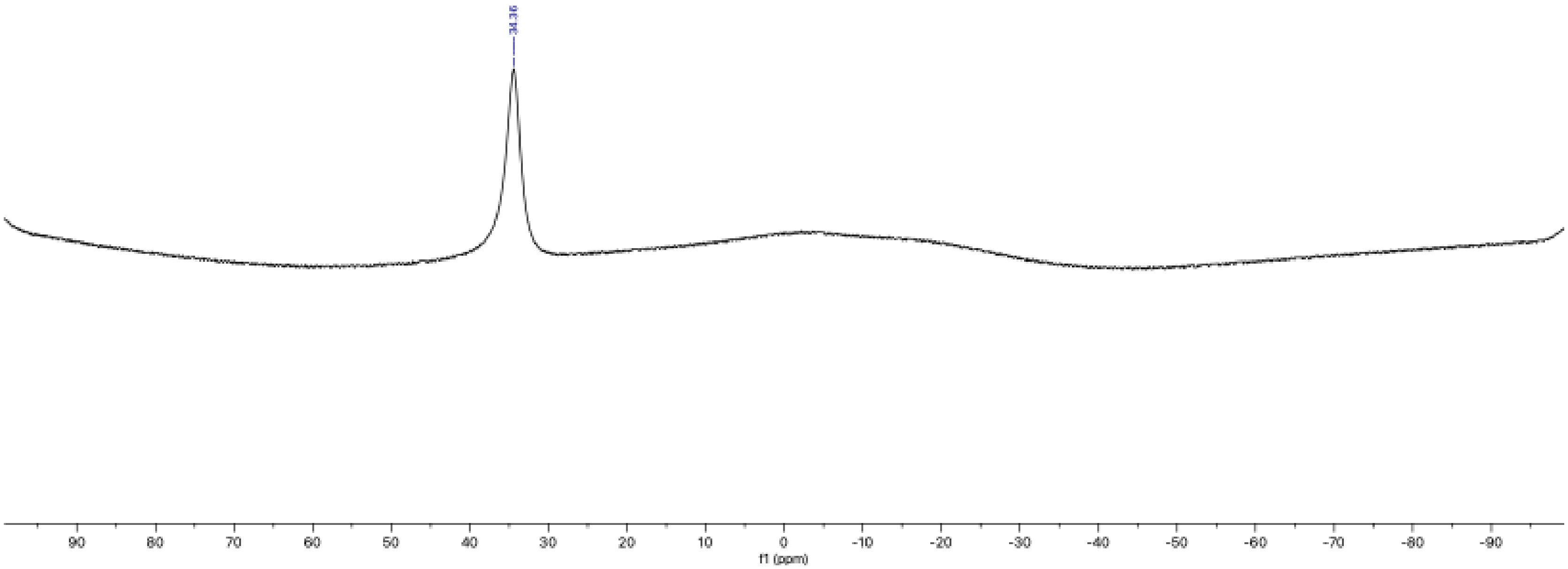

^{11}B NMR spectrum (128 MHz, $CDCl_3$)

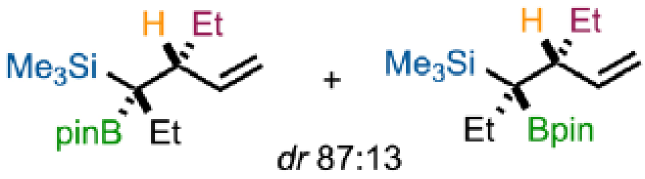

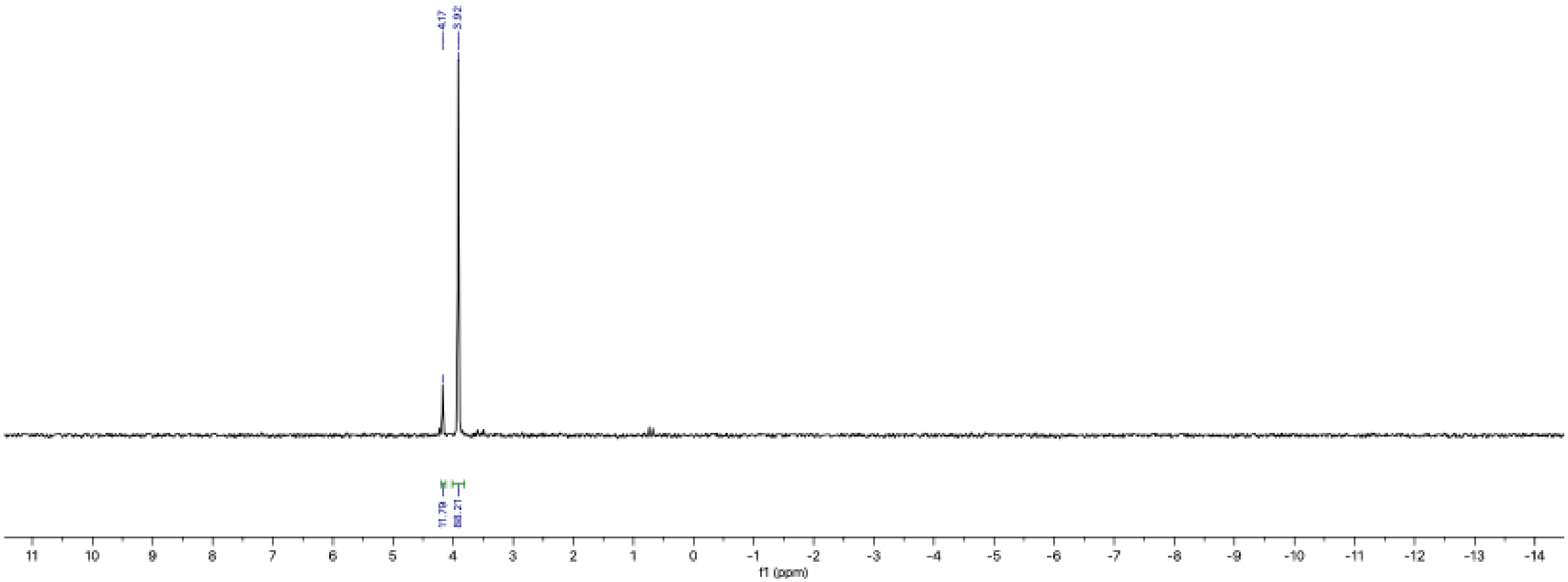

^{29}Si NMR spectrum (80 MHz, $CDCl_3$)

Trimethyl((4*S,5*S**)-5-(4,4,5,5-tetramethyl-1,3,2-dioxaborolan-2-yl)-4-vinylnon-1-en-5-yl)silane (6f)**

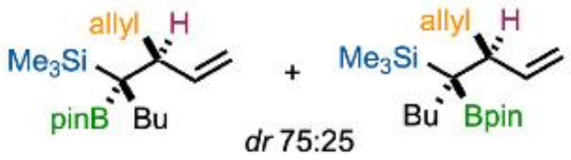

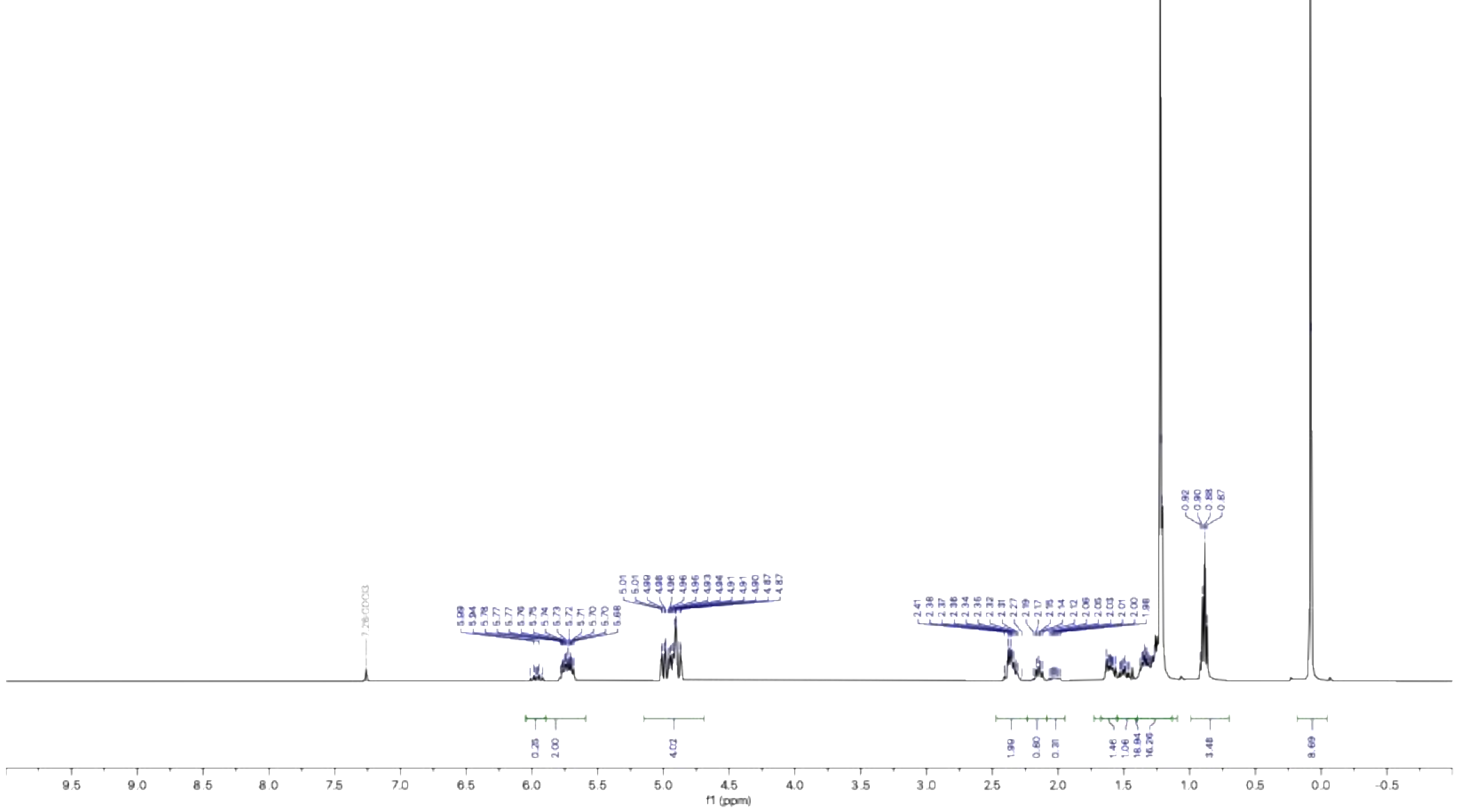

^{1}H NMR spectrum (400 MHz, $CDCl_3$)

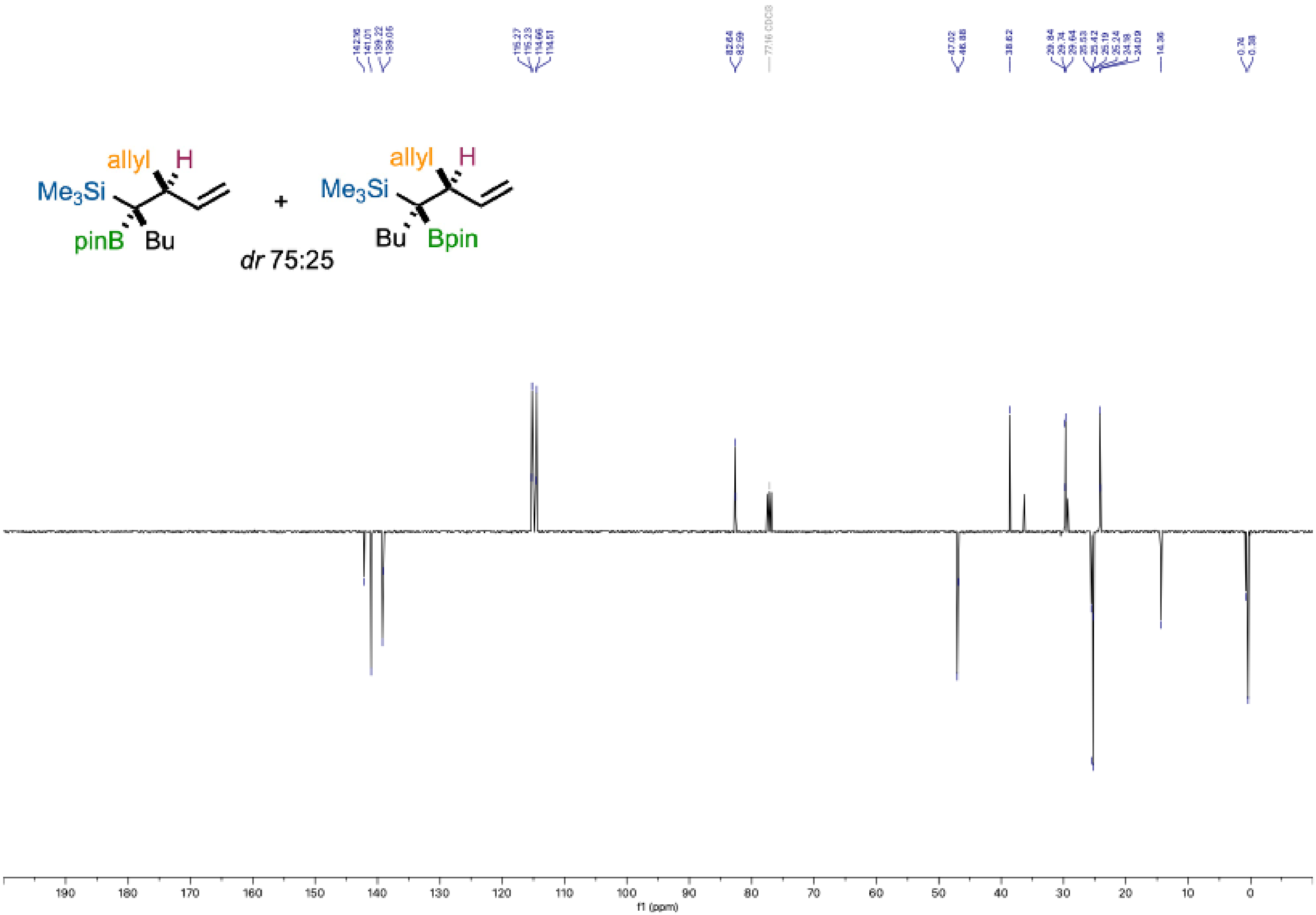

^{13}C NMR spectrum (101 MHz, $CDCl_3$)

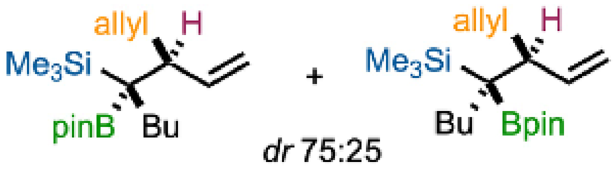

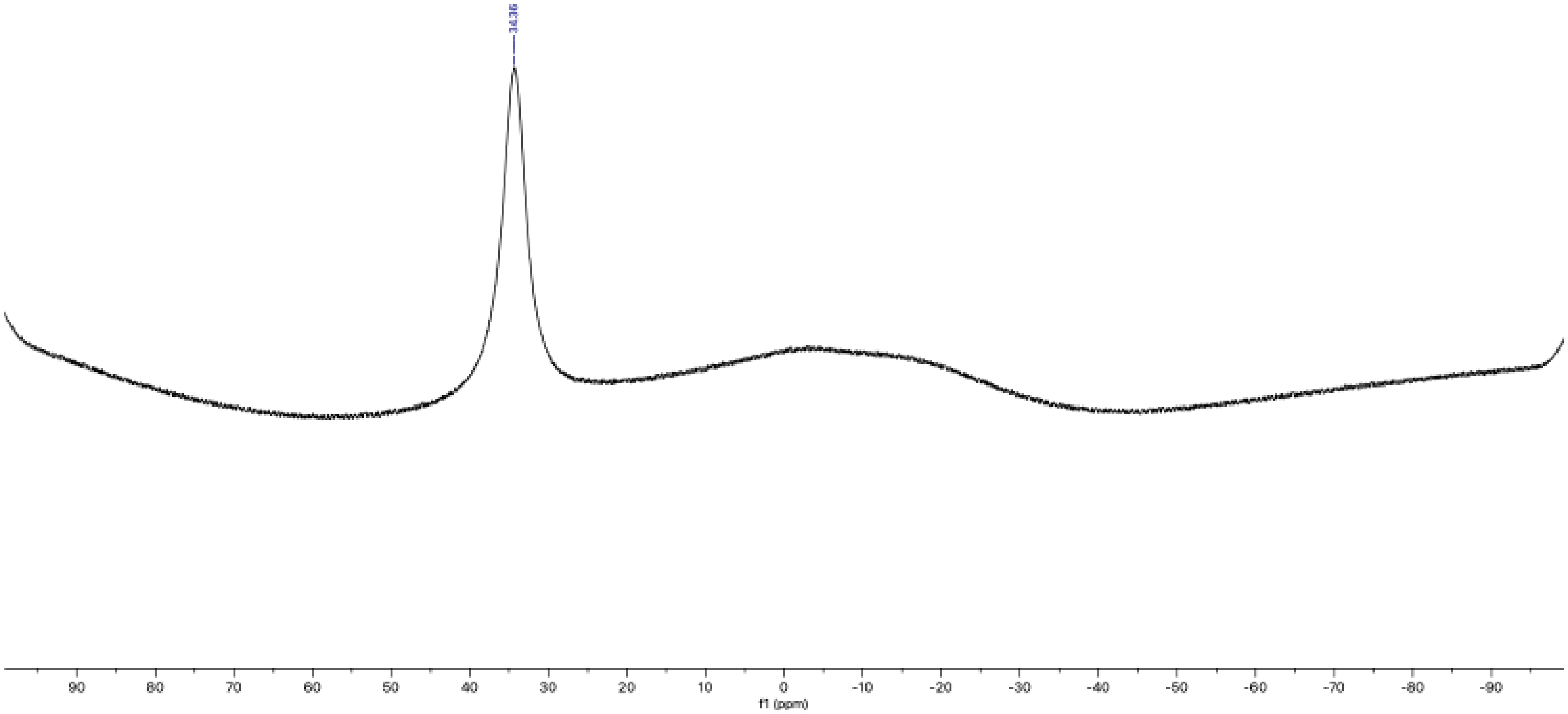

^{11}B NMR spectrum (128 MHz, $CDCl_3$)

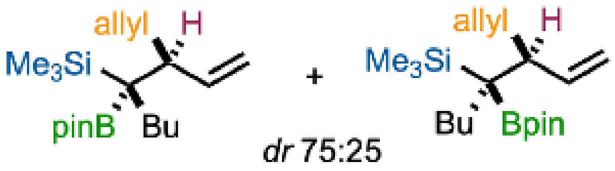

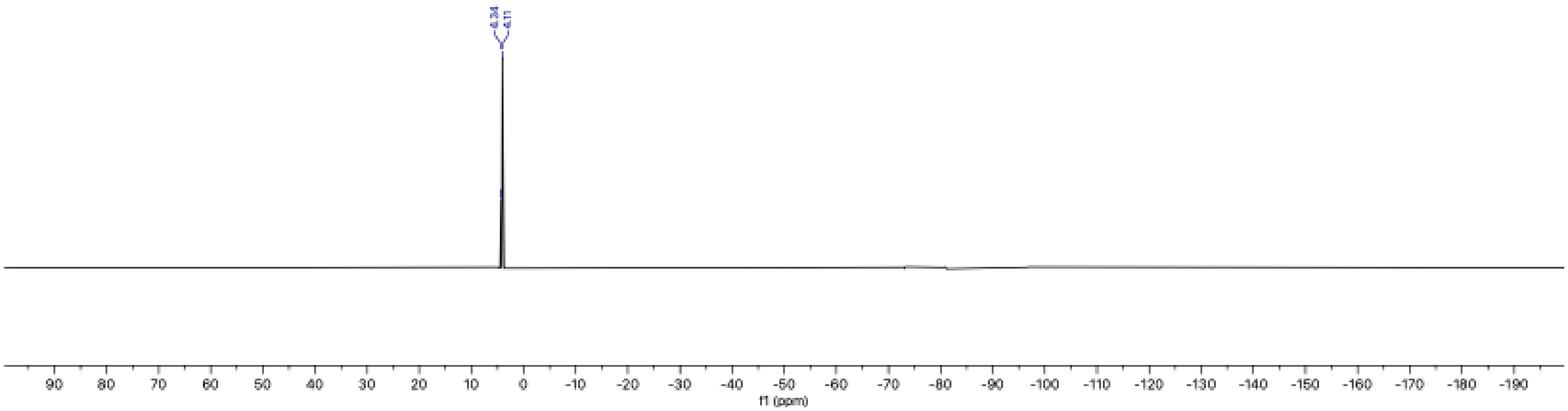

29Si NMR spectrum (80 MHz, $CDCl_3$)

((1*S,2*S**)-1-Cyclohexyl-1-(4,4,5,5-tetramethyl-1,3,2-dioxaborolan-2-yl)-2-vinylpentyl)trimethylsilane 6g**

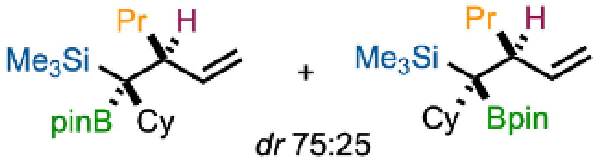

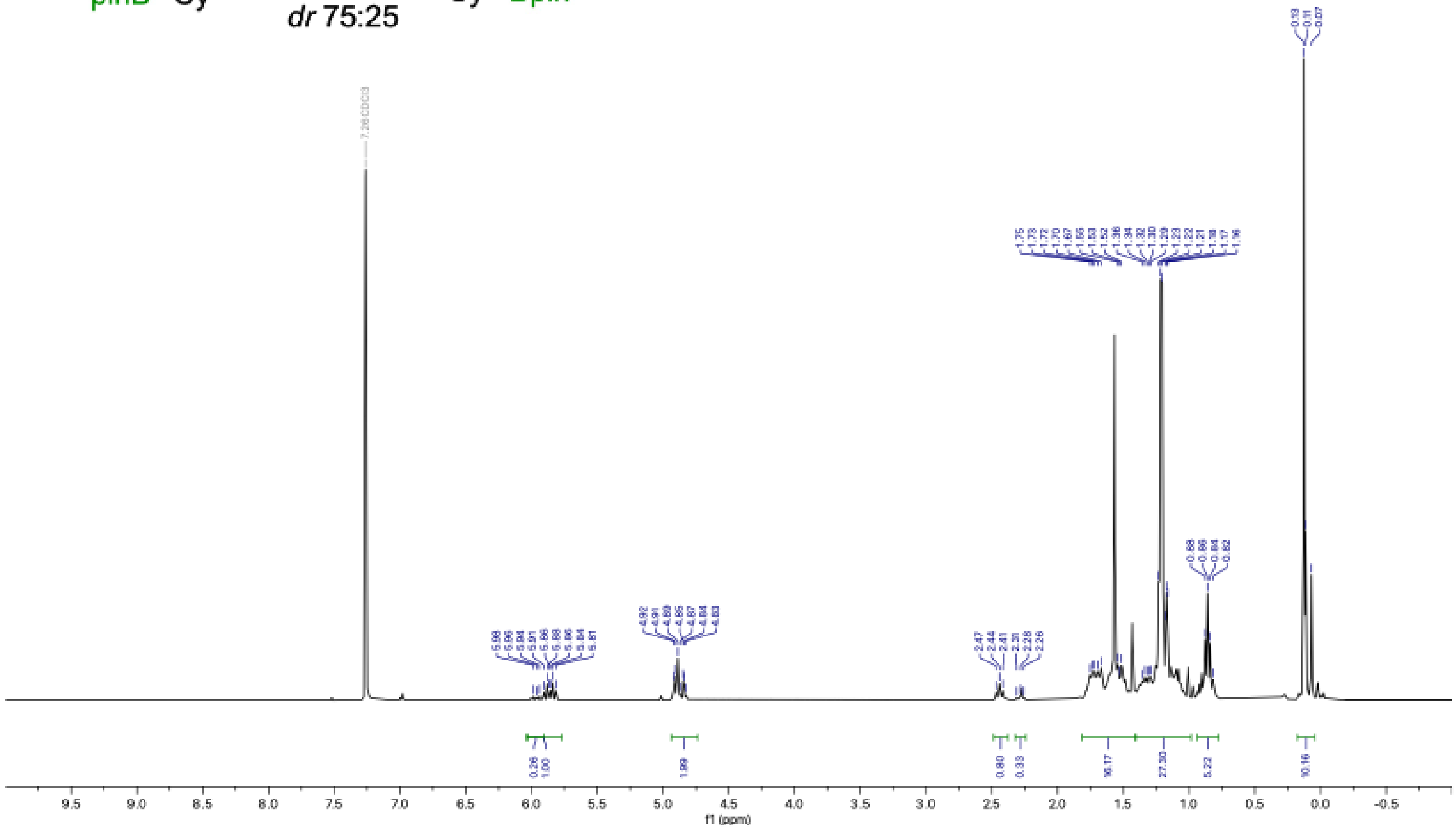

^{1}H NMR spectrum (400 MHz, $CDCl_3$)

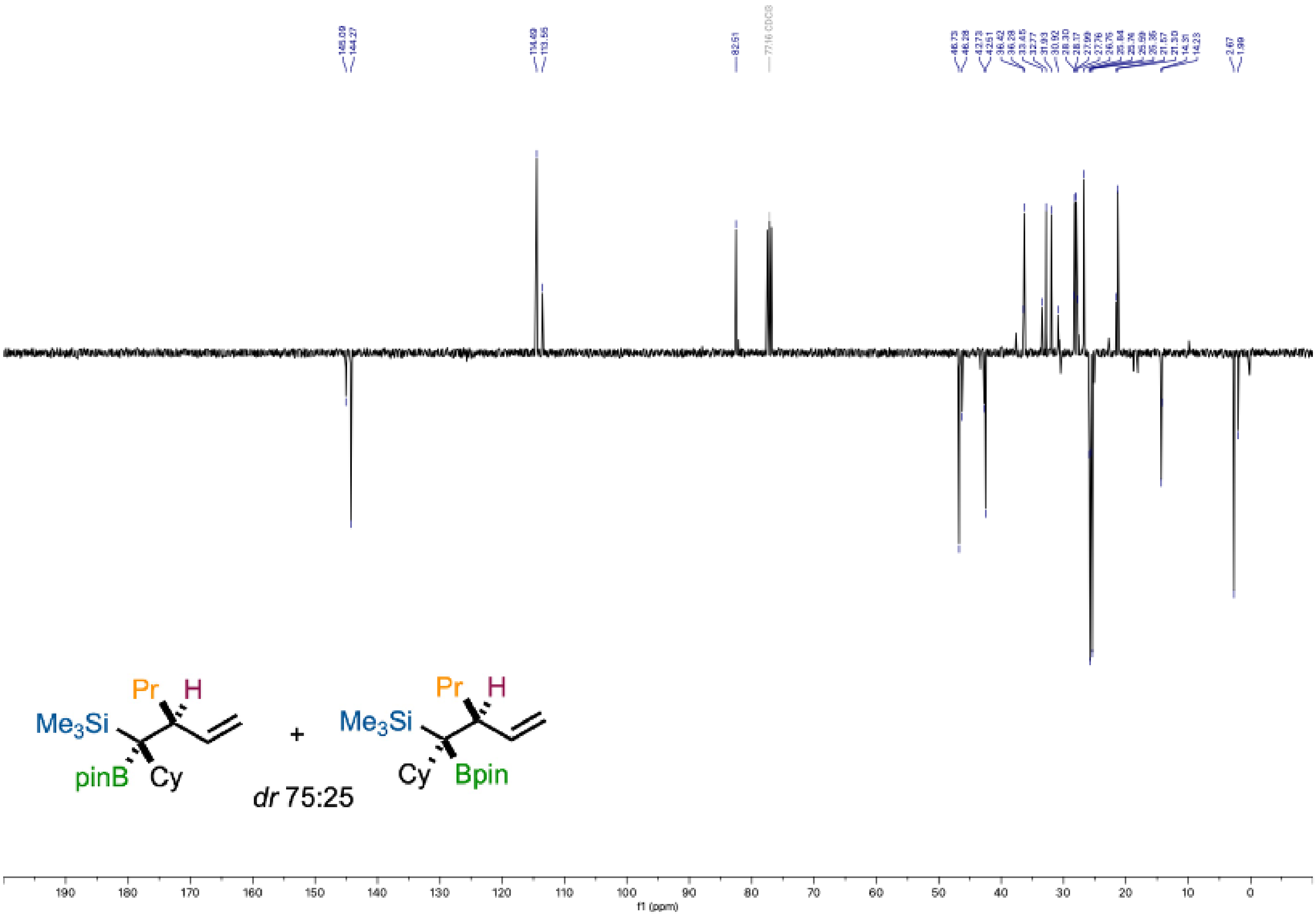

^{13}C NMR spectrum (101 MHz, $CDCl_3$)

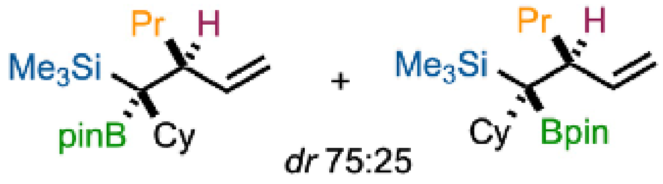

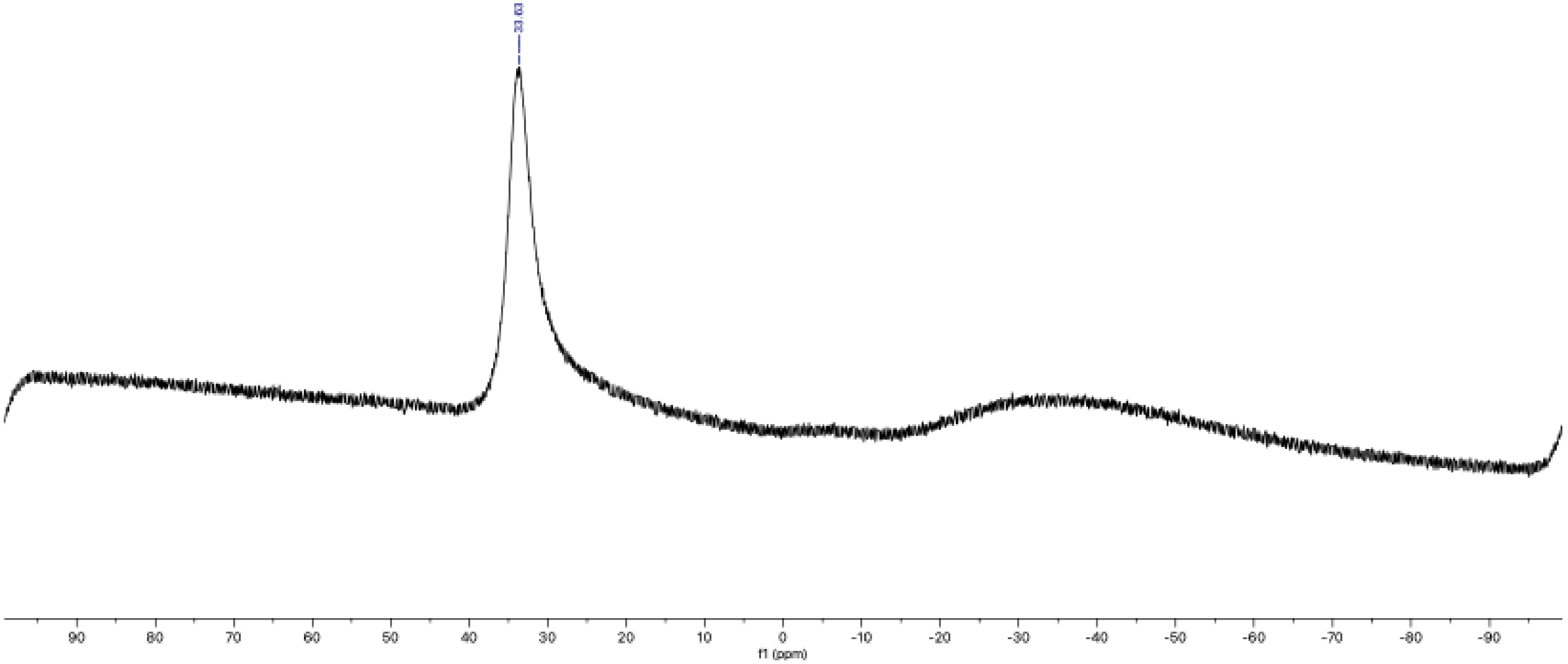

^{11}B NMR spectrum (128 MHz, $CDCl_3$)

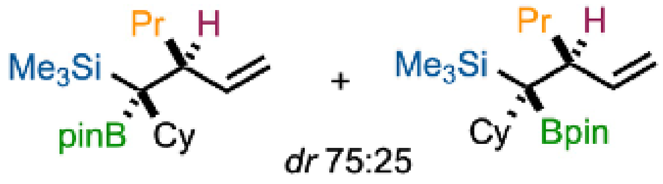

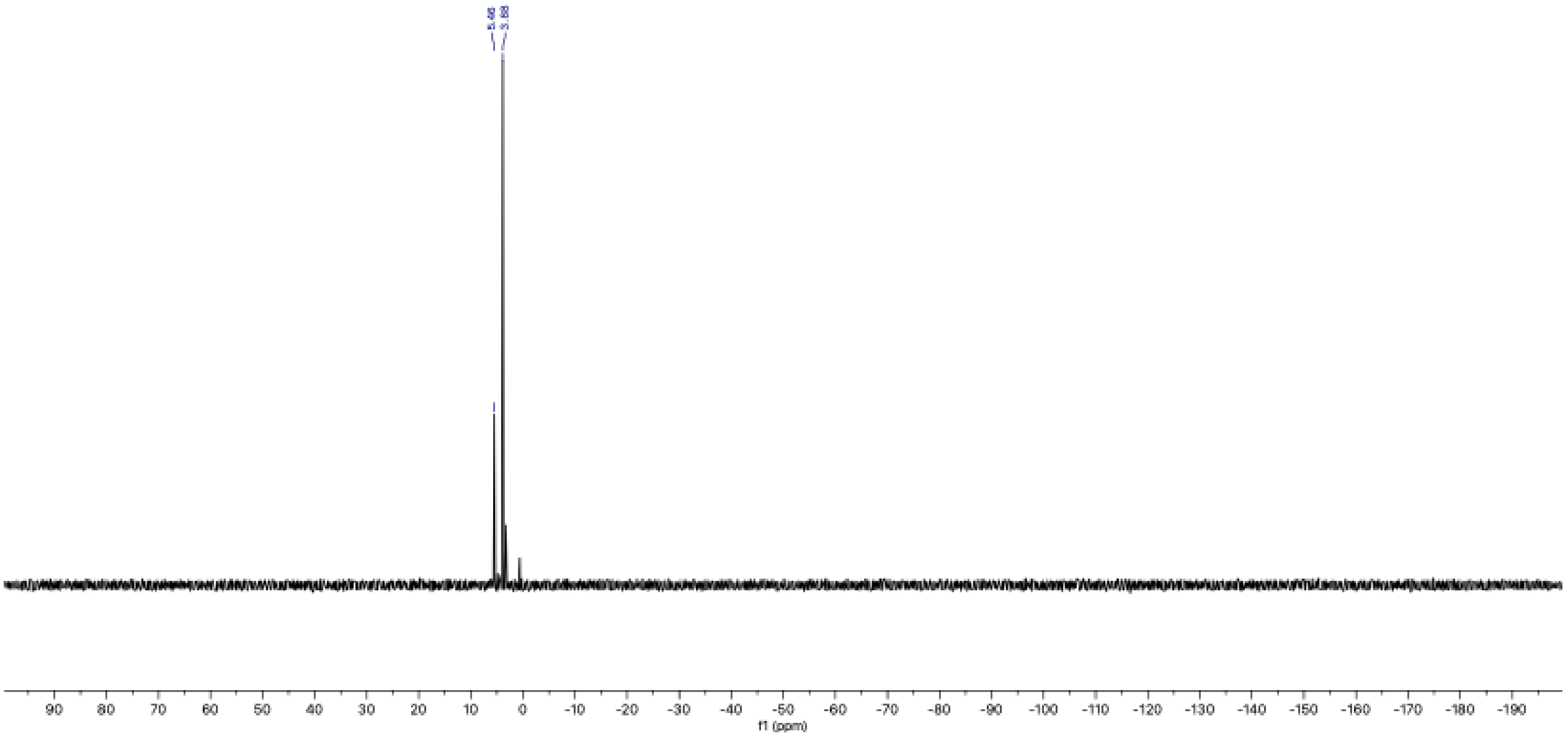

^{29}**Si NMR** spectrum (80 MHz, $CDCl_3$)

Trimethyl((2*S*,3*S*)-3-methyl-2-(4,4,5,5-tetramethyl-1,3,2-dioxaborolan-2-yl)-3-vinylhex-5-en-2-yl)silane (6h)

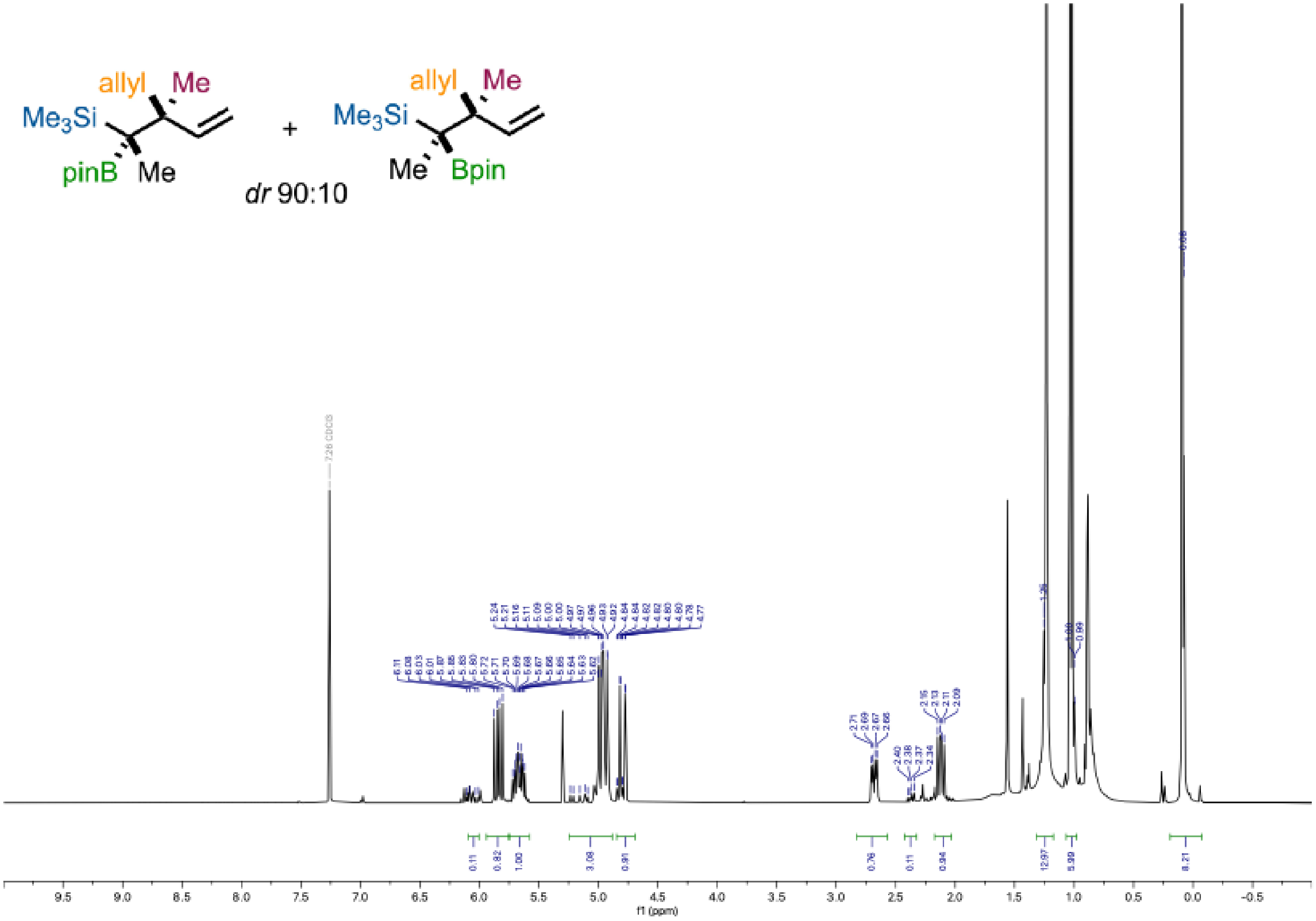

[1]**H NMR** spectrum (400 MHz, $CDCl_3$)

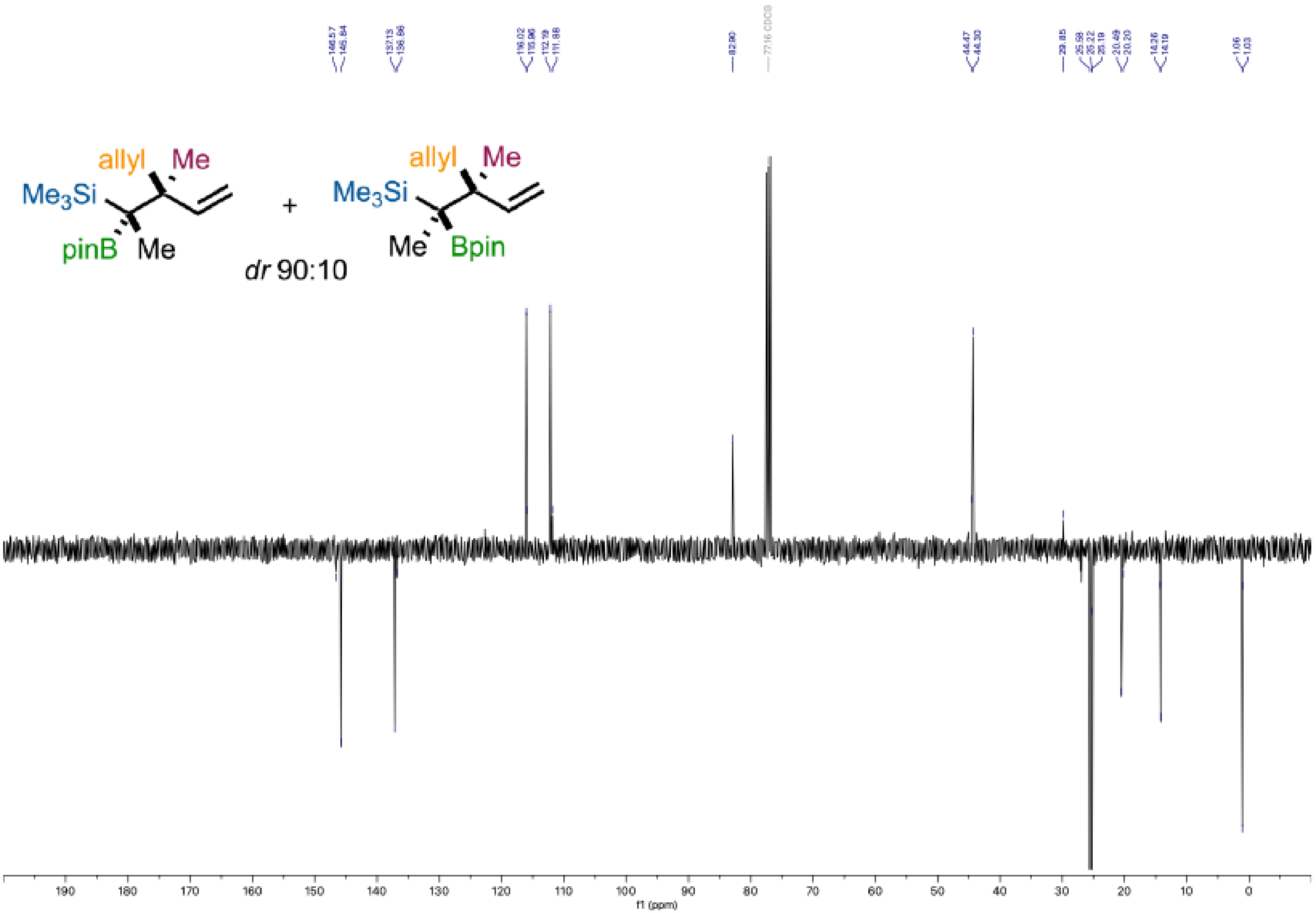

^{13}**C NMR** spectrum (101 MHz, $CDCl_3$)

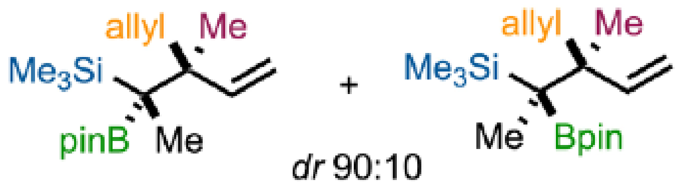

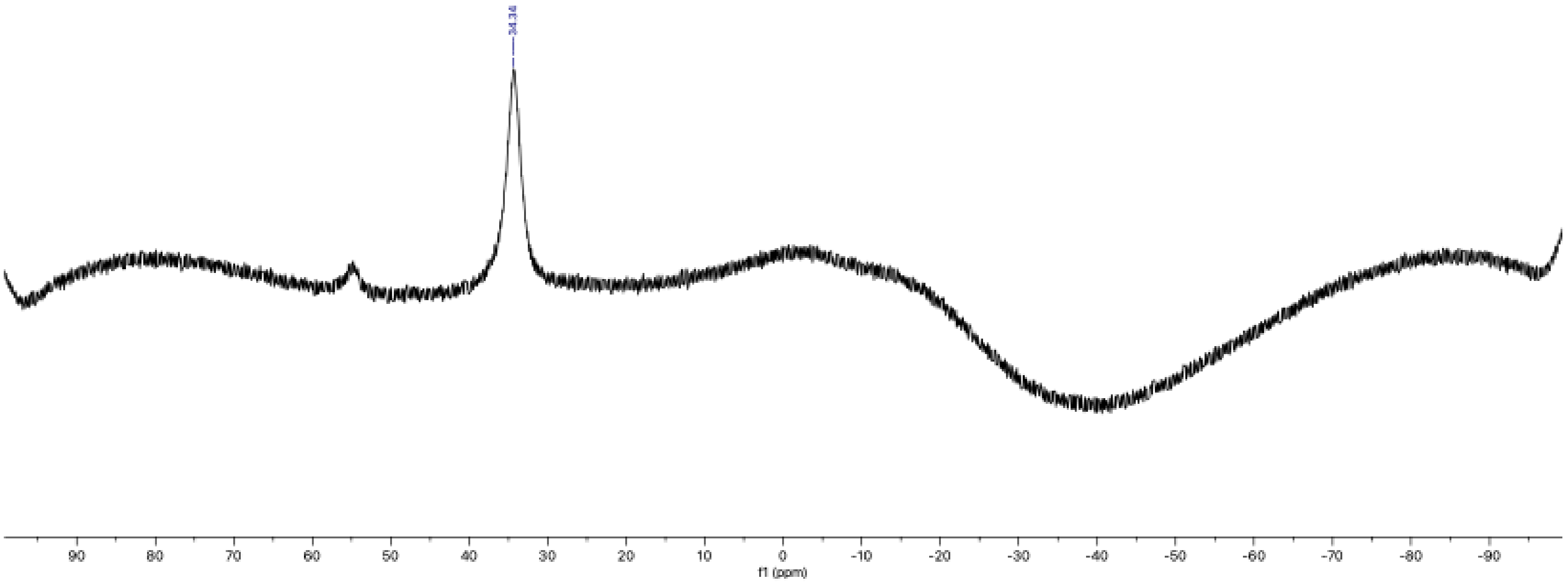

^{11}B NMR spectrum (128 MHz, $CDCl_3$)

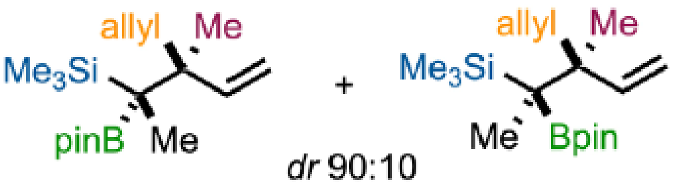

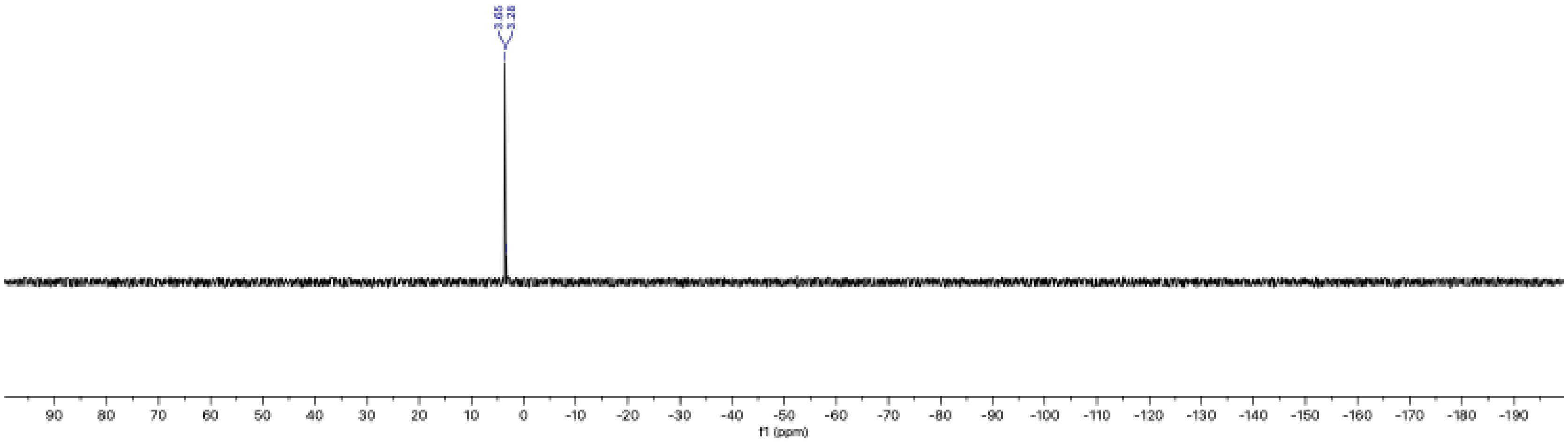

^{29}Si NMR spectrum (80 MHz, $CDCl_3$)

Tributyl((3*R,4*R**)-4-ethyl-3-(4,4,5,5-tetramethyl-1,3,2-dioxaborolan-2-yl)hex-5-en-3-yl)stannane (6i)**

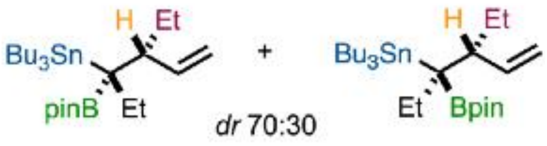

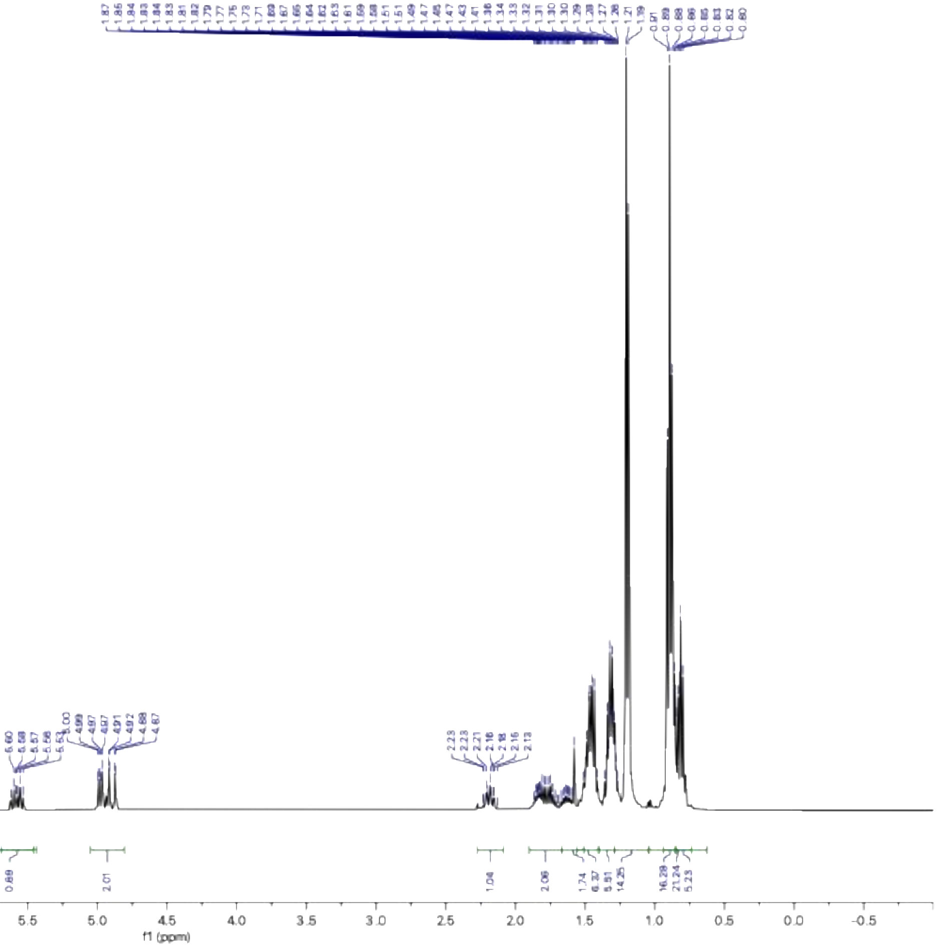

^{1}H NMR spectrum (400 MHz, $CDCl_3$)

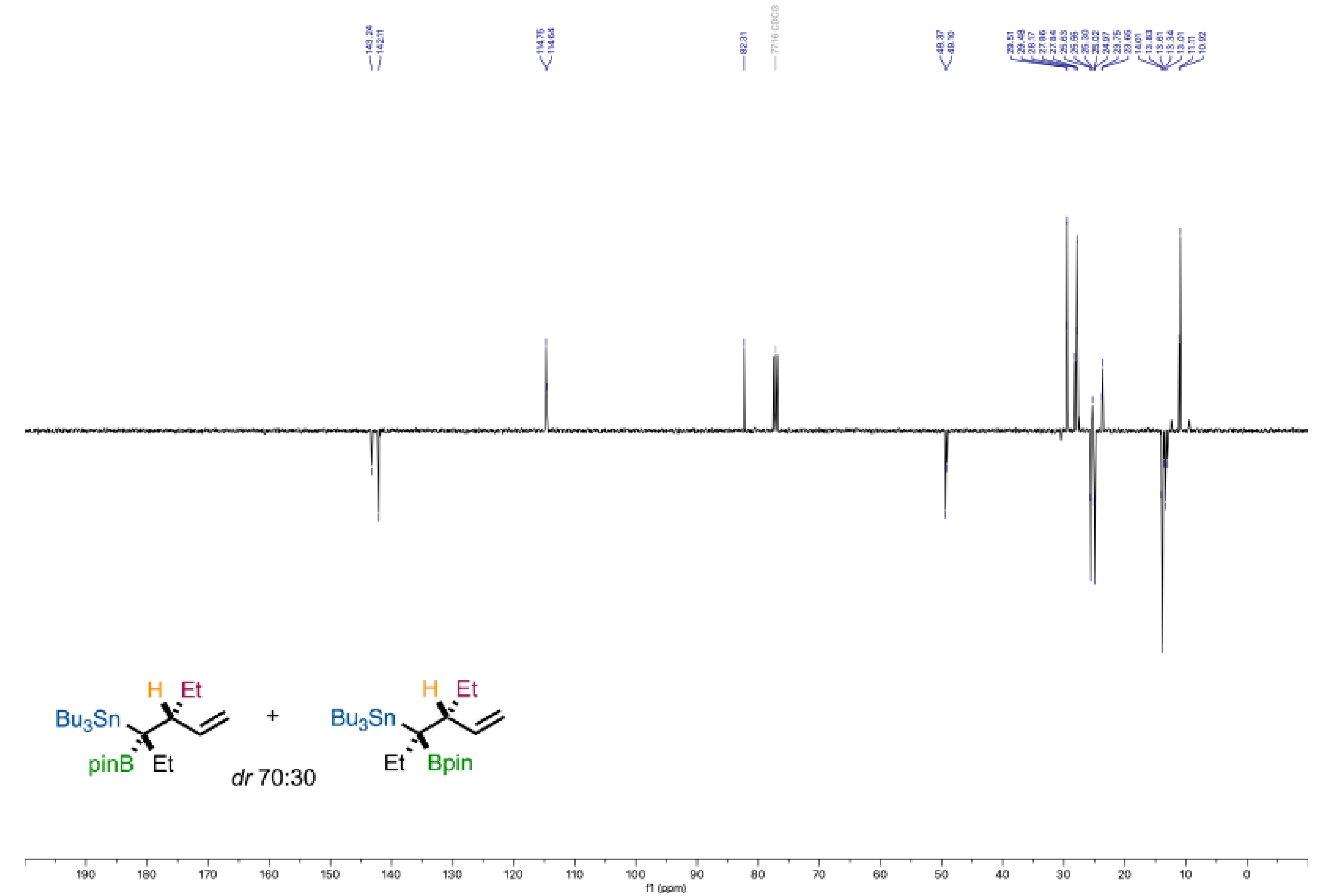

^{13}C NMR spectrum (101 MHz, $CDCl_3$)

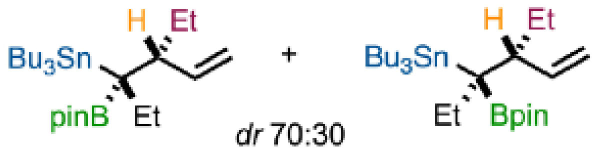

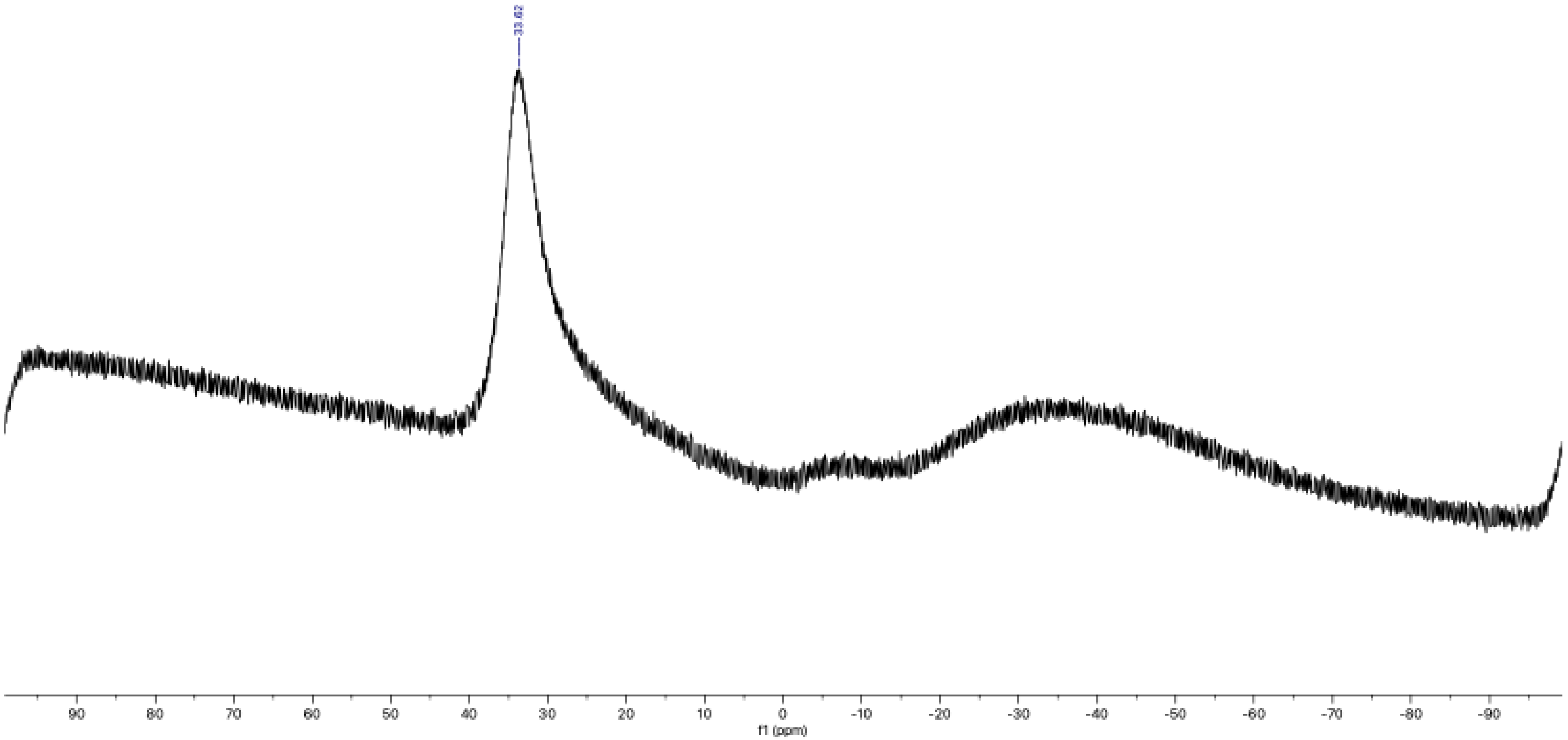

^{11}B NMR spectrum (128 MHz, $CDCl_3$)

(4-Ethylhex-5-en-3-ylidene)cyclohexane (6j)

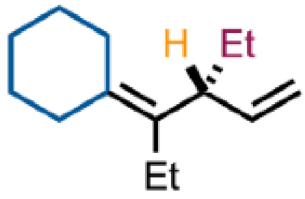

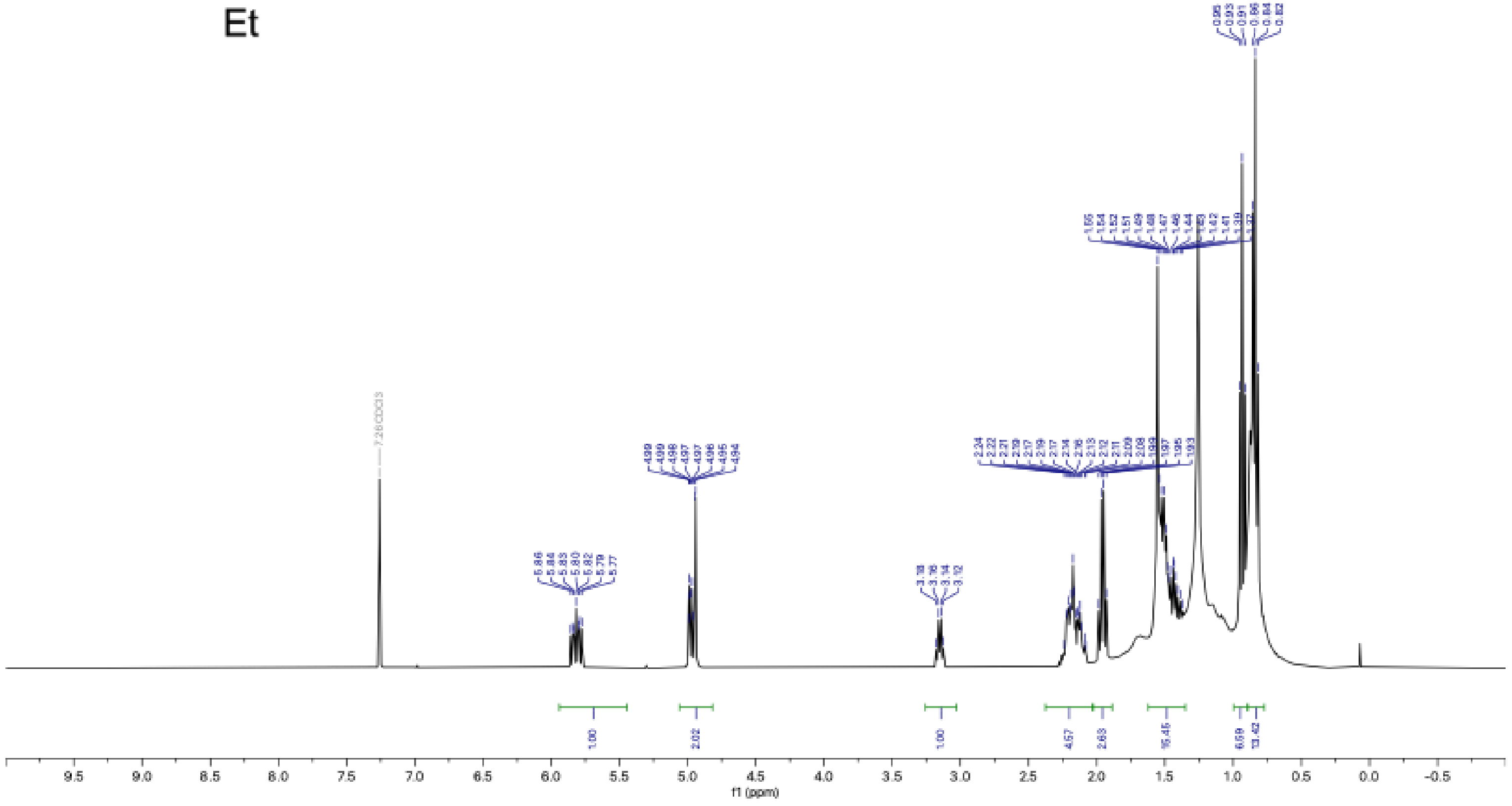

[1]**H NMR** spectrum (400 MHz, $CDCl_3$)

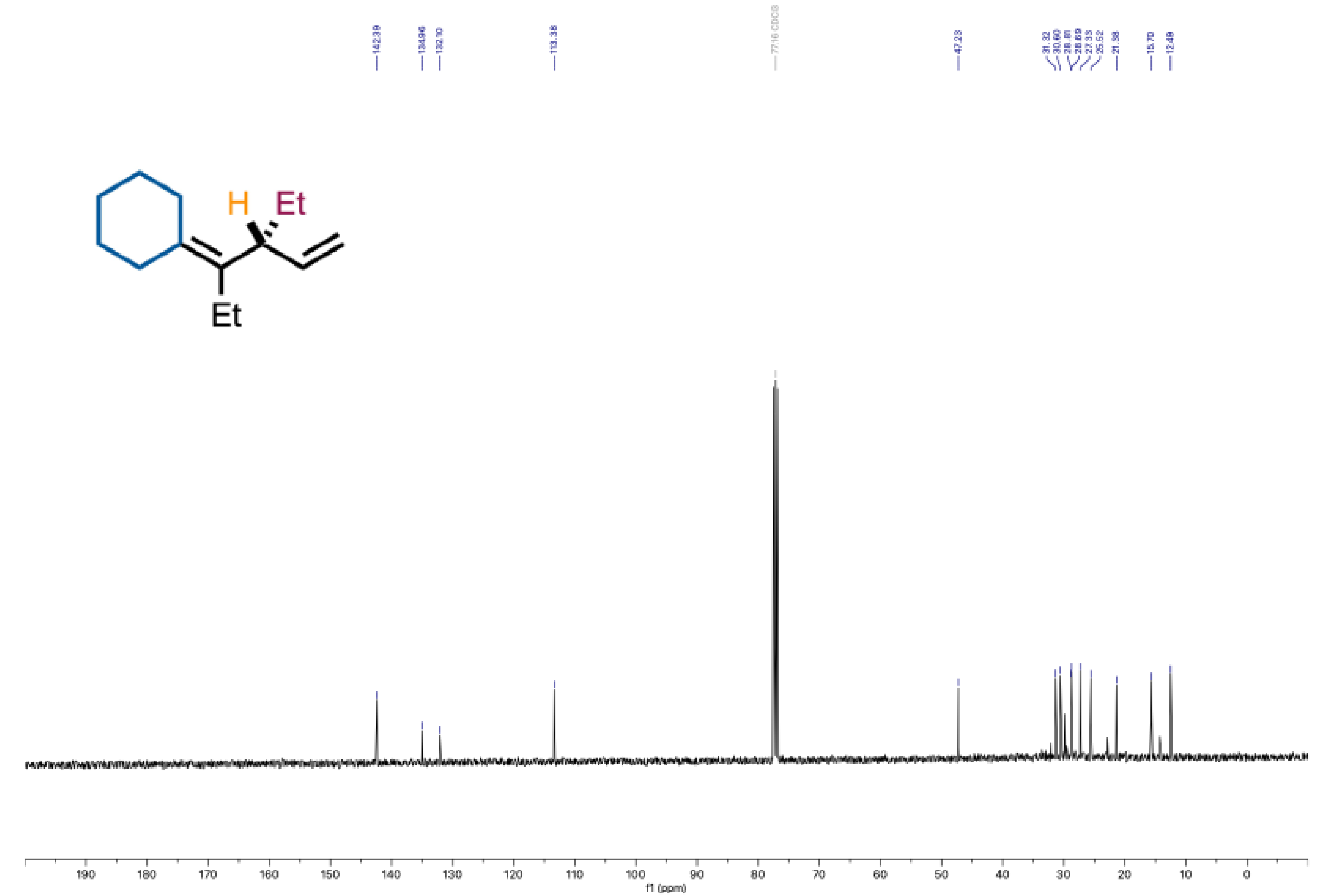

^{13}C NMR spectrum (101 MHz, $CDCl_3$)

2-((1*R,2*S**)-1-Butyl-2-vinylcyclopentyl)-4,4,5,5-tetramethyl-1,3,2-dioxaborolane (6k)**

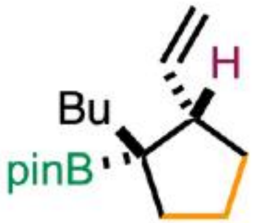

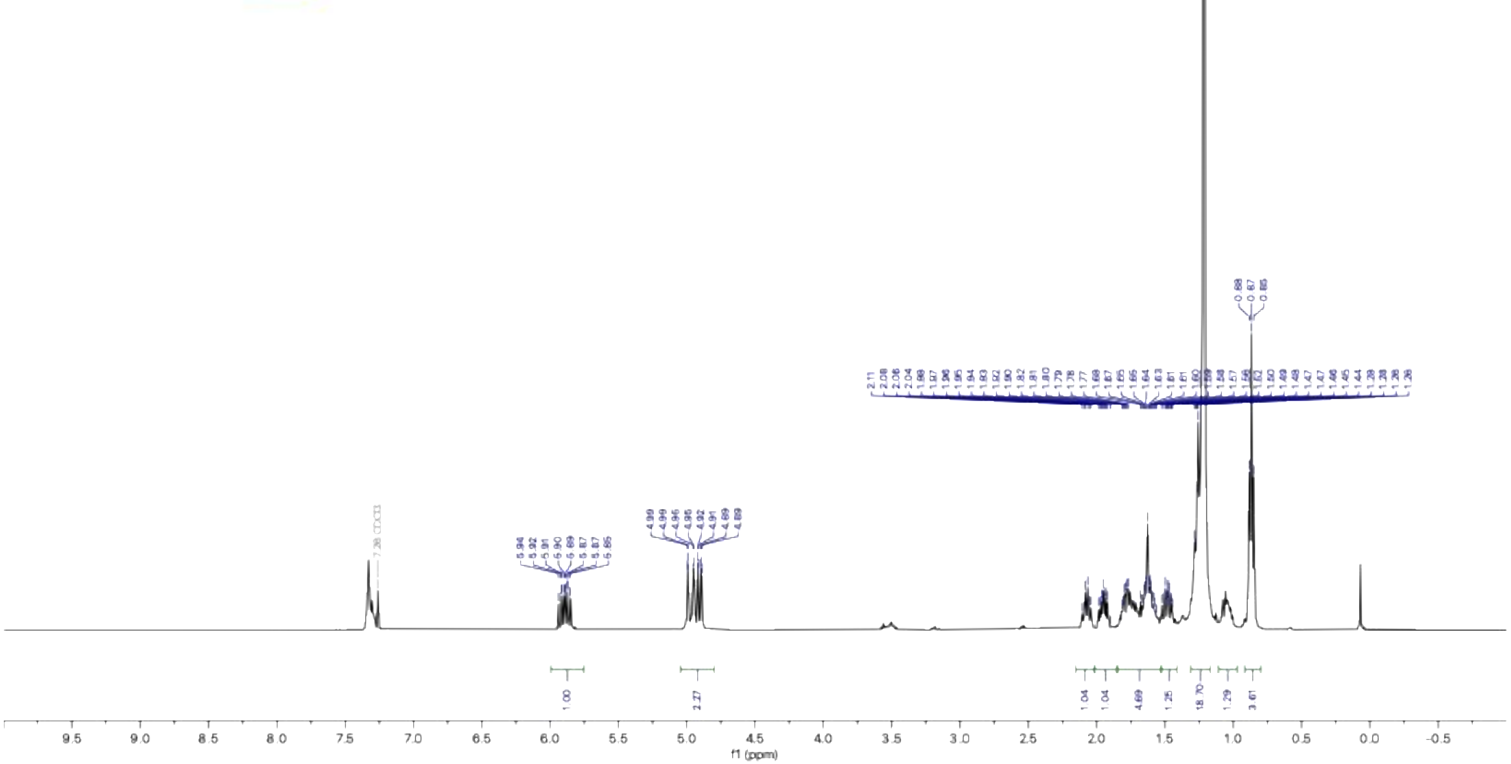

^{1}H NMR spectrum (400 MHz, $CDCl_3$)

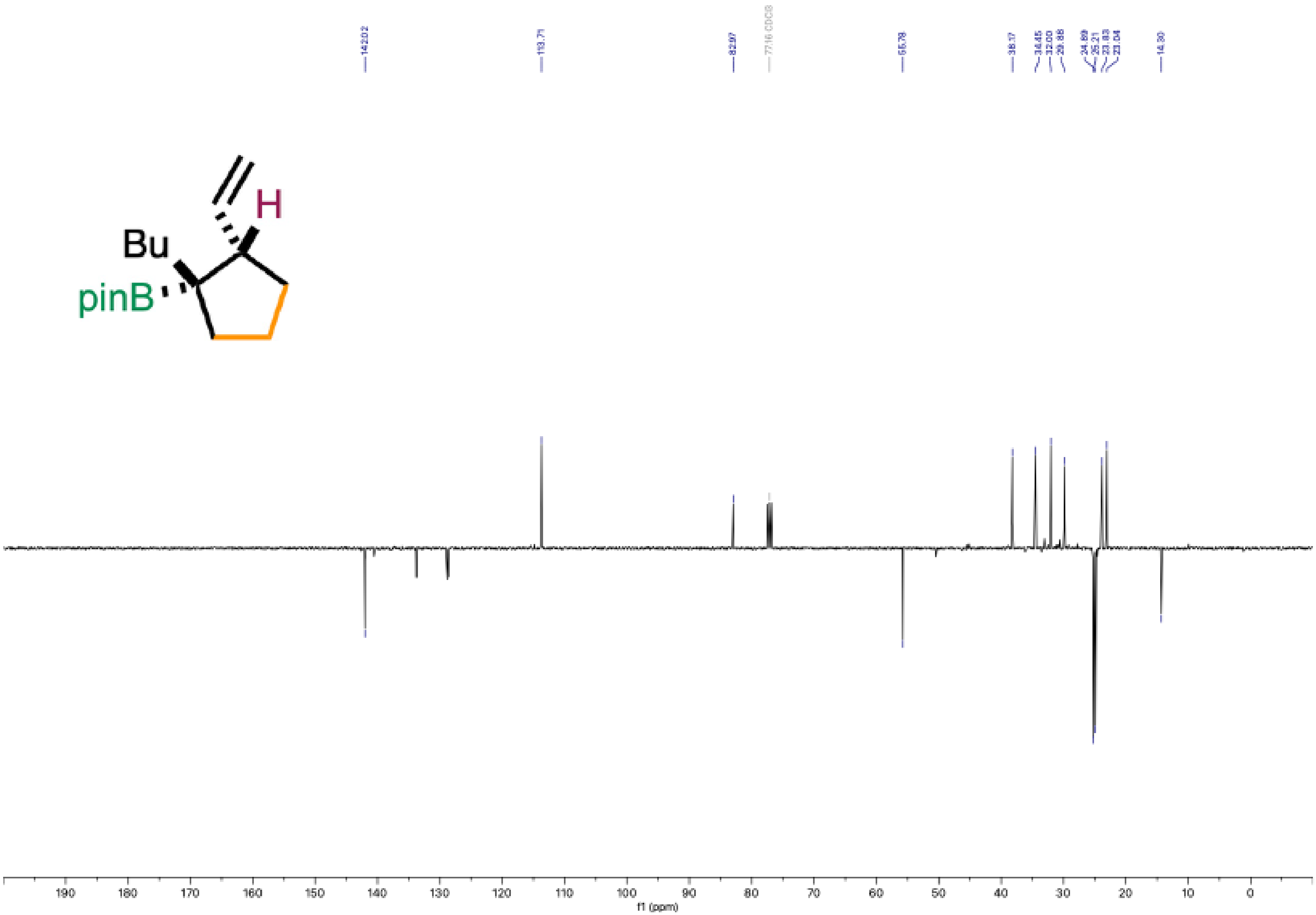

^{13}C NMR spectrum (101 MHz, $CDCl_3$)

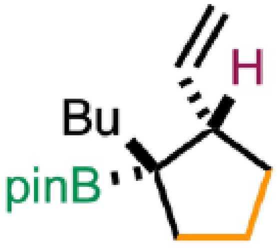

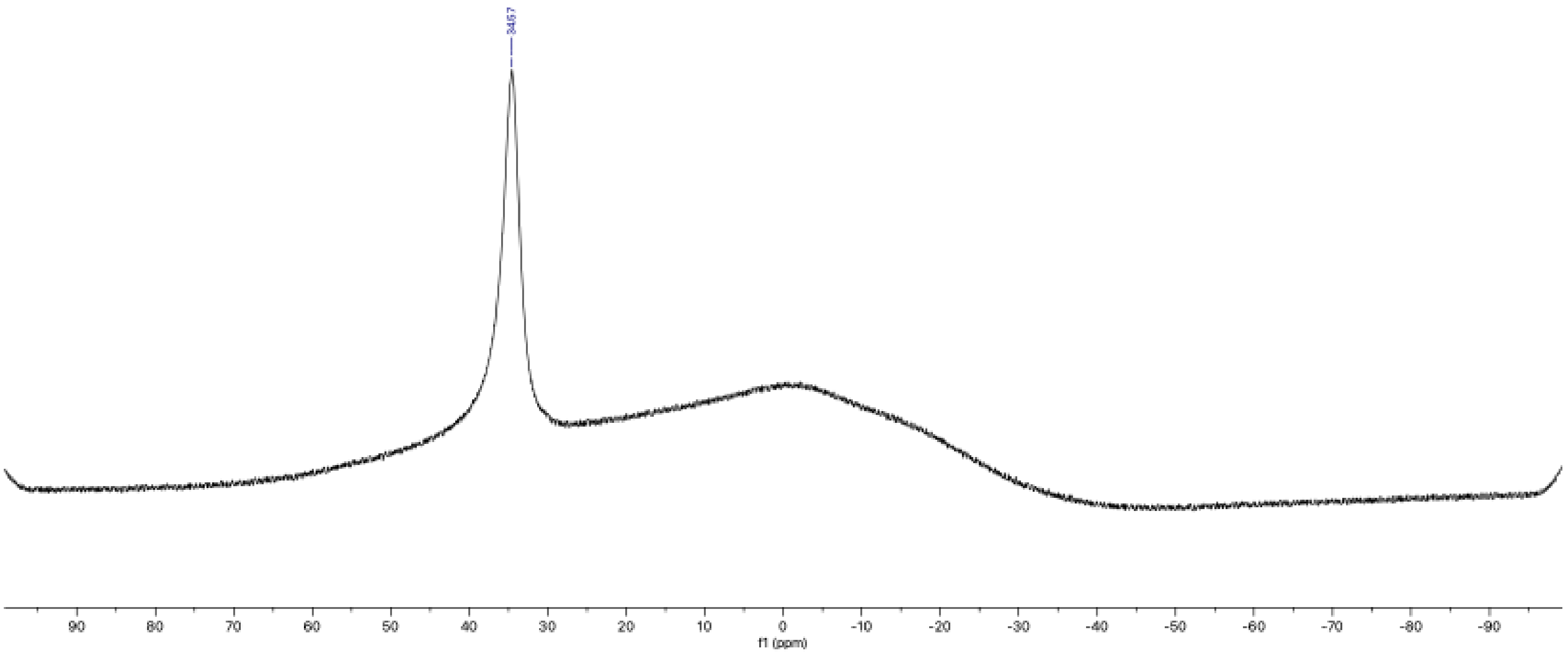

^{11}B NMR spectrum (128 MHz, $CDCl_3$)

Dioxo[*N*,*N*,*N*,*N*-tetramethylethane-1,2-diaminetetramethylehylendiamine][(2*R,3*R**,4*R**)-4-(dimethyl(phenyl)silyl)-3-methyl-4-(4,4,5,5-tetramethyl-1,3,2-dioxaborolan-2-yl)pentane-1,2-diol]osmium complex 7b**

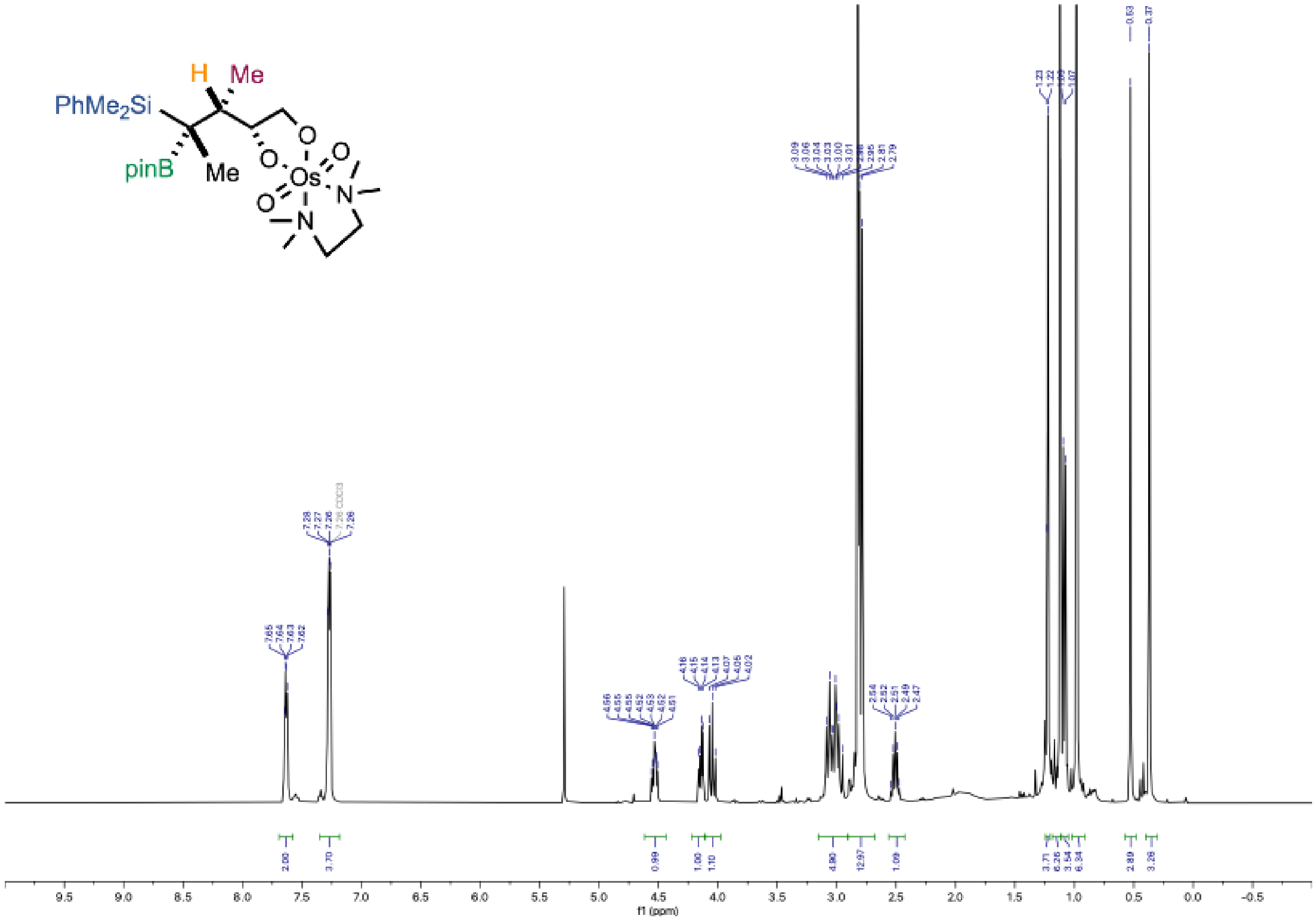

[1]**H NMR** spectrum (400 MHz, $CDCl_3$)

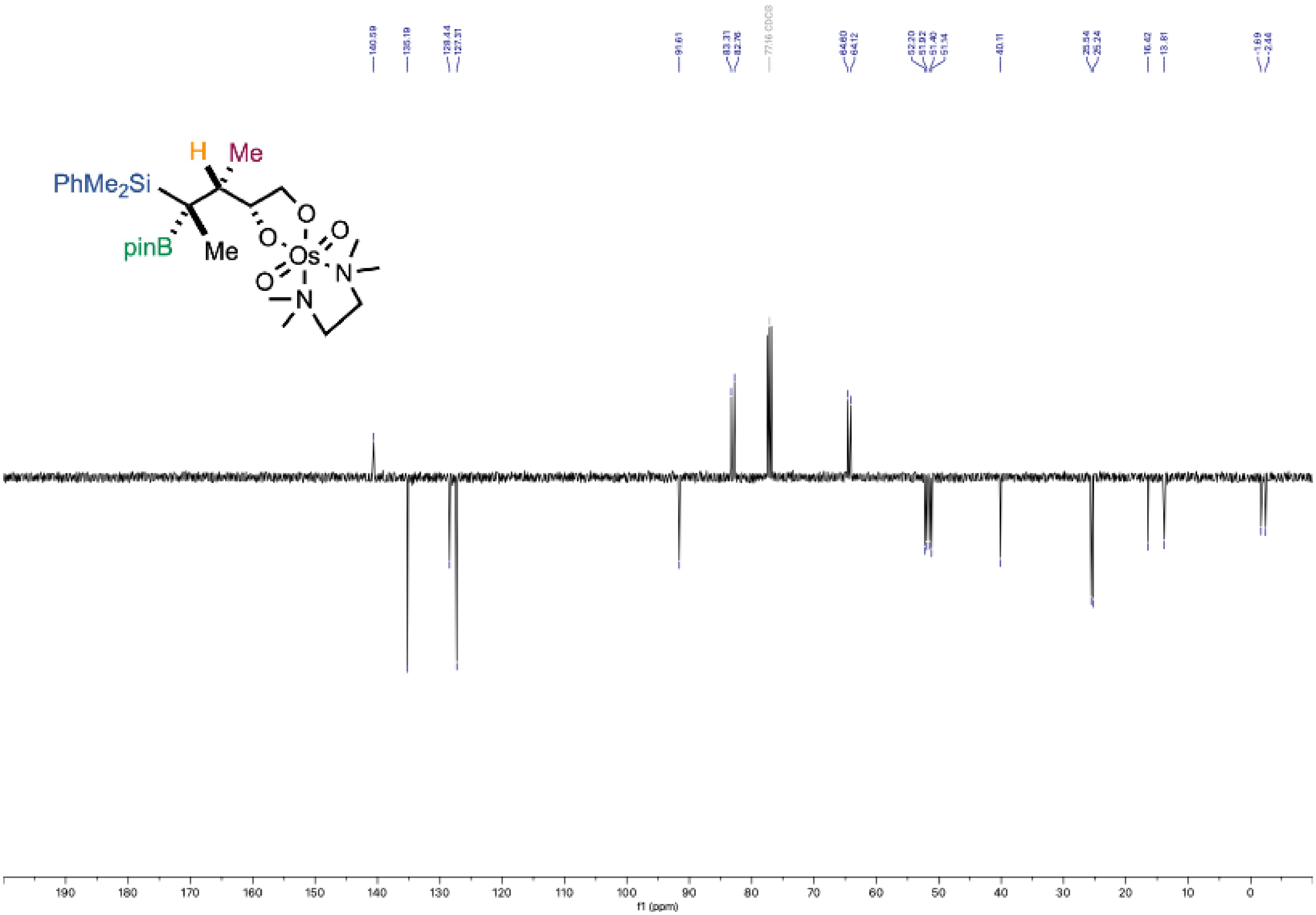

^{13}C NMR spectrum (101 MHz, $CDCl_3$)

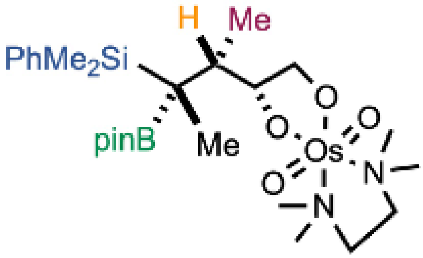

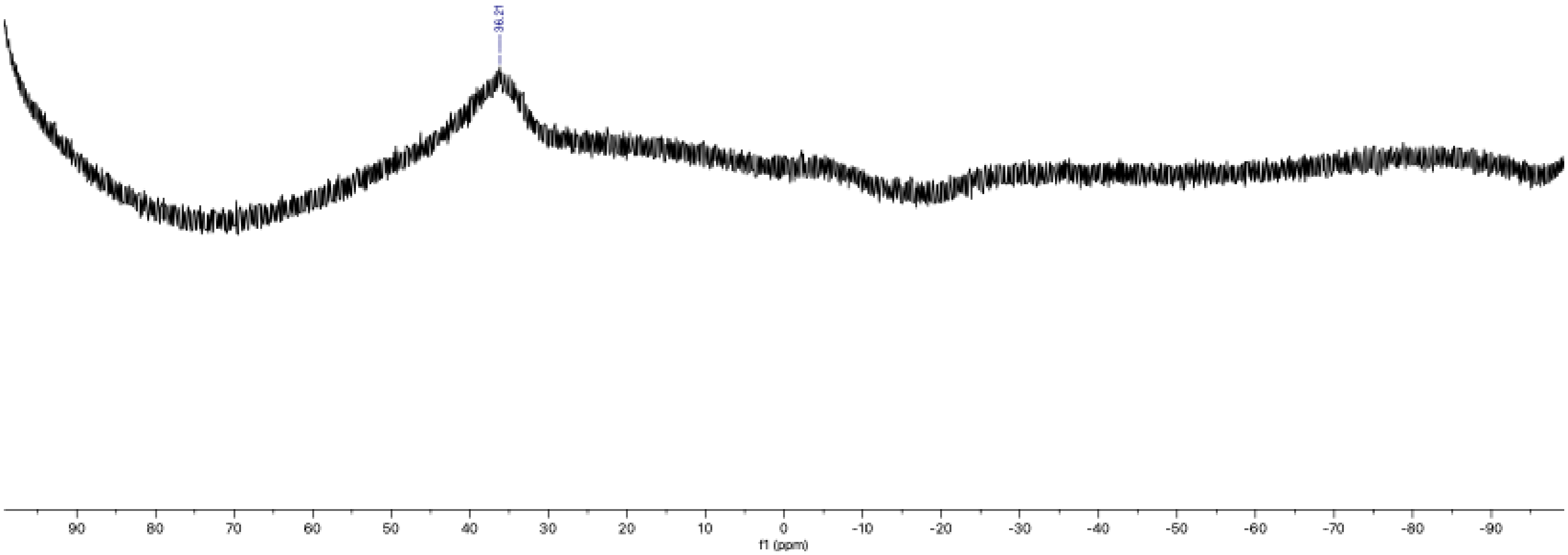

^{11}B NMR spectrum (128 MHz, $CDCl_3$)

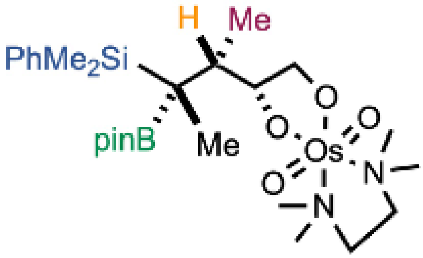

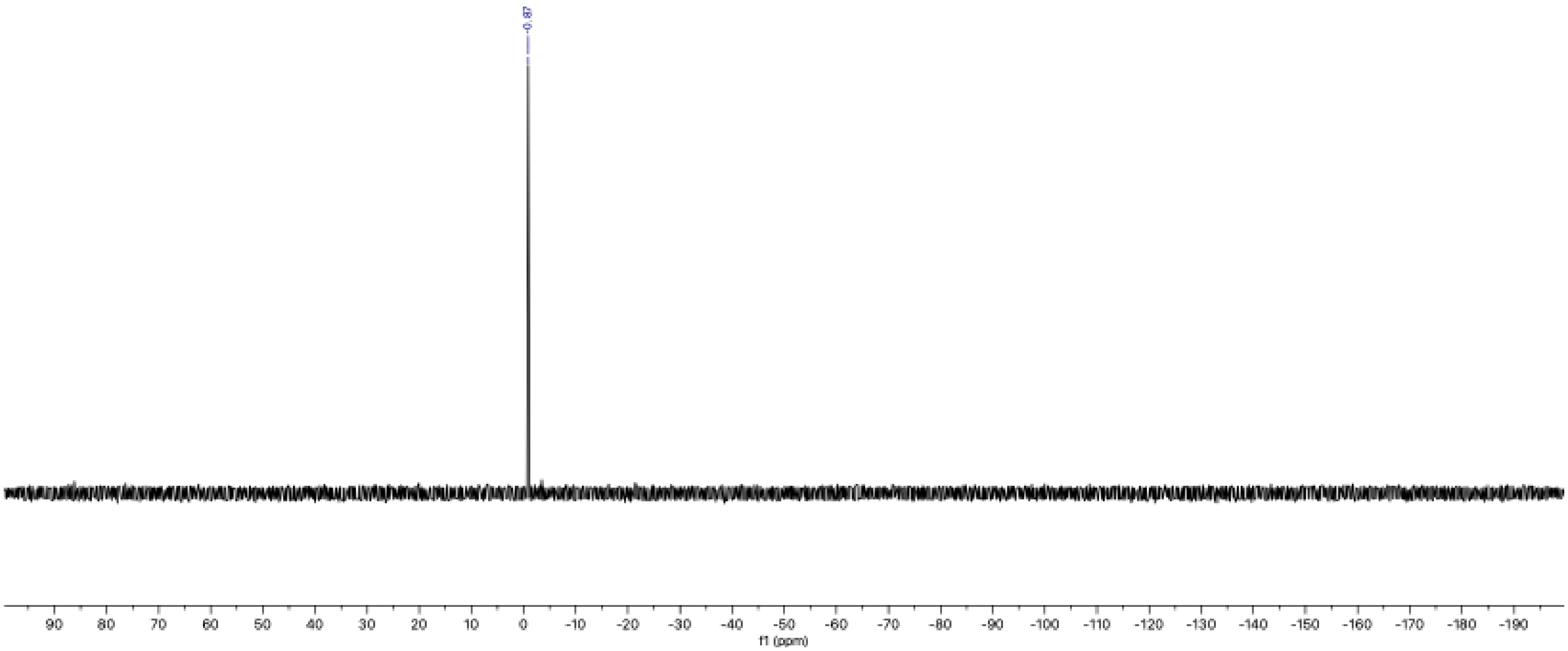

[29]**Si NMR** spectrum (80 MHz, $CDCl_3$)

Dioxo[*N*,*N*,*N*,*N*-tetramethylethane-1,2-diaminetetramethylehylendiamine][(1-((1*R,2*R**)-2-butyl-2-(4,4,5,5-tetramethyl-1,3,2-dioxaborolan-2-yl)cyclopentyl)ethane-1,2-diol]osmium complex 7k**

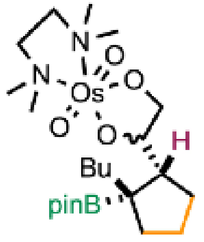

dr 75:25

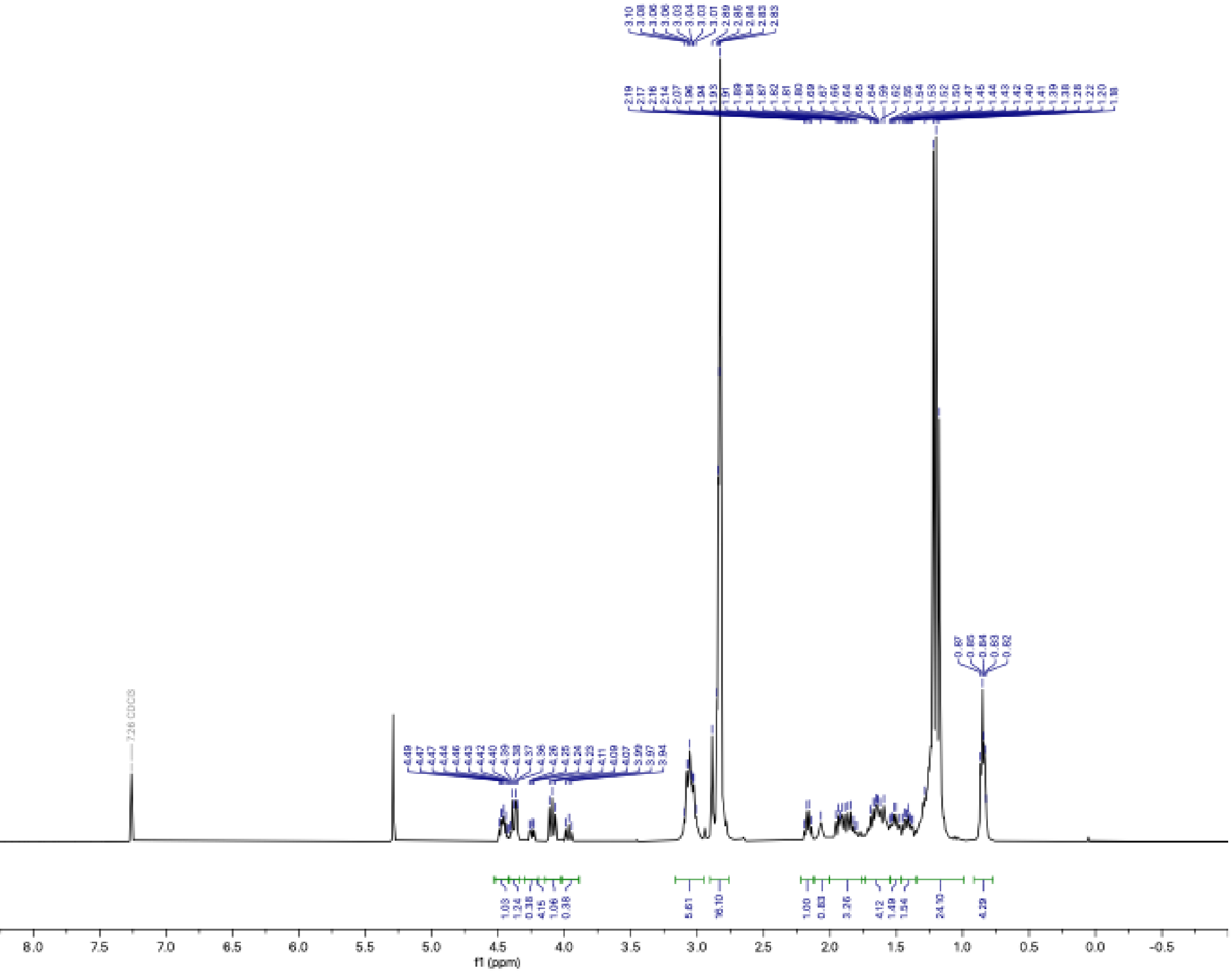

^{1}H NMR spectrum (400 MHz, $CDCl_3$)

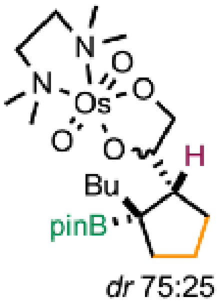

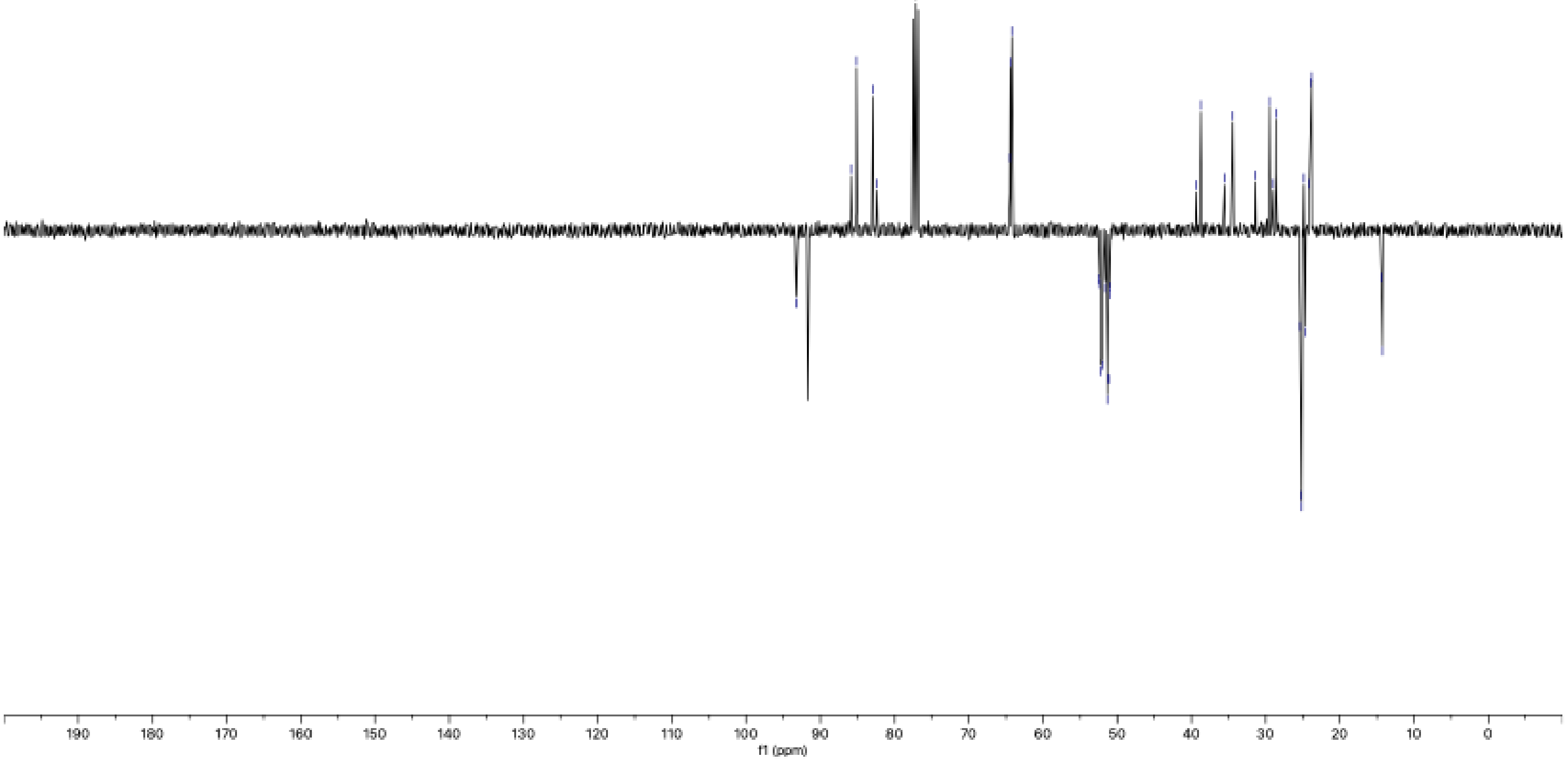

^{13}C NMR spectrum (101 MHz, $CDCl_3$)

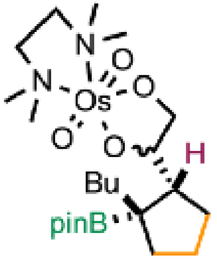

dr 75:25

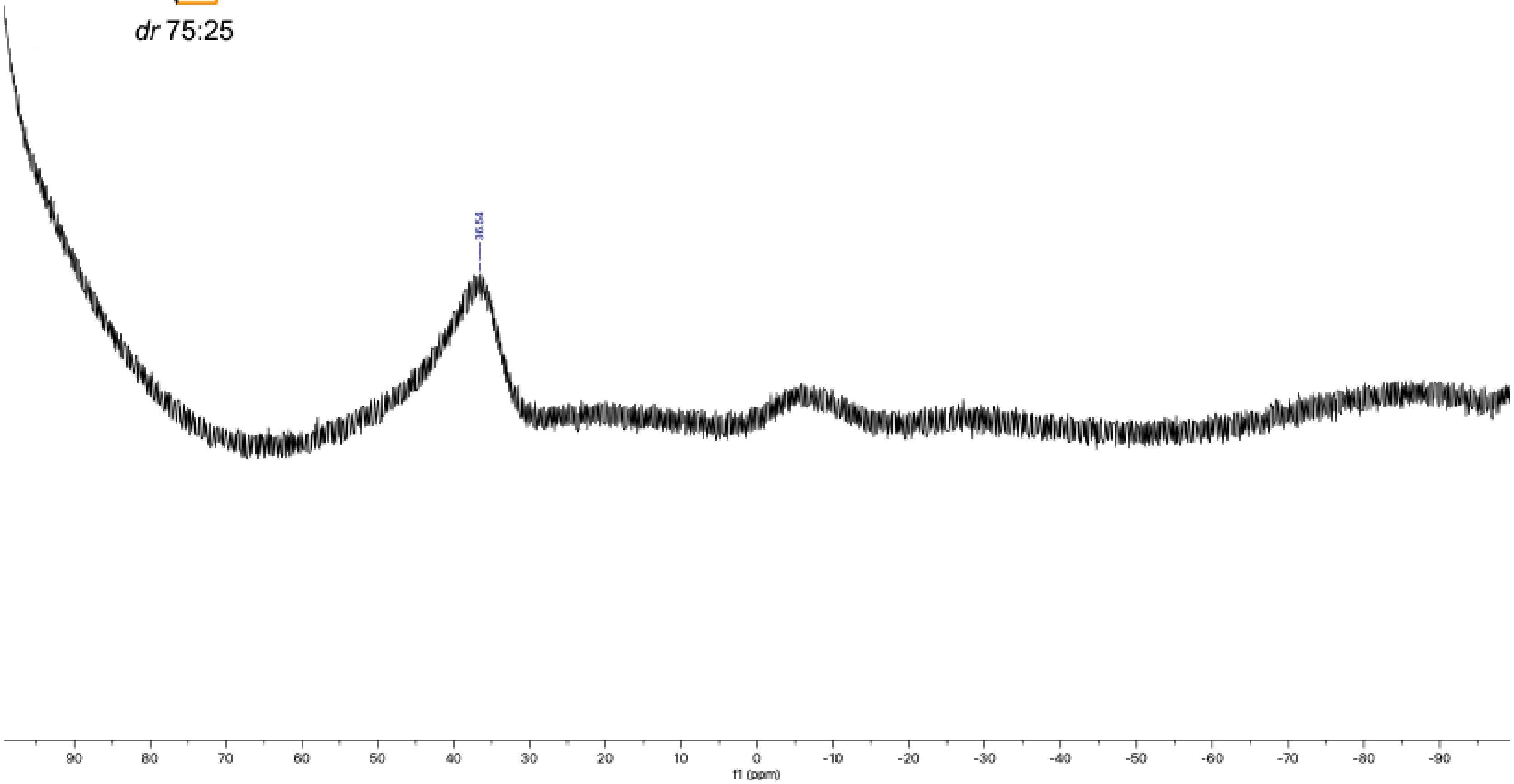

^{11}B NMR spectrum (128 MHz, $CDCl_3$)

6. References

(1) Augustin, A. U.; Di Silvio, S.; Marek, I. *J. Am. Chem. Soc.* **2022**, *144* (36), 16298-16302.
(2) Pavlíčková, T.; Stöckl, Y.; Marek, I. *Org. Lett.* **2022**, *24* (48), 8901-8906.
(3) Pavlickova, T.; Orbach, N.; Kaushansky, A.; Marek, I. *J. Am. Chem. Soc*. **2025,** *147*, 41204-41209.
(4) Burchat, A. F.; Chong, J. M.; Nielsen, N. *J. Organomet. Chem.* **1997**, *542* (2), 281-283.
(5) Burns, A. S.; Dooley III, C.; Carlson, P. R.; Ziller, J. W.; Rychnovsky, S. D. *Org. Lett*. **2019**, *21*, 10125–10129.
(6) Dolomanov, O. V.; Bourhis, L.J.; Gildea, R. J.; Howard, J. A. K.; Puschmann, H. *J. Appl. Cryst.* **2009**, *42*, 339-341.
(7) Bourhis, L. J.; Dolomanov, O. V.; Gildea, R. J;, Howard, J. A. K.; Puschmann, H. *Acta Cryst.* **2015,** *A71*, 59-75.
(8) Sheldrick, G.M. *Acta Cryst.* **2015**, *C71*, 3-8.
(9) Mercury Software from CCDC: http://www.ccdc.cam.ac.uk/ Solutions/CSDSystem/Pages/Mercury.aspx.
